# A Convenient Way to Quinoxaline Derivatives through the Reaction of 2-(3-Oxoindolin-2-yl)-2-phenylacetonitriles with Benzene-1,2-diamines

**DOI:** 10.3390/ijms231911120

**Published:** 2022-09-22

**Authors:** Alexander V. Aksenov, Nikolai A. Arutiunov, Dmitrii A. Aksenov, Artem V. Samovolov, Igor A. Kurenkov, Nicolai A. Aksenov, Elena A. Aleksandrova, Daria S. Momotova, Michael Rubin

**Affiliations:** 1 Department of Chemistry, North Caucasus Federal University, 1a Pushkin St., 355017 Stavropol, Russia; 2 Department of Chemistry, University of Kansas, 1567 Irving Hill Road, Lawrence, KS 66045, USA

**Keywords:** cyclization, quinoxalines, microwave synthesis

## Abstract

Microwave-assisted reaction between 2-(3-oxoindolin-2-yl)-2-phenylacetonitriles andbenzene-1,2-diamines leads to the high-yielding formation of the corresponding quinoxalines as sole, easily isolaable products. The featured transformation involves unusual extrusion of phenylacetonitrile molecule and could be performed in a short sequence starting from commonly available indoles and nitroolefins.

## 1. Introduction

**Scheme 1:**
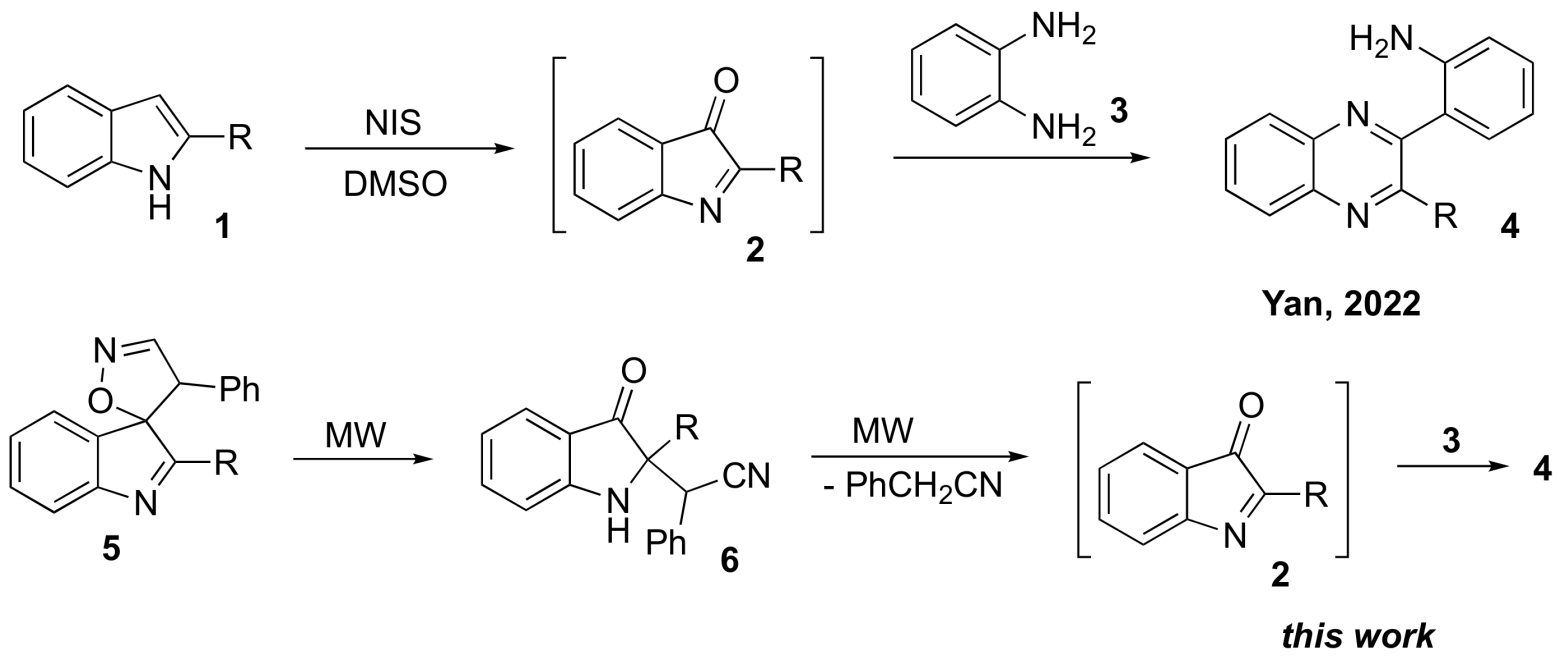
Direct assembly of 2-(quinoxalin-2-yl)anilines via ring-opening/cyclization of indoles with 1,2-diaminoarenes.

**Scheme 2:**
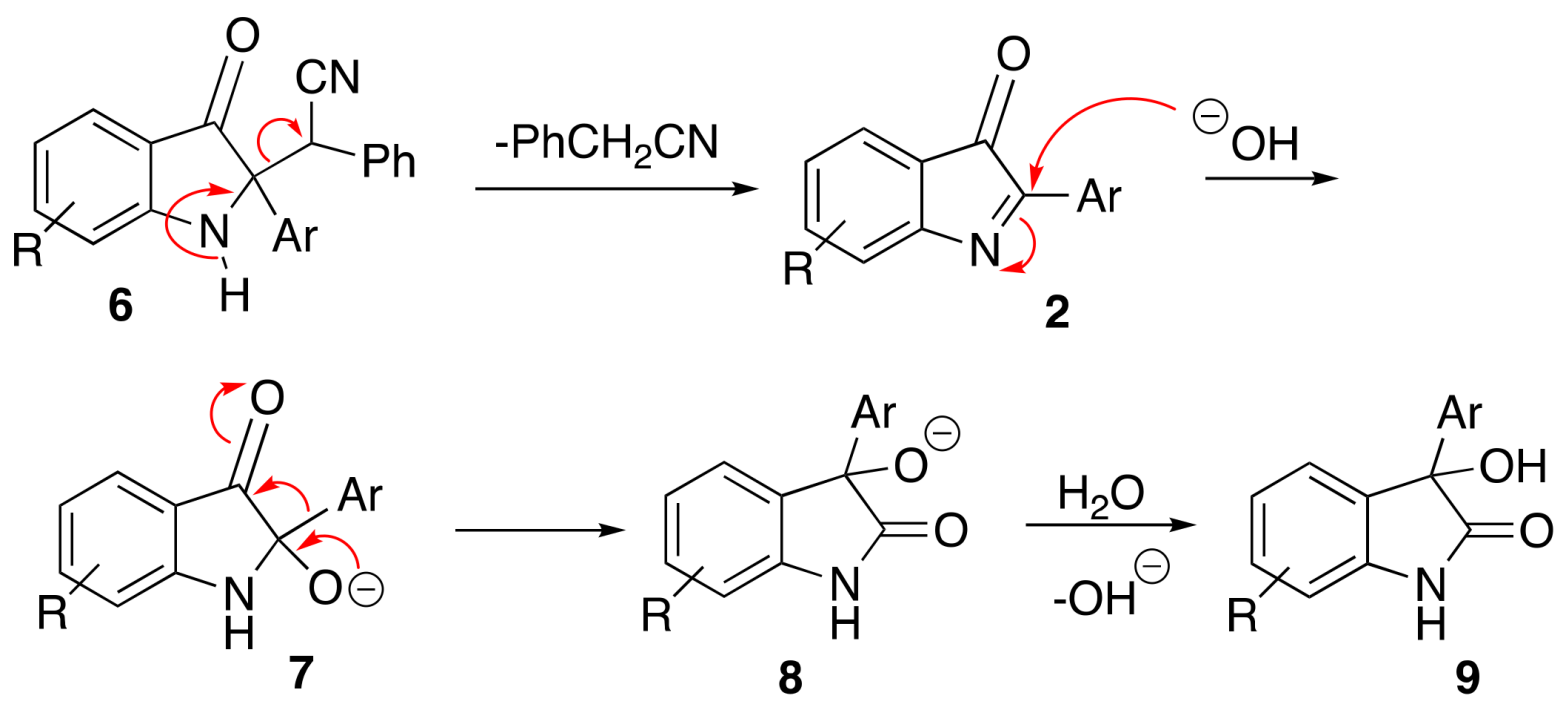
Extrusion of phenylacetonitrile molecule from 2-(3-oxoindolin-2-yl)-2-arylacetonitriles **6** to generate 3-hydroxyindolin-2-ones **9**.

It is hard to overstate the importance of quinoxalines for modern drug discovery and medicinal chemistry [1,2,3,4,5]. Many quinoxaline-based natural products have shown a broad range of bioactivities [6,7] and naturally, this heterocyclic core is considered one of the privileged pharmacophoric scaffolds for drug design [8]. In some cases, those compounds were successfully employed as efficient fluorescent probes used in molecular electronics, analytical chemistry, and the design of photo-triggered medicines [9,10,11] while quinoxalines **4** bearing ortho-aniline moiety at C-2 (or their closely related derivatives) has attracted great attention as selective DNA triple- and quadruple-helix intercalating ligands [12,13,14,15,16,17]. Nowadays, many synthetic approaches to these structures have been developed, although most of them rely on multi-step functional group transformation sequences. To the best of our knowledge, there is only one direct approach to structure **4** reported recently by Yan et al. [18] that involves oxidative ring-opening/cyclization cascade of indoles **1** with the 1,2-diaminoarenes **3**; this rather elegant method relays on initial oxidation of indole with NIS in DMSO to obtain 3*H*-indol-3-one **2**, which undergoing subsequent ANRORC cascade with bis-nucleophilic species **3** (Scheme 1) [18]. In turn, we recently demonstrated that nitroolefins might act as 1,4-CCNO dipoles in reaction with indoles in the presence of phosphorous acid; this unusual transformation efficiently leads to the formation of stereo-defined spirocyclic scaffolds **5**, which are versatile and affordable synthetic equivalents of highly functionalized indoles [19,20]; it was shown that upon treatment with mild acids or bases as well as under neutral condition upon heating (Scheme 1) spiranes **5** could be diastereoselectively transformed into 2-(3-oxoindolin-2-yl)-2-arylacetonitriles **6** [21,22]. Further extrusion of 2-phenylacetonitrile molecule followed by the formation of postulated intermediate **2** was used by us to design cascade sequence involving 1,2-aryl shift and leading to 3-hydroxyindolin-2-ones **9** (Scheme 2) [23]. At some point, we speculated that such *in-situ* generated 3*H*-indol-3-one **2** in the presence of 1,2-diaminoarenes **3** could provide an alternative redox-neutral method for the preparation of quinoxalines **4** (Scheme 1) and below is our report on the development of this idea.

## 2. Results and Discussion

**Scheme 3:**
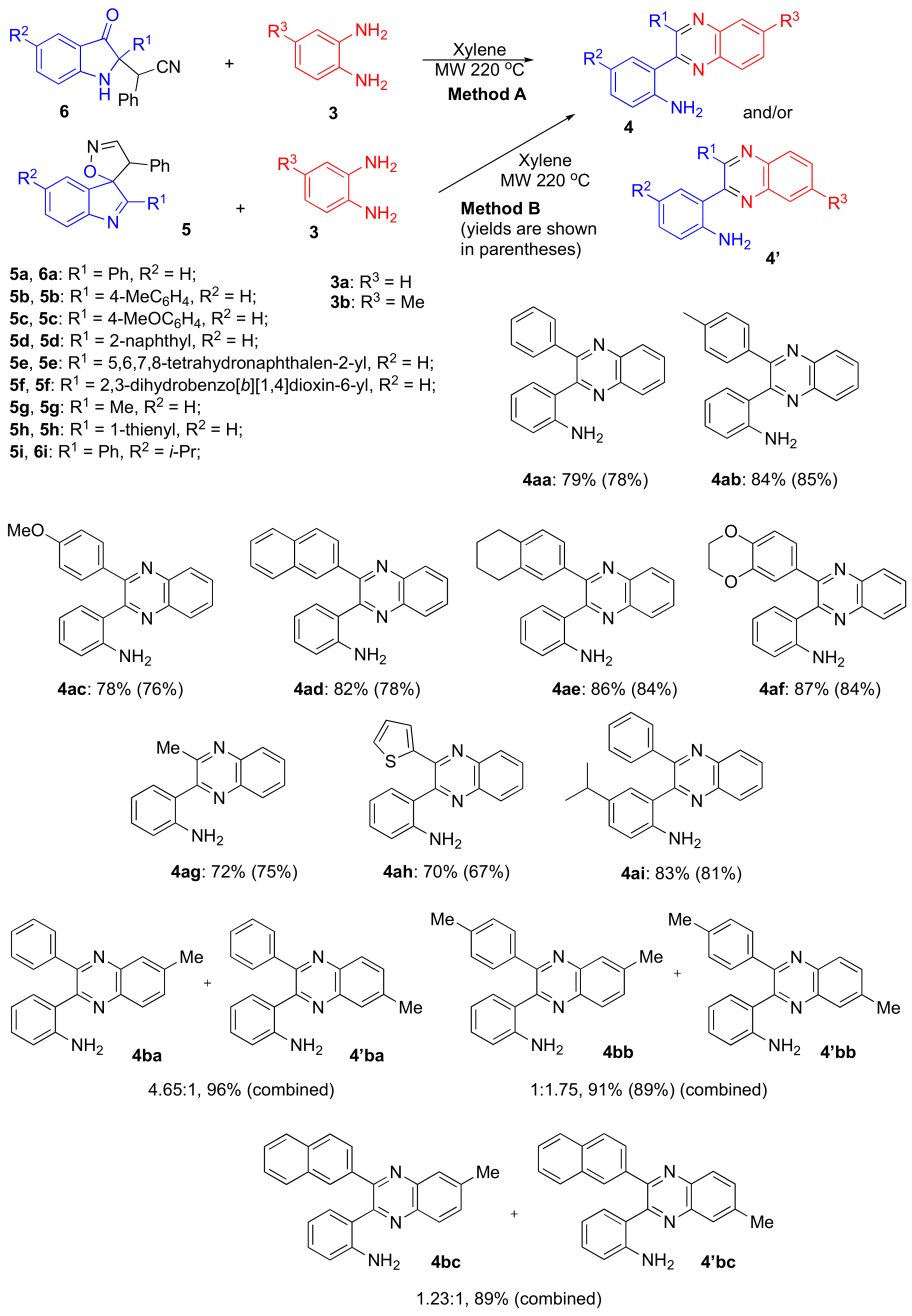
Preparation of quinoxalines via Methods A and B.

**Scheme 4:**
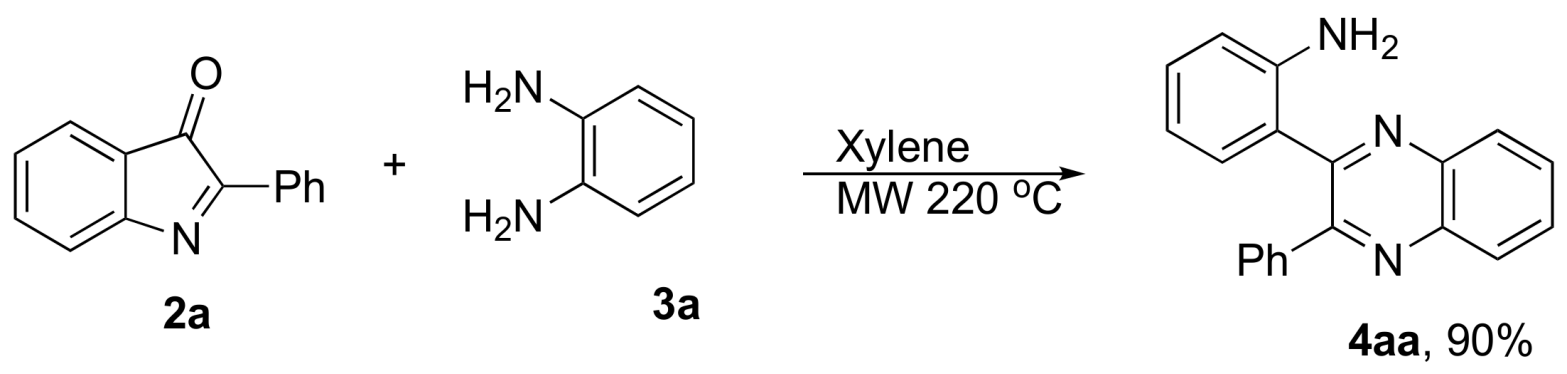
Generation of quinoxaline **4a** from 2-phenyl-3*H*-indol-3-one (**2a**) (Method C).

Earlier, we proposed a plausible base-assisted mechanism formation of 3-hydroxyindolin-2-one **9** from 2-(3-oxoindolin-2-yl)-2-arylacetonitriles **6** [23]. The extrusion of phenylacetonitrile molecule gives the intermediate 3*H*-indol-3-one **2** which, in turn, upon nucleophilic attack of hydroxide ion across C=N bond of the corresponding cyclic imine provides 3-oxoindolin-2-olate species **7** (Scheme 2). Next, the 1,2-aryl shift takes place affording isomeric 2-oxoindolin-3-olates **8** that, eventually, after protonation gives product **9** as sole isolable product. As the continuation of this research, we have decided to look into the possibility of generation of intermediate **2** under neutral conditions in the presence of 1,2-diaminoarenes **3** as the only available source of nucleophiles. In our opinion, this should lead to the formation of a corresponding quinoxaline core and, therefore, to the development of a novel, general procedure for the synthesis of quinoxaline derivatives. Testing this idea was began from the reaction between 2-(3-oxo-2-phenylindolin-2-yl)-2-phenylacetonitrile (**6a**, 1.00 mmol) and benzene-1,2-diamine (**3a**, 2.00 mmol) (Method A, Scheme 3). The reagents were dissolved in xylene (2 mL), and the solution was microwaved in a sealed tube at 220 °C for 1 h. Gladly, the reaction proceeded smoothly affording the target quinoxaline **4aa** with a high yield (Scheme 3). We also evaluated the possibility of direct conversion of spiranes **5** into quinoxalines and found out that under the same reaction conditions, 2,4’-diphenyl-4’*H*-spiro[indole-3,5’-isoxazole] (**5a**, 1.00 mmol) and diamine **3a** (2.00 mmol) gives the same product **4aa** in 78% yield (Method B, Scheme 3). Expectedly, 2-phenylacetonitrile was detected by GC/MS in aliquots of the crude reaction mixtures in both cases, thus confirming that reaction proceeds via extrusion of this molecule. Similarly, the independently synthesized 2-phenyl-3*H*-indol-3-one (**2a**) affords the same product **4aa** in 90% yield (Scheme 4) (or 74% yield when the reaction was performed in a 5.00 mmol scale—Method D).

Next, the scope and compatibility of the reaction were evaluated. For that, a series of acetonitriles **6** bearing various substituents R^1^ (including methyl, phenyl, substituted aryls, and thienyl) was introduced into the reaction with diamine **3a** under the typical conditions of Method A. As it is seen (Scheme 3), all these substrates reacted smoothly producing the corresponding products **4aa**–**4ag** in good to high yields. The presence of isopropyl substituent at C-5 did not affect the reaction performance, as the target quinoxaline **4ai** bearing isopropyl-substituted aryl group was also obtained in high yield (Scheme 3). In most cases, the direct conversion of spiranes **5** into quinoxalines **4** (Method B) gives yields comparable to those obtained via Method A (Scheme 3). And, expectedly, reactions in the presence of non-symmetric diamine **3b** afforded mixtures of regioisomeric products **4** and **4′** with moderate to mediocre selectivity (Scheme 3).

**Figure 1:**
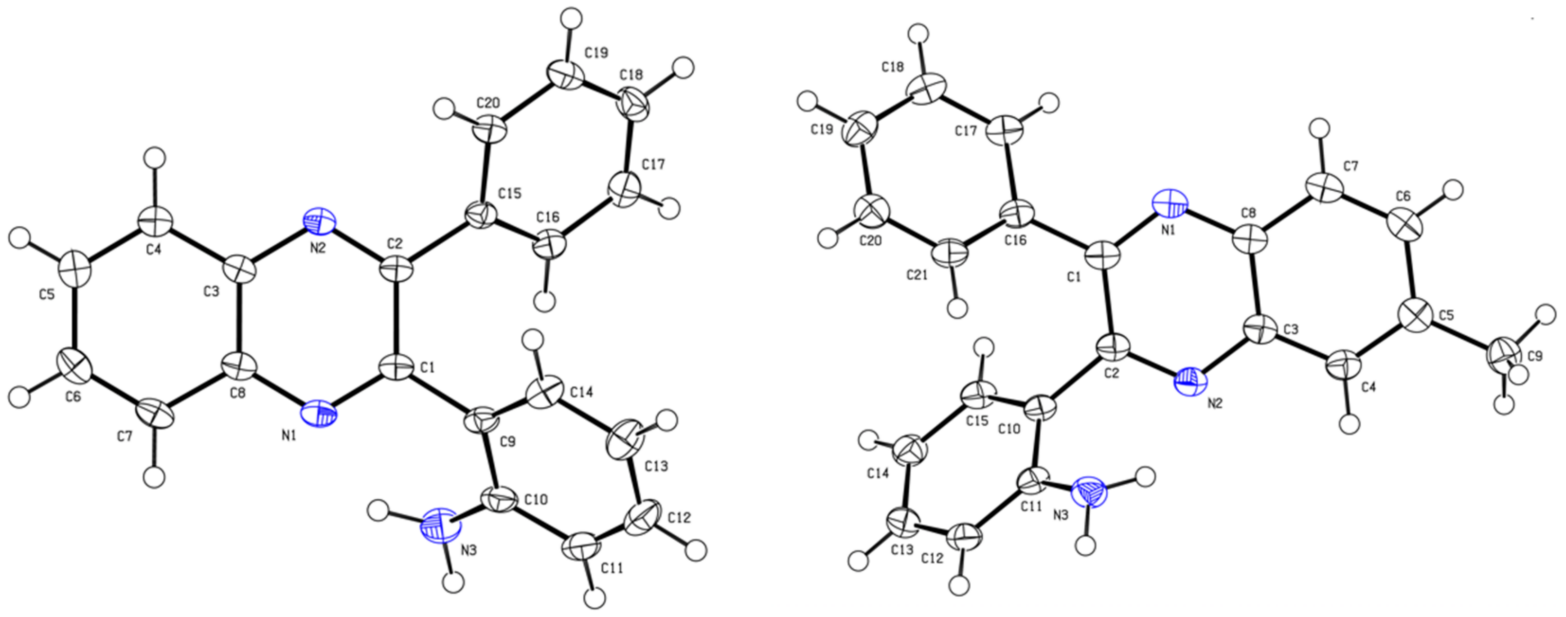
ORTEP drawings of X-ray structures for 2-(3-phenylquinoxalin-2-yl)aniline **4aa** (left, CCDC #2195374) and 2-(7-methyl-3-phenylquinoxalin-2-yl)aniline **4′bb** (right, CCDC #2195382). Thermal ellipsoids for non-hydrogen atoms are shown at 50% probability. Blue: nitrogen, gray: carbon atoms.

It should be pointed out, that formation of quinoxaline core in the featured transformation as well as configuration of one of the regiomeric products obtained in reaction with non-symmetric diamine **3b** was unambiguously confirmed by single crystal X-ray diffraction of compounds **4aa** (CCDC #2195374) and **4′bb** (CCDC #2195382), respectively (Figure 1). Interestingly, both molecules possess close contacts, corresponding to intra- and intermolecular hydrogen bonds between *ortho*-amino groups and proximal nitrogen atoms in the heterocyclic rings. Thus, the molecules in the crystals form continuous networks linked by hydrogen bonds, which certainly increase the stabilization of these crystalline forms of the products and should affect the physicochemical properties of the samples. The aryl substituents are twisted out of the plane by 27–55 degrees, which is the optimal compromise between conjugation and steric repulsion.

## 3. Methods and Materials

**General**

NMR spectra, ^1^H and ^13^C were measured in solutions of CDCl_3_ or DMSO-*d*_6_ on a Bruker AVANCE-III HD instrument (at 400.40 or 100.61 MHz, respectively). Residual solvent signals were used as internal standards, in DMSO-*d*_6_ (2.50 ppm for ^1^H, and 40.45 ppm for ^13^C nuclei) or in CDCl_3_ (7.26 ppm for ^1^H, and 77.16 ppm for ^13^C nuclei). High-resolution mass spectra were registered with a Bruker Maxis spectrometer (electrospray ionization, in MeCN solution, using HCO_2_Na–HCO_2_H for calibration). Single crystal X-ray diffraction analysis of compounds **4aa** (C_20_H_15_N_3_) and **4′ba** (C_21_H_17_N_3_) was performed on an automatic four-circle diffractometer Agilent Super Nova, Dual, Cu at zero, Atlas S2 at 100(2) K. See Supplementary Materials for NMR (Figures S1–S25), HRMS (Figures S26–S37) spectral charts and X-ray analysis data (Figures S38 and S39 and Tables S1–S14). IR spectra were measured on FT-IR spectrometer Shimadzu IR Affinity-1S equipped with an ATR sampling module. Melting points were measured with a Stuart SMP30 apparatus. MW-assisted reactions were conducted in G10 and G30 vials using an Anton Paar Monowave 300 reactor with automatic temperature control. Reaction progress and purity of isolated compounds were controlled by TLC on ALUGRAM Xtra SIL G UV 254 plates. Column chromatography was performed with Macherey Nagel Silica gel 60 (particle size: 0.063–0.2 mm). 2-Phenyl-3*H*-indol-3-one (**2**) [24], 2,4’-diaryl-4’*H*-spiro[indole- 3,5’-isoxazole] (**5a**) [21], 4’-phenyl-2-(*p*-tolyl)-4’*H*-spiro[indole-3,5’-isoxazole] (**5b**) [21], 2-(4-methoxyphenyl)-4’-phenyl-4’*H*-spiro[indole-3,5’-isoxazole] (**5c**) [2]_,_ 2-(naphthalen-2- yl)-4’-phenyl-4’*H*-spiro[indole-3,5’-isoxazole] (**5d**) [21], 4’-phenyl-2-(5,6,7,8-tetrahydronaphthalen-2-yl)-4’*H*-spiro[indole-3,5’-isoxazole] (**5e**) [21], 2-(2,3-dihydrobenzo[*b*][1,4]dioxin-6-yl)-4’-phenyl-4’*H*-spiro[indole-3,5’-isoxazole] (**5f**) [21], 2-methyl-4’-phenyl-4’*H*- spiro[indole-3,5’-isoxazole] (**5g**) [21], 4’-phenyl-2-(thiophen-2-yl)-4’*H*-spiro[indole- 3,5’-isoxazole] (**5h**) [21], 5-isopropyl-2,4’-diphenyl-4’*H*-spiro[indole-3,5’-isoxazole] (**5i**) [21], 2-(3-oxo-2-phenylindolin-2-yl)-2-phenylacetonitrile (**6a**) [21], 2-(3-oxo-2-(*p*-tolyl)indolin-2-yl)-2-phenylacetonitrile (**6b**) [21], 2-(2-(4-methoxyphenyl)-3-oxoindolin-2-yl)- 2-phenylacetonitrile (**6c**) [21], 2-(2-(naphthalen-2-yl)-3-oxoindolin-2-yl)-2-phenylacetonitrile (**6d**) [21], 2-(3-oxo-2-(5,6,7,8-tetrahydronaphthalen-2-yl)indolin-2-yl)-2-phenylacetonitrile (**6e**) [21], 2-(2-(2,3-dihydrobenzo[*b*][1,4]dioxin-6-yl)-3-oxoindolin-2-yl)-2- phenylacetonitrile (**6f**) [21], 2-(2-methyl-3-oxoindolin-2-yl)-2-phenylacetonitrile (**6g**) [21], 2-(3-oxo-2-(thiophen-2-yl)indolin-2-yl)-2-phenylacetonitrile (**6h**) [21]_,_ 2-(5-isopropyl-3- oxo-2-phenylindolin-2-yl)-2-phenylacetonitrile (**6i**) [21] were synthesized according to procedures published in our recent reports and were identical to those were described. All other reagents and solvents were purchased from commercial vendors and used as received.

**Method A for preparation of quinoxalines 4:**

2-(3-Oxo-2-phenylindolin-2-yl)-2-phenylacetonitrile (**6**) (1.00 mmol) in 2 mL of xylene and 1,2-phenylenediamine (**3**) (216 mg, 2.00 mmol) were charged in a G10 vial. The vial was sealed and heated in the microwave apparatus at 220 °C for 1 h. After completion of the reaction vial was opened and the reaction mixture concentrated in vacuo. The crude material was purified by column chromatography (EtOAc/Hexane, 1:3, *v*/*v*).

**Method B for preparation of quinoxalines 4:**

2,4’-Diaryl-4’*H*-spiro[indole-3,5’-isoxazole] (**5**) (1.00 mmol) in 2 mL of xylene and 1,2-phenylenediamine (**3**) (216 mg, 2.00 mmol) were charged in a G10 vial. The vial was sealed and heated in the microwave apparatus at 220 °C for 1 h. After completion of the reaction, the vial was opened, and the reaction mixture was concentrated in vacuo. The crude material was purified by column chromatography (EtOAc/Hexane, 1:3, *v*/*v*).

**Method C for preparation of quinoxalines 4:**

2-Phenyl-3*H*-indol-3-one (**2**) [1] (207 mg, 1.00 mmol) in 2 mL of xylene and 1,2-phenylenediamine (216 mg, 2.00 mmol) were charged in a G10 vial. The vial was sealed, and heated in the microwave apparatus at 220 °C for 15 min. After completion of the reaction, the vial was opened, and the reaction mixture was concentrated in vacuo. The crude material was purified by column chromatography (EtOAc/Hexane, 1:3, *v*/*v*).

**Method D for preparation of quinoxalines 4 (Scale-up procedure):**

2-(3-Oxo-2-phenylindolin-2-yl)-2-phenylacetonitrile (**6**) (1.62 g, 5.00 mmol) in 10 mL of xylene and 1,2-phenylenediamine (**3**) (1.08 g, 10.00 mmol) were charged in a G30 vial. The vial was sealed and heated in the microwave apparatus at 220 °C for 1 h. After completion of reaction, the vial was opened, and the reaction mixture was concentrated in vacuo. The crude material was purified by column chromatography (EtOAc/Hexane, 1:3, *v*/*v*).

*2-(3-Phenylquinoxalin-2-yl)aniline* (**4aa**): This compound was prepared via **Method A** employing 2-(3-oxo-2-phenylindolin-2-yl)-2-phenylacetonitrile (**6a**) [21] (324 mg, 1.00 mmol) and 1,2-phenylenediamine (216 mg, 2.00 mmol) in a yield of 234 mg (0.79 mmol, 79%), or via **Method B** employing 2,4’-diaryl-4’*H*-spiro[indole-3,5’-isoxazole] (**5a**) [2] (324 mg, 1.00 mmol) and 1,2-phenylenediamine (216 mg, 2.00 mmol) in a yield of 217 mg (0.73 mmol, 73%), or via **Method C** employing 2-phenyl-3*H*-indol-3-one (**2**) [1] (207 mg, 1 mmol) and 1,2-phenylenediamine (216 mg, 2.00 mmol) in a yield of 267 mg (0.9 mmol, 90%). The scale-up procedure employing **Method D** afforded the same product in a yield of 1.098 g (3.70 mmol, 74%). Purification was performed by column chromatography (EtOAc/Hexane = 1:3). The titled compound was obtained as yellow solid, m.p. 149.7–151.0 °C, R*_f_* 0.26 (EtOAc/Hexane = 1:3). ^1^H NMR (400 MHz, CDCl_3_) δ 8.23–8.14 (m, 1H), 8.14–8.06 (m, 1H), 7.83–7.72 (m, 2H), 7.64–7.53 (m, 2H), 7.40–7.28 (m, 3H), 7.17–7.08 (m, 1H), 6.89–6.77 (m, 2H), 6.55 (t, *J* = 7.5 Hz, 1H), 4.63 (br. s, 2H); ^13^C{^1^H} NMR (101 MHz, CDCl_3_) δ 154.4, 153.1, 145.5, 141.2, 140.5, 139.0, 131.9, 130.1 (3C), 129.7 (2C), 129.4, 129.0, 128.8, 128.4 (2C), 123.3, 118.2, 117.1; FTIR, *v_max_*: 3454, 3345, 3063, 1620, 1489, 1464, 1441, 1403, 1350, 1307 cm^−1^; HRMS (ESI TOF) *m*/*z*: calc’d for C_20_H_15_N_3_Na [M + Na]^+^: 320.1158, found 320.1167 (-2.9 ppm).

*2-(3-(p-Tolyl)quinoxalin-2-yl)aniline* (**4ab**): This compound was prepared via **Method A** employing 2-(3-oxo-2-(*p*-tolyl)indolin-2-yl)-2-phenylacetonitrile (**6b**) [21] (338 mg, 1.00 mmol) and 1,2-phenylenediamine (216 mg, 2.00 mmol) in a yield of 260 mg (0.84 mmol, 84%). Alternatively, this compound was prepared via **Method B** employing 4’-phenyl-2-(*p*-tolyl)-4’*H*-spiro[indole-3,5’-isoxazole] (**5b**) [21] (338 mg, 1.00 mmol) and 1,2-phenylenediamine (216 mg, 2.00 mmol) in a yield of 264 mg (0.85 mmol, 85%). Purification was performed by column chromatography (EtOAc/Hexane = 1:3). The titled compound was obtained as yellow solid, m.p. 136.3–137.9 °C, R*_f_* 0.29 (EtOAc/Hexane = 1:3). ^1^H NMR (400 MHz, CDCl_3_) δ 8.17 (d, *J* = 9.9 Hz, 1H), 8.10 (d, *J* = 7.1 Hz, 1H), 7.81–7.70 (m, 2H), 7.49 (d, *J* = 8.1 Hz, 2H), 7.13 (d, *J* = 8.1 Hz, 3H), 6.90 (d, *J* = 7.7 Hz, 1H), 6.81 (d, *J* = 8.1 Hz, 1H), 6.58 (t, *J* = 7.5 Hz, 1H), 4.59 (br. s, 2H), 2.35 (s, 3H); ^13^C{^1^H} NMR (101 MHz, CDCl_3_) δ 154.4, 153.0, 145.4, 141.2, 140.4, 139.0, 136.1, 131.8, 130.0 (2C), 129.9, 129.6 (2C), 129.3, 129.1 (2C), 128.7, 123.6, 118.2, 117.1, 21.5; FTIR, *v_max_*: 3461, 3348, 3030, 1615, 1498, 1445, 1393, 1342, 1307, 1221 cm^−1^; HRMS (ESI TOF) *m*/*z*: calc’d for C_21_H_18_N_3_ [M + H]^+^: 312.1495, found 312.1498 (−1.0 ppm).

*2-(3-(4-Methoxyphenyl)quinoxalin-2-yl)aniline* (**4ac**): This compound was prepared via **Method A** employing 2-(2-(4-methoxyphenyl)-3-oxoindolin-2-yl)-2-phenylacetonitrile (**6c**) [21] (354 mg, 1.00 mmol) and 1,2-phenylenediamine (216 mg, 2.00 mmol) in a yield of 255 mg (0.78 mmol, 78%). Alternatively, this compound was prepared via **Method B** employing 2-(4-methoxyphenyl)-4’-phenyl-4’*H*-spiro[indole-3,5’-isoxazole] (**5c**) [21] (354 mg, 1.00 mmol) and 1,2-phenylenediamine (216 mg, 2.00 mmol) in a yield of 249 mg (0.76 mmol, 76%). Purification was performed by column chromatography (Benzene/EA, 10:1, *v*/*v*). The titled compound was obtained as yellow solid, m.p. 215–217 °C; R*_f_* 0.4 (Benzene/EA, 10:1, *v*/*v*). ^1^H NMR (400 MHz, CDCl_3_) δ 8.19–8.11 (m, 1H), 8.12–8.06 (m, 1H), 7.79–7.71 (m, 2H), 7.55 (d, *J* = 8.6 Hz, 2H), 7.14 (t, *J* = 7.3 Hz, 1H), 6.91 (d, *J* = 7.5 Hz, 1H), 6.83 (dd, *J* = 12.8, 8.4 Hz, 3H), 6.60 (t, *J* = 7.5 Hz, 1H), 4.56 (s, 2H), 3.81 (s, 3H); ^13^C{^1^H} NMR (101 MHz, CDCl_3_) δ 160.3, 153.9, 152.9, 145.3, 141.2, 140.3, 131.7, 131.3, 131.2 (2C), 130.1, 130.0, 129.8, 129.2, 128.7, 123.6, 118.3, 117.1, 113.8 (2C), 55.4; FTIR, *v_max_*: 3451, 3241, 1679, 1611, 1556, 1537, 1456, 1418, 1271, 1231 cm^−1^; HRMS (ESI TOF) m/z: calc’d for C_21_H_18_N_3_O [M + H]^+^: 328.1444, found 328.1439 (1.7 ppm).

*2-(3-(Naphthalen-2-yl)quinoxalin-2-yl)aniline* (**4ad**): This compound was prepared via **Method A** employing 2-(2-(naphthalen-2-yl)-3-oxoindolin-2-yl)-2-phenylacetonitrile (**6d**) [21] (374 mg, 1.00 mmol) and 1,2-phenylenediamine (216 mg, 2.00 mmol) in a yield of 284 mg (0.82 mmol, 82%). Alternatively, this compound was prepared via **Method B** employing 2-(naphthalen-2-yl)-4’-phenyl-4’*H*-spiro[indole-3,5’-isoxazole] (**5d**) [21] (374 mg, 1.00 mmol) and 1,2-phenylenediamine (216 mg, 2.00 mmol) in a yield of 271 mg (0.78 mmol, 78%). Purification was performed by column chromatography (EtOAc/Hexane = 1:3). The titled compound was obtained as yellow solid, m.p. 181.0–182.0 °C, R*_f_* 0.23 (EtOAc/Hexane = 1:3). ^1^H NMR (400 MHz, CDCl_3_) δ 8.27–8.17 (m, 2H), 8.17–8.09 (m, 1H), 7.86–7.77 (m, 4H), 7.75 (d, *J* = 8.6 Hz, 1H), 7.60 (d, *J* = 8.6 Hz, 1H), 7.54–7.43 (m, 2H), 7.17–7.07 (m, 1H), 6.89 (d, *J* = 7.8 Hz, 1H), 6.82 (d, *J* = 7.0 Hz, 1H), 6.49 (t, *J* = 6.9 Hz, 1H), 4.67 (br. S, 2H); ^13^C{^1^H} NMR (101 MHz, CDCl_3_) δ 154.2, 153.2, 145.5, 141.3, 140.6, 136.5, 133.4, 133.3, 131.9, 130.2 (3C), 129.6, 129.4, 128.9, 128.8, 127.8 (2C), 126.9 (2C), 126.3, 123.3, 118.3, 117.1; FTIR, *v_max_*: 3331, 1773, 1635, 1562, 1509, 1478, 1456, 1398, 1342, 1305, 1242 cm^−1^; HRMS (ESI TOF) m/z: calc’d for C_24_H_18_N_3_ [M + H]^+^: 348.1495, found 348.1501 (−1.6 ppm).

*2-(3-(5,6,7,8-Tetrahydronaphthalen-2-yl)uinoxaline-2-yl)aniline* (**4ae**): This compound was prepared via **Method A** employing 2-(3-oxo-2-(5,6,7,8-tetrahydronaphthalen- 2-yl)indolin-2-yl)-2-phenylacetonitrile (**6e**) [21] (378 mg, 1.00 mmol) and 1,2-phenylenediamine (216 mg, 2.00 mmol) in a yield of 302 mg (0.86 mmol, 86%). Alternatively, this compound was prepared via **Method B** employing 4′-phenyl-2-(5,6,7,8-tetrahydronaphthalen-2-yl)-4′*H*-spiro[indole-3,5′-isoxazole] (**5e**) [21] (378 mg, 1.00 mmol) and 1,2-phenylenediamine (216 mg, 2.00 mmol) in a yield of 295 mg (0.84 mmol, 84%). Purification was performed by column chromatography (EtOAc/Hexane = 1:3). The titled compound was obtained as a brown oil, R*_f_* 0.31 (EtOAc/Hexane = 1:3). ^1^H NMR (400 MHz, CDCl_3_) δ 8.21–8.13 (m, 1H), 8.13–8.05 (m, 1H), 7.81–7.69 (m, 2H), 7.40 (s, 1H), 7.21–7.10 (m, 2H), 6.98–6.90 (m, 2H), 6.81 (d, *J* = 6.8 Hz, 1H), 6.60 (t, *J* = 6.9 Hz, 1H), 4.58 (br. s, 2H), 2.74 (d, *J* = 3.3 Hz, 4H), 1.78 (quint, *J* = 3.5 Hz, 4H); ^13^C{^1^H} NMR (101 MHz, CDCl_3_) δ 154.5, 153.1, 145.3, 141.3, 140.4, 138.4, 137.3, 136.0, 131.8, 130.3, 130.0 (2C), 129.8, 129.3, 128.9, 128.7, 126.8, 123.7, 118.2, 117.0, 29.5, 29.4, 23.2 (2C); FTIR, *v_max_*: 3457, 3358, 3060, 2232, 1617, 1498, 1448, 1343, 1307, 1249 cm^−1^; HRMS (ESI TOF) *m*/*z*: calc’d for C_24_H_22_N_3_ [M + H]^+^: 352.1808, found 352.1817 (−2.5 ppm).

*2-(3-(2,3-Dihydrobenzo[b]*[1,4]*dioxin-6-yl)quinoxalin-2-yl)aniline* (**4af**): This compound was prepared via **Method A** employing 2-(2-(2,3-dihydrobenzo[*b*][1,4]dioxin-6-yl)- 3-oxoindolin-2-yl)-2-phenylacetonitrile (**6f**) [21] (382 mg, 1.00 mmol) and 1,2-phenylenediamine (216 mg, 2.00 mmol) in a yield of 308 mg (0.87 mmol, 87%). Alternatively, this compound was prepared via **Method B** employing 2-(2,3-dihydrobenzo[*b*][1,4]dioxin-6-yl)-4’-phenyl-4’*H*-spiro[indole-3,5’-isoxazole] (**5f**) [21] (382 mg, 1.00 mmol) and 1,2-phenylenediamine (216 mg, 2.00 mmol) in a yield of 298 mg (0.84 mmol, 84%). Purification was performed by column chromatography (EtOAc/Hexane = 1:2). The titled compound was obtained as brown oil, R*_f_* 0.31 (EtOAc/Hexane = 1:2). ^1^H NMR (400 MHz, DMSO-*d_6_*) δ 8.14–8.04 (m, 2H), 7.88–7.78 (m, 2H), 7.13–7.04 (m, 2H), 7.01 (d, *J* = 10.8 Hz, 1H), 6.87 (d, *J* = 7.5 Hz, 1H), 6.79–6.70 (m, 2H), 6.49 (t, *J* = 7.5 Hz, 1H), 5.20 (br. s, 2H), 4.22 (d, *J* = 6.6 Hz, 4H); ^13^C{^1^H} NMR (101 MHz, DMSO-*d_6_*) δ 152.9 (2C), 146.3, 144.2, 142.8, 140.6, 140.2, 131.8, 130.5, 130.1, 129.8, 129.5, 128.8, 128.7, 123.2, 122.6, 118.2, 116.5, 115.7, 115.5, 64.3, 64.0; FTIR, *v_max_*: 3447, 3338, 3235, 1634, 1622, 1587, 1509, 1496, 1456, 1418, 1396, 1343, 1299, 1279, 1259 cm^−1^; HRMS (ESI TOF) m/z: calc’d for C_22_H_18_N_3_O_2_ [M + H]^+^: 356.1394, found 356.1387 (1.9 ppm).

*2-(3-Methylquinoxalin-2-yl)aniline* (**4ag**): This compound was prepared via **Method A** employing 2-(2-methyl-3-oxoindolin-2-yl)-2-phenylacetonitrile (**6g**) [21] (262 mg, 1.00 mmol) and 1,2-phenylenediamine (216 mg, 2.00 mmol) in a yield of 170 mg (0.72 mmol, 72%). Alternatively, this compound was prepared via **Method B** employing 2-methyl-4’-phenyl-4’*H*-spiro[indole-3,5’-isoxazole] (**5g**) [21] (262 mg, 1.00 mmol) and 1,2-phenylenediamine (216 mg, 2.00 mmol) in a yield of 176 mg (0.75 mmol, 75%). Purification was performed by column chromatography (EtOAc/Hexane = 1:1). The titled compound was obtained as a yellow oil, R*_f_* 0.43 (EtOAc/Hexane = 1:1). ^1^H NMR (400 MHz, DMSO-*d_6_*) δ 8.04 (d, *J* = 1.9 Hz, 2H), 7.85–7.73 (m, 2H), 7.17 (t, *J* = 7.6 Hz, 1H), 7.11 (d, *J* = 7.6 Hz, 1H), 6.81 (d, *J* = 8.1 Hz, 1H), 6.67 (t, *J* = 7.4 Hz, 1H), 5.11 (br. s, 2H), 2.57 (s, 3H); ^13^C{^1^H} NMR (101 MHz, DMSO-*d_6_*) δ 154.5, 153.9, 146.2, 140.8, 140.6, 129.8, 129.6 (2C), 129.0, 128.8, 128.1, 123.0, 115.9, 115.4, 22.9; FTIR, *v_max_*: 3437, 3312, 3189, 1642, 1602, 1569, 1499, 1486, 1456, 1393, 1372, 1340, 1307 cm^−1^; HRMS (ESI TOF) *m*/*z*: calc’d for C_15_H_14_N_3_ [M + H]^+^: 236.1182, found 236.1187 (−2.2 ppm).

*2-(3-(Thiophen-2-yl)quinoxalin-2-yl)aniline* (**4ah**): This compound was prepared via **Method A** employing 2-(3-oxo-2-(thiophen-2-yl)indolin-2-yl)-2-phenylacetonitrile (**6h**) [21] (330 mg, 1.00 mmol) and 1,2-phenylenediamine (216 mg, 2.00 mmol) in a yield of 212 mg (0.70 mmol, 70%). Alternatively, this compound was prepared via **Method B** employing 4’-phenyl-2-(thiophen-2-yl)-4’*H*-spiro[indole-3,5’-isoxazole] (**5h**) [21] (330 mg, 1.00 mmol) and 1,2-phenylenediamine (216 mg, 2.00 mmol) in a yield of 203 mg (0.67 mmol, 67%). Purification was performed by column chromatography (Benzene). The titled compound was obtained as green-yellow solid, m.p. 131–132 °C; R*_f_* 0.3 (Benzene). ^1^H NMR (400 MHz, CDCl_3_) δ 8.14–8.10 (m, 1H), 8.10–8.04 (m, 1H), 7.80–7.74 (m, 1H), 7.74–7.69 (m, 1H), 7.41 (dd, *J* = 5.0, 1.1 Hz, 1H), 7.33–7.27 (m, 1H), 7.20 (dd, *J* = 7.6, 1.4 Hz, 1H), 6.96 (dd, *J* = 3.8, 1.1 Hz, 1H), 6.91 (dd, *J* = 5.0, 3.8 Hz, 1H), 6.89–6.81 (m, 2H), 4.03 (br. s, 2H); ^13^C{^1^H} NMR (101 MHz, CDCl_3_) δ 151.1, 147.7, 144.8, 142.6, 141.3, 140.5, 130.7, 130.7, 130.5, 129.8, 129.8, 129.0 (2C), 128.9, 128.2, 124.2, 119.0, 117.0; FTIR, *v_max_*: 3331, 3063, 1779, 1610, 1471, 1435, 1305, 1242 cm^−1^; HRMS (ESI TOF) m/z: calc’d for C_18_H_14_N_3_S [M + H]^+^: 304.0903, found 304.0904 (−0.3 ppm).

*4-Isopropyl-2-(3-phenylquinoxalin-2-yl)aniline* (**4ai**): This compound was prepared via **Method A** employing 2-(5-isopropyl-3-oxo-2-phenylindolin-2-yl)-2-phenylacetonitrile (**6i**) [21] (366 mg, 1.00 mmol) and 1,2-phenylenediamine (216 mg, 2.00 mmol) in a yield of 281 mg (0.83 mmol, 83%). Alternatively, this compound was prepared via **Method B** employing 5-isopropyl-2,4’-diphenyl-4’*H*-spiro[indole-3,5’-isoxazole] (**5i**) [21] (366 mg, 1.00 mmol) and 1,2-phenylenediamine (216 mg, 2.00 mmol) in a yield of 275 mg (0.81 mmol, 81%). Purification was performed by column chromatography (EtOAc/Hexane = 1:2). The titled compound was obtained as yellow oil, R*_f_* 0.47 (EtOAc/Hexane = 1:2). ^1^H NMR (400 MHz, CDCl_3_) δ 1H NMR (400 MHz, CDCl_3_) δ 8.22–8.14 (m, 1H), 8.14–8.06 (m, 1H), 7.81–7.71 (m, 2H), 7.55 (s, 2H), 7.33 (s, 3H), 6.96 (d, *J* = 8.2 Hz, 1H), 6.75 (d, *J* = 8.3 Hz, 1H), 6.67 (s, 1H), 4.71 (br. s, 2H), 2.61–2.46 (m, 1H), 0.87 (d, *J* = 7.0 Hz, 6H); ^13^C{^1^H} NMR (101 MHz, CDCl_3_) δ 154.7, 153.5, 143.5, 141.0, 140.4, 139.4, 138.3, 130.1, 130.0 (2C), 129.7 (2C), 129.3, 128.7 (2C), 128.4, 128.3 (2C), 122.4, 117.1, 33.0, 23.9 (2C); FTIR, *v_max_*: 3444, 3348, 3053, 2954, 2868, 1897, 1624, 1501, 1425, 1400, 1343, 1290, 1221, 1194 cm^−1^; HRMS (ESI TOF) m/z: calc’d for C_23_H_22_N_3_ [M + H]^+^: 340.1808, found 340.1816 (−2.2 ppm).

*2-(6-Methyl-3-phenylquinoxalin-2-yl)aniline* (**4ba**) *and 2-(7-Methyl-3-phenylquinoxalin-2-yl)aniline* (**4′ba**): This compounds was prepared via **Method A** employing 2-(3-oxo-2-phenylindolin-2-yl)-2-phenylacetonitrile (**6a**) [21] (324 mg, 1.00 mmol) and 4-methyl-1,2-phenylenediamine (244 mg, 2.00 mmol) in a yield of 300 mg (0.96 mmol, **4ba/4′ba** = 4.65:1.0, total yield 96%). Purification was performed by column chromatography (EtOAc/Hexane = 1:3). The titled compounds were obtained as yellow solid, m.p. 156.3–159.2 °C, R*_f_* 0.29 (EtOAc/Hexane = 1:3). Major isomer (**4ba**): ^1^H NMR (400 MHz, CDCl_3_) δ 8.06 (d, *J* = 8.6 Hz, 1H), 7.88 (s, 1H), 7.65–7.53 (m, 3H), 7.33 (s, 3H), 7.12 (t, *J* = 7.7 Hz, 1H), 6.88–6.77 (m, 2H), 6.55 (t, *J* = 7.5 Hz, 1H), 4.61 (br. s, 2H), 2.62 (s, 3H); ^13^C{^1^H} NMR (101 MHz, CDCl_3_) δ 153.5, 152.9, 145.5, 140.6, 139.7, 139.2, 132.5, 132.4, 131.9, 130.0, 129.7 (2C), 128.9, 128.8, 128.3 (2C), 127.6, 123.4, 118.1, 117.0, 22.1; Minor isomer (**4′ba**): ^1^H NMR (400 MHz, CDCl_3_) δ 7.99 (d, *J* = 8.6 Hz, 1H), 7.95 (s, 1H), 7.65–7.53 (m, 3H), 7.33 (s, 3H), 7.12 (t, *J* = 7.7 Hz, 1H), 6.88–6.77 (m, 2H), 6.55 (t, *J* = 7.5 Hz, 1H), 4.61 (br. s, 2H), 2.62 (s, 3H); ^13^C{^1^H} NMR (101 MHz, CDCl_3_) δ 154.3, 152.1, 145.4, 141.3, 140.7 (2C), 139.0, 132.4, 131.9, 129.9, 129.7 (2C), 129.4, 128.5, 128.3 (2C), 128.2, 123.4, 118.2, 117.0, 22.1; FTIR, *v_max_*: 3421, 3335, 2921, 1632, 1557, 1484, 1446, 1348, 1300, 1250 cm^−1^; HRMS (ESI TOF) *m*/*z*: calc’d for C_21_H_18_N_3_ [M + H]^+^: 312.1495, found 312.1495 (0.0 ppm).

*2-(6-Methyl-3-(p-tolyl)quinoxalin-2-yl)aniline***(4bb)***and 2-(7-Methyl-3-(p-tolyl)quinoxalin-2-yl)aniline* (**4′bb**)**:** This compounds were prepared via **Method A** employing 2-(3-oxo-2-(*p*-tolyl)indolin-2-yl)-2-phenylacetonitrile (**6b**) [21] (338 mg, 1.00 mmol) and 4-methyl-1,2-phenylenediamine (244 mg, 2.00 mmol) in a yield of 296 mg (0.91 mmol, **4bb’:4bb** = 1.0:1.75, total yield 91%). Alternatively, these compounds were prepared via **Method B** employing 4’-phenyl-2-(*p*-tolyl)-*4’H*-spiro[indole-3,5’-isoxazole] (**5b**) [21] (338 mg, 1.00 mmol) and 5-methyl-1,2-phenylenediamine (244 mg, 2.00 mmol) in a yield of 289 mg (0.89 mmol, 89%). Purification was performed by column chromatography (EtOAc/Hexane = 1:3). The titled compounds were obtained as yellow solid, m.p. 176.1–179.6 °C, R*_f_* 0.31 (EtOAc/Hexane = 1:3). Major isomer (**4′bb**): ^1^H NMR (400 MHz, CDCl_3_) δ 7.98 (d, *J* = 8.6 Hz, 1H), 7.94 (s, 1H), 7.63–7.54 (m, 1H), 7.51–7.43 (m, 2H), 7.17–7.08 (m, 3H), 6.88 (d, *J* = 7.7 Hz, 1H), 6.80 (d, *J* = 6.8 Hz, 1H), 6.58 (t, *J* = 7.5 Hz, 1H), 4.56 (br. s, 2H), 2.61 (s, 3H), 2.34 (s, 3H); ^13^C{^1^H} NMR (101 MHz, CDCl_3_) δ 154.2, 152.0, 145.3, 141.3, 140.6, 140.4, 138.9, 136.2, 132.2, 131.8, 129.9, 129.6 (2C), 129.1 (2C), 128.3, 128.2, 123.8, 118.2, 117.0, 22.0, 21.5; Minor isomer (**4bb**): ^1^H NMR (400 MHz, CDCl_3_) δ 8.05 (d, *J* = 8.6 Hz, 1H), 7.86 (s, 1H), 7.63–7.54 (m, 1H), 7.51–7.43 (m, 2H), 7.17–7.08 (m, 3H), 6.88 (d, *J* = 7.7 Hz, 1H), 6.80 (d, *J* = 6.8 Hz, 1H), 6.58 (t, *J* = 7.5 Hz, 1H), 4.56 (br. s, 2H), 2.61 (s, 3H), 2.34 (s, 3H); ^13^C{^1^H} NMR (101 MHz, CDCl_3_) δ 153.5, 152.9, 145.4, 140.6, 140.5, 139.7, 138.8, 136.2, 132.4, 131.8, 129.9, 129.6 (2C), 129.1 (2C), 128.9, 127.6, 123.7, 118.2, 117.0, 22.0, 21.5; FTIR, *v_max_*: 3414, 3318, 3027, 1922, 1617, 1491, 1448, 1342, 1307, 1247 cm^−1^; HRMS (ESI TOF) m/z: calc’d for C_22_H_19_N_3_Na [M + Na]^+^: 348.1471, found 348.1480 (−2.7 ppm).

*2-(7-Methyl-3-(naphthalen-2-yl)quinoxalin-2-yl)aniline* (**4′bc**) *and 2-(6-Methyl-3- (naphthalen-2-yl)quinoxalin-2-yl)aniline* (**4bc**)**:** This compounds was prepared via **Method A** employing 2-(2-(naphthalen-2-yl)-3-oxoindolin-2-yl)-2-phenylacetonitrile (**6c**) [2] (374 mg, 1.00 mmol) and 4-methyl-1,2-phenylenediamine (244 mg, 2.00 mmol) in a yield of 320 mg (0.89 mmol, **4bc/4′bc** = 1.23:1.0, total yield 89%). Purification was performed by column chromatography (EtOAc/Hexane = 1:3). The titled compounds were obtained as yellow solid, m.p. 155.9–158.7 °C, R*_f_* 0.26 (EtOAc/Hexane = 1:3). Major isomer (**4bc**): ^1^H NMR (400 MHz, CDCl_3_) δ 8.19 (s, 1H), 8.10 (d, *J* = 8.4 Hz, 1H), 7.90 (s, 1H), 7.85–7.76 (m, 2H), 7.73 (d, *J* = 8.7 Hz, 1H), 7.67–7.54 (m, 2H), 7.54–7.42 (m, 2H), 7.11 (t, *J* = 7.7 Hz, 1H), 6.92–6.77 (m, 2H), 6.53–6.44 (m, 1H), 4.66 (br. s, 2H), 2.66–2.60 (m, 3H); ^13^C{^1^H} NMR (101 MHz, CDCl_3_) δ 153.3, 153.1, 145.5, 140.7, 140.7, 139.8, 136.7, 133.4 (2C), 132.6, 131.9, 130.1, 129.5, 128.9, 128.8, 127.7 (3C), 127.0, 126.8, 126.3, 123.5, 118.3, 117.1, 22.1; Minor isomer (**4′bc**): ^1^H NMR (400 MHz, CDCl_3_) δ 8.19 (s, 1H), 8.02 (d, *J* = 8.6 Hz, 1H), 7.99 (s, 1H), 7.85–7.76 (m, 2H), 7.73 (d, *J* = 8.7 Hz, 1H), 7.67–7.54 (m, 2H), 7.54–7.42 (m, 2H), 7.11 (t, *J* = 7.7 Hz, 1H), 6.92–6.77 (m, 2H), 6.53–6.44 (m, 1H), 4.66 (br. s, 2H), 2.66–2.60 (m, 3H); ^13^C{^1^H} NMR (101 MHz, CDCl_3_) δ 154.0, 152.2, 145.4, 141.4, 140.8, 139.1, 136.7, 133.4 (2C), 132.5, 131.9, 130.0, 129.6, 128.8, 128.3, 128.2, 127.7 (2C), 127.0, 126.9, 126.3, 123.5, 118.3, 117.1, 22.1; FTIR, *v_max_*: 3451, 3378, 1619, 1557, 1489, 1458, 1338, 1304, 1247 cm^−1^; HRMS (ESI TOF) *m*/*z*: calc’d for C_25_H_20_N_3_ [M + H]^+^: 362.1652, found 362.1662 (−2.8 ppm).

## 4. Conclusions

New preparative method for synthesis of diverse quinoxalines **4** based on microwave-assisted redox-neutral cascade reaction of 2-(3-oxoindolin-2-yl)-2-phenylacetonitriles **6** with benzene-1,2-diamines **3** was developed; this approach cleverly employs the unusual ability of shelf-stable molecules **6** to lose a benzyl cyanide moiety, thus acting as a synthetic precursor of very unstable 2-aryl-indol-3-ones, which are quite difficult to handle. Alternatively, the same transformation could also be carried out from 4’-phenyl-4’*H*-spiro[indole-3,5’-isoxazoles] **5**. Considering that spiranes **5** could be obtained in a single step from commonly available indoles and nitroolefins, the overall sequence provides a very convenient and affordable method for the preparation of quinoxalines. Related reactions of precursors **5** and **6** with aliphatic diamines are currently underway in our laboratories.

## Data Availability

File SI.pdf containing ^1^H and ^13^C NMR spectral charts for all newly synthesized compounds (Figures S1–S25), HRMS spectral charts (Figures S26–S37), X-Ray crystallography data (Figures S38 and S39 and Tables S1–S14).

## Supplementary Materials

The supporting information can be downloaded at: https://www.mdpi.com/article/10.3390/ijms231911120/s1. References [25,26,27] are cited in the supplementary materials.
