# Supplementary material for: A Convenient Way to Quinoxaline Derivatives through the Reaction of 2-(3-Oxoindolin-2-yl)-2-phenylacetonitriles with Benzene-1,2-diamines
Source: Int J Mol Sci. 2022 Sep 22;23(19):11120. doi: 10.3390/ijms231911120 (PMC9570350; doi:10.3390/ijms231911120)
Supplement: Supplementary file 1 [file ijms-23-11120-s001.zip › ijms-1904927-supplementary.pdf]

Electronic Supplementary Material (ESI)

Formation of quinoxalines in reaction of 2-(3-oxoindolin-2-yl)-2-phenylacetonitriles with ben-zene-1,2-diamines

Alexander V. Aksenov[1,*], Nikolai A. Arutiunov[1], Dmitrii A. Aksenov[1], Artem V. Samovolov[1], Igor A. Kurenkov[1], Nicolai A. Aksenov[1], Elena A. Aleksandrova[1], Daria S. Momotova, and Michael Rubin[1,2,*]

[1] Department of Chemistry, North Caucasus Federal University, 1a Pushkin St., 355017 Stavropol, Russia; naarutiunov@ncfu.ru

[2] Department of Chemistry, University of Kansas, 1567 Irving Hill Road, Lawrence, KS 66045, USA

* Correspondence: aaksenov@ncfu.ru (A.V.A.); mrubin@ku.edu (M.R.)

Supporting Information

NMR Spectral Charts.. S2
^{1}H and ^{13}C NMR spectral charts for 2-phenyl-3*H*-indol-3-one (2)........................ S2
^{1}H and ^{13}C NMR spectral charts for quinoxalines (4)........................ S3
HRMS spectral charts S27
HRMS spectral charts for quinoxalines (4).. S27
X-Ray crystallography data.. S33
X-Ray crystallography data for 4aa.. S33
X-Ray crystallography data for 4’ba.. S41
References.. S49

^{1}H and ^{13}C NMR spectral charts for 2-phenyl-3*H*-indol-3-one (**2**)

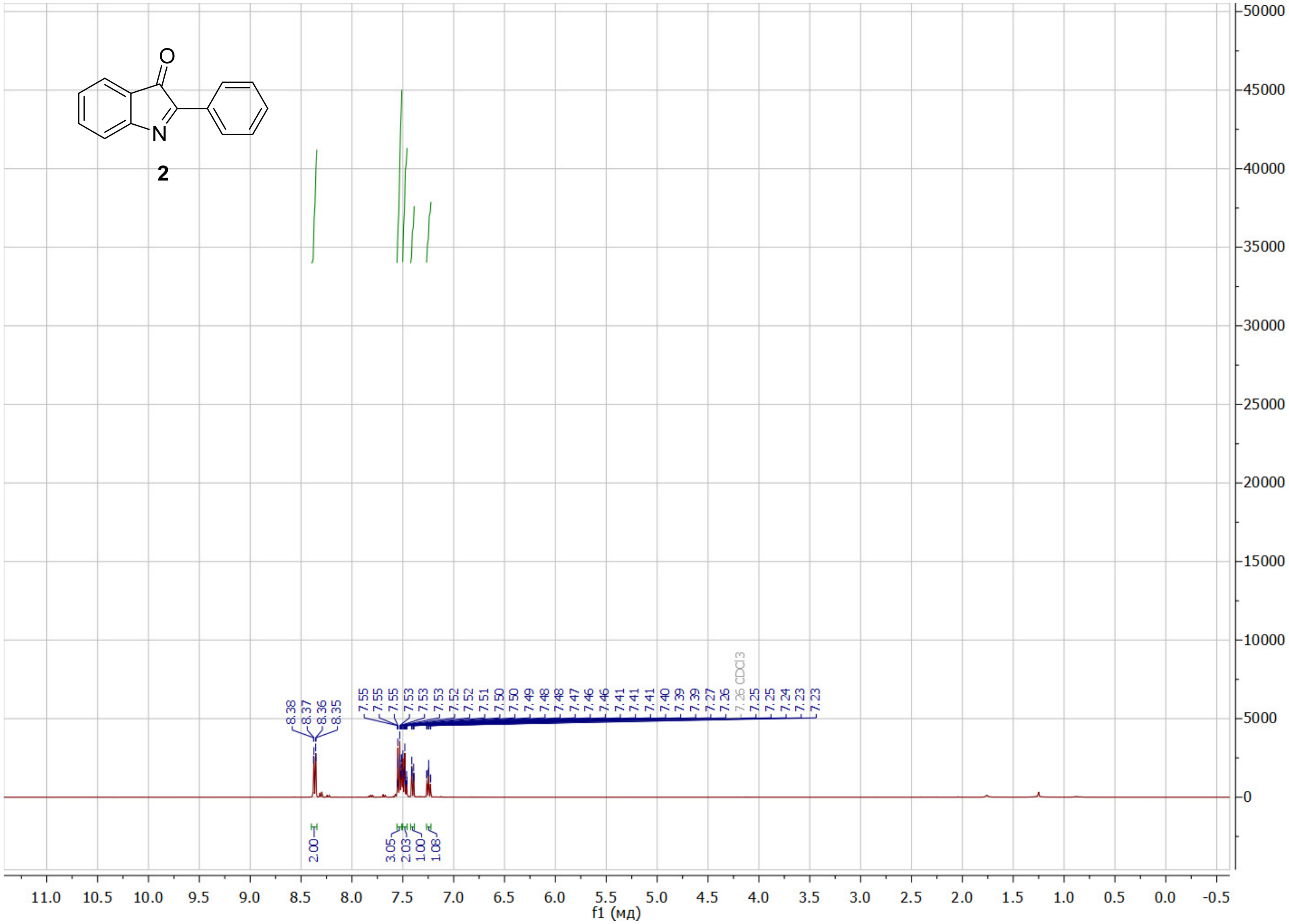

Figure S1. ^{1}H NMR spectrum of **2** in $CDCl_3$ (400 MHz)

^{1}H and ^{13}C NMR spectral charts for quinoxalines (4)

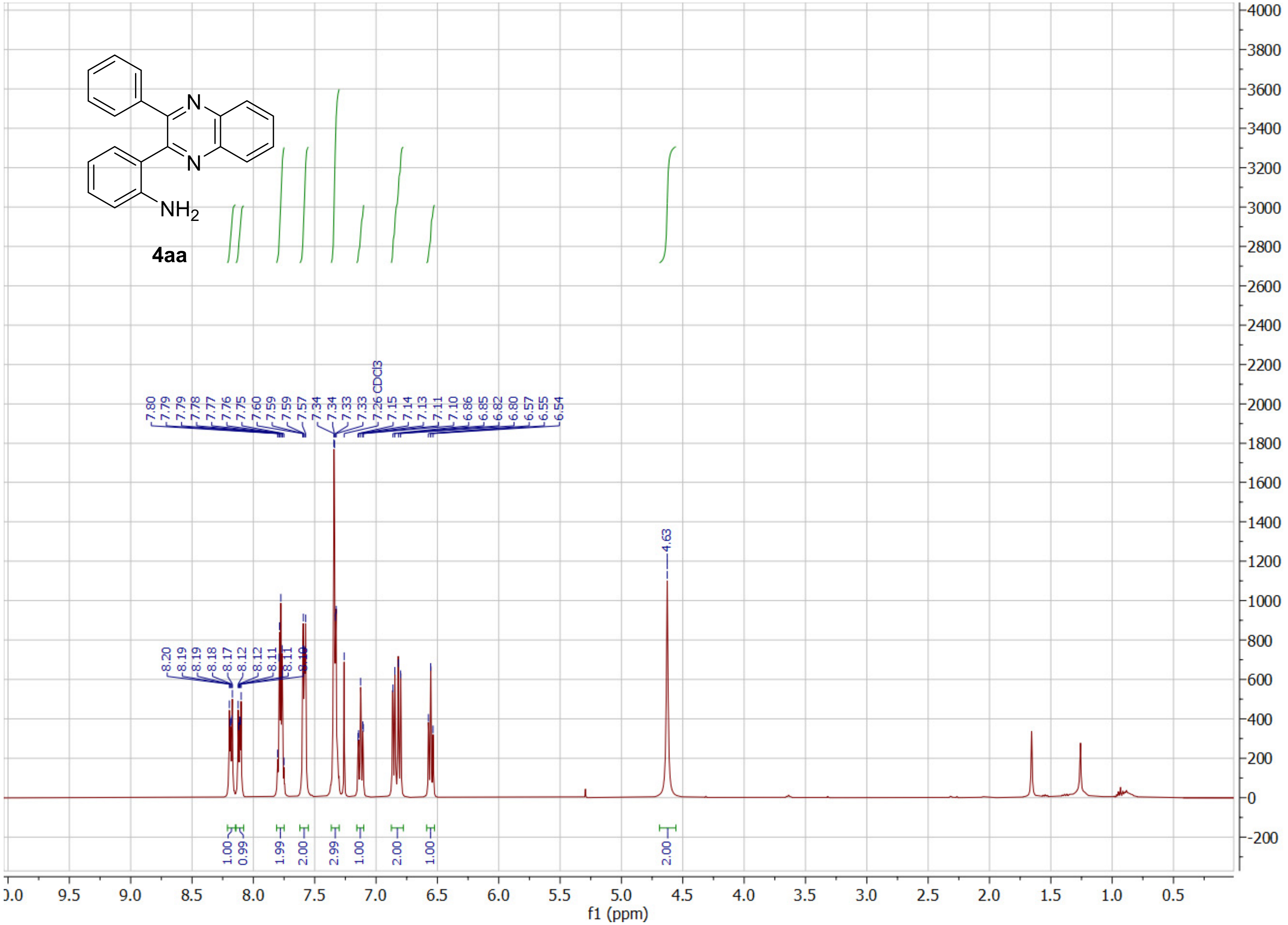

Figure S2. ^{1}H NMR spectrum of **4aa** in $CDCl_3$ (400 MHz)

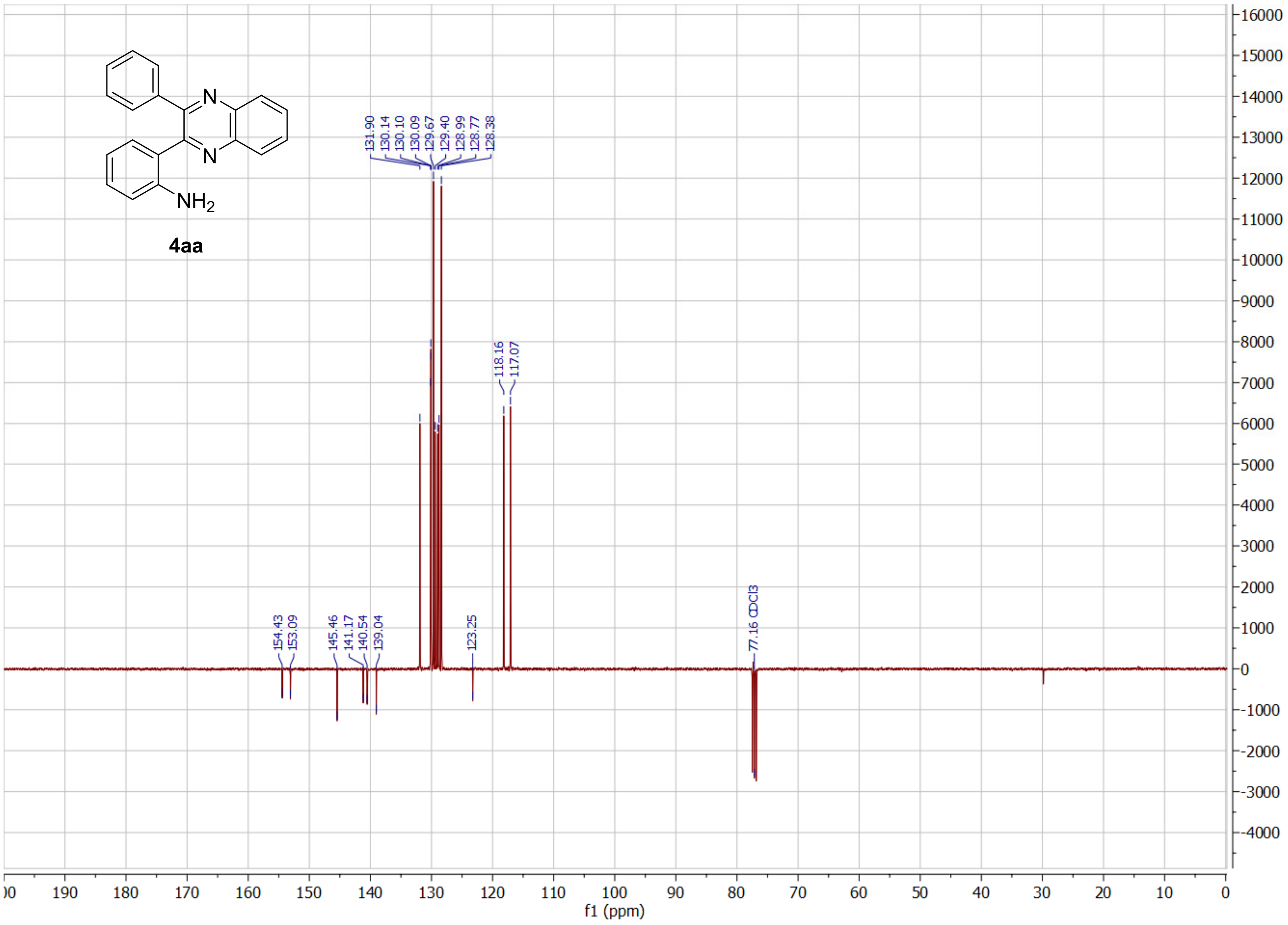

Figure S3. ^{13}C DEPTQ NMR spectrum of **4aa** in $CDCl_3$ (101 MHz)

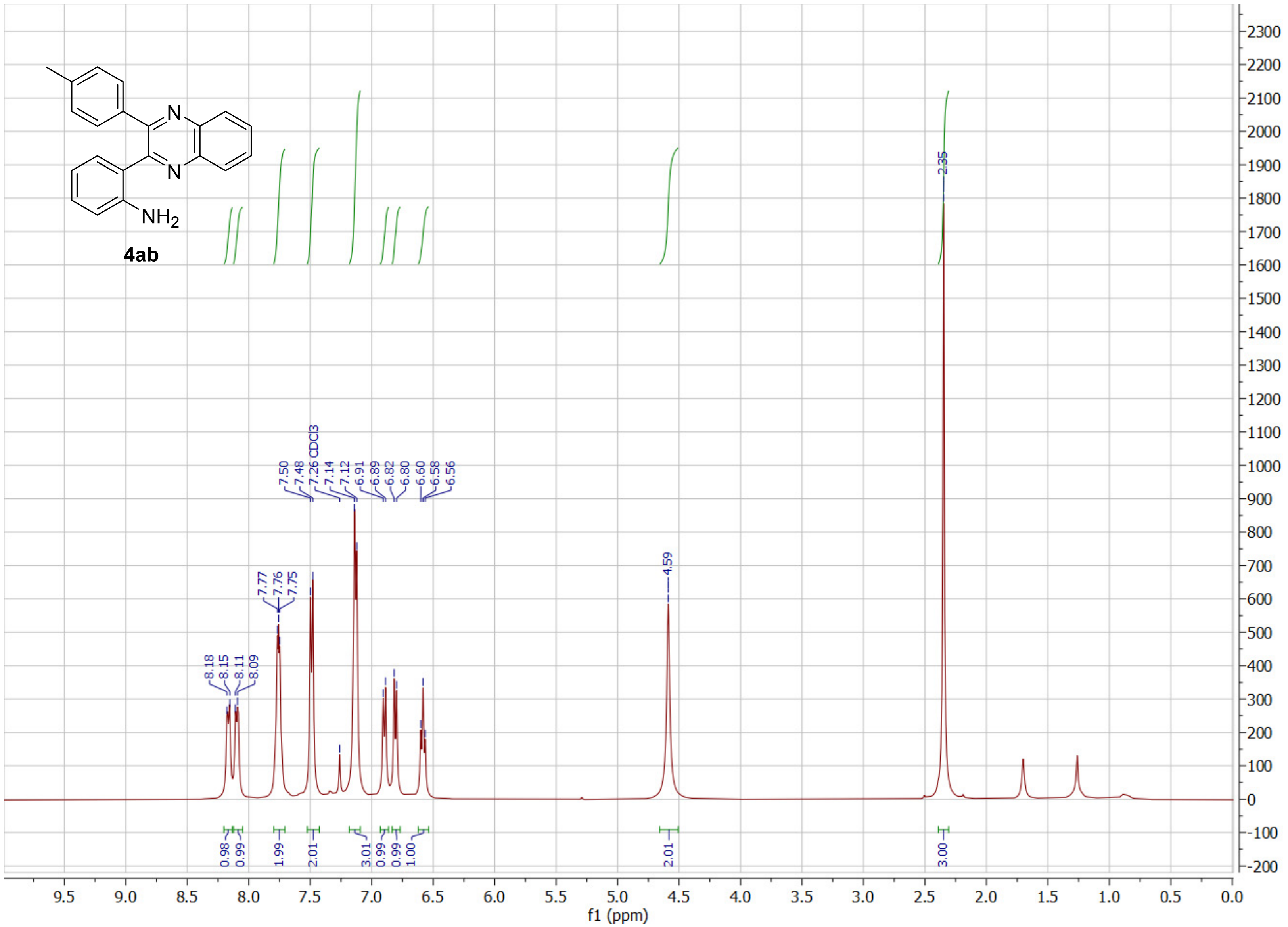

Figure S4. ^{1}H NMR spectrum of **4ab** in $CDCl_3$ (400 MHz)

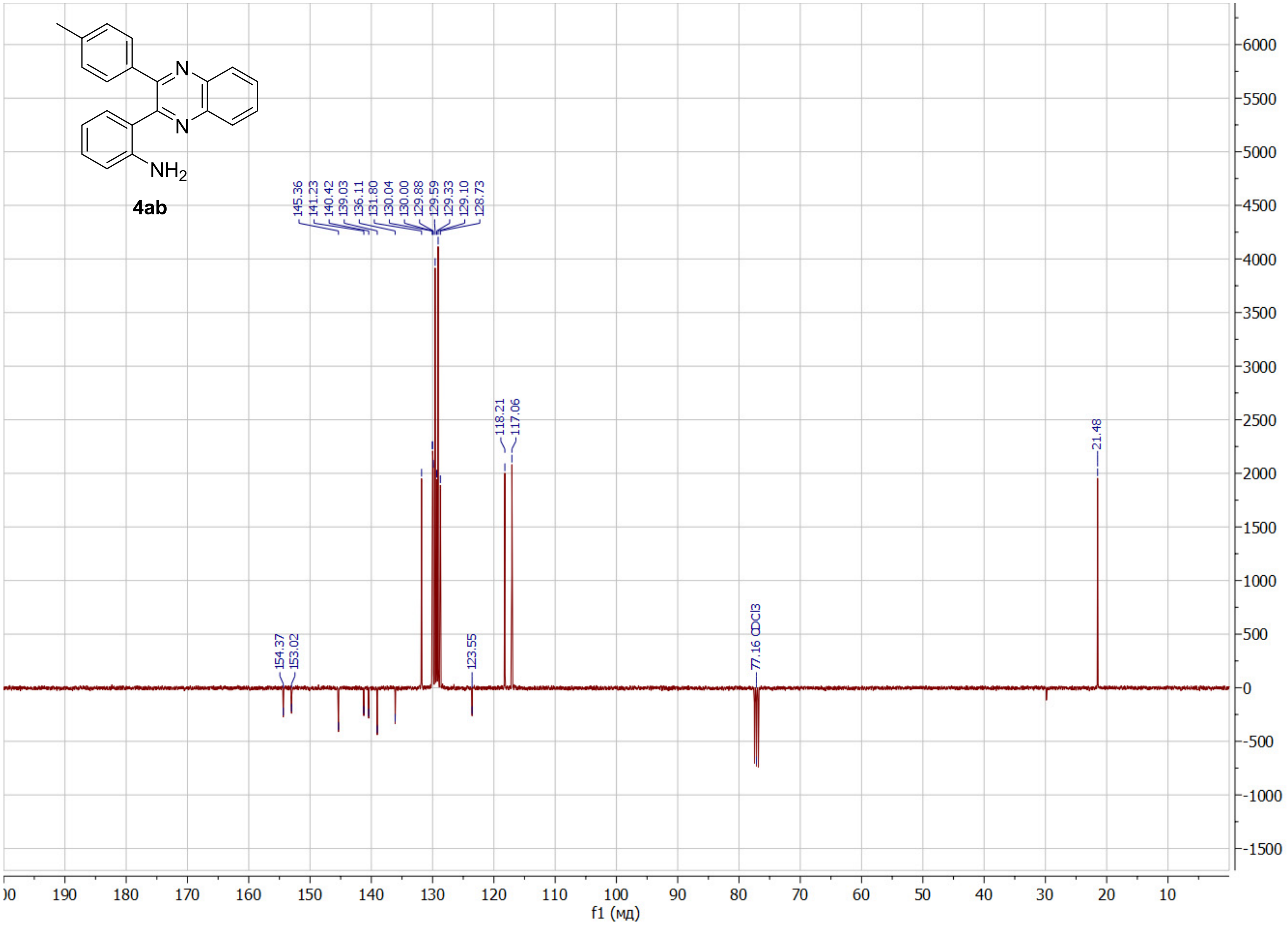

Figure S5. ^{13}C DEPTQ NMR spectrum of **4ab** in $CDCl_3$ (101 MHz)

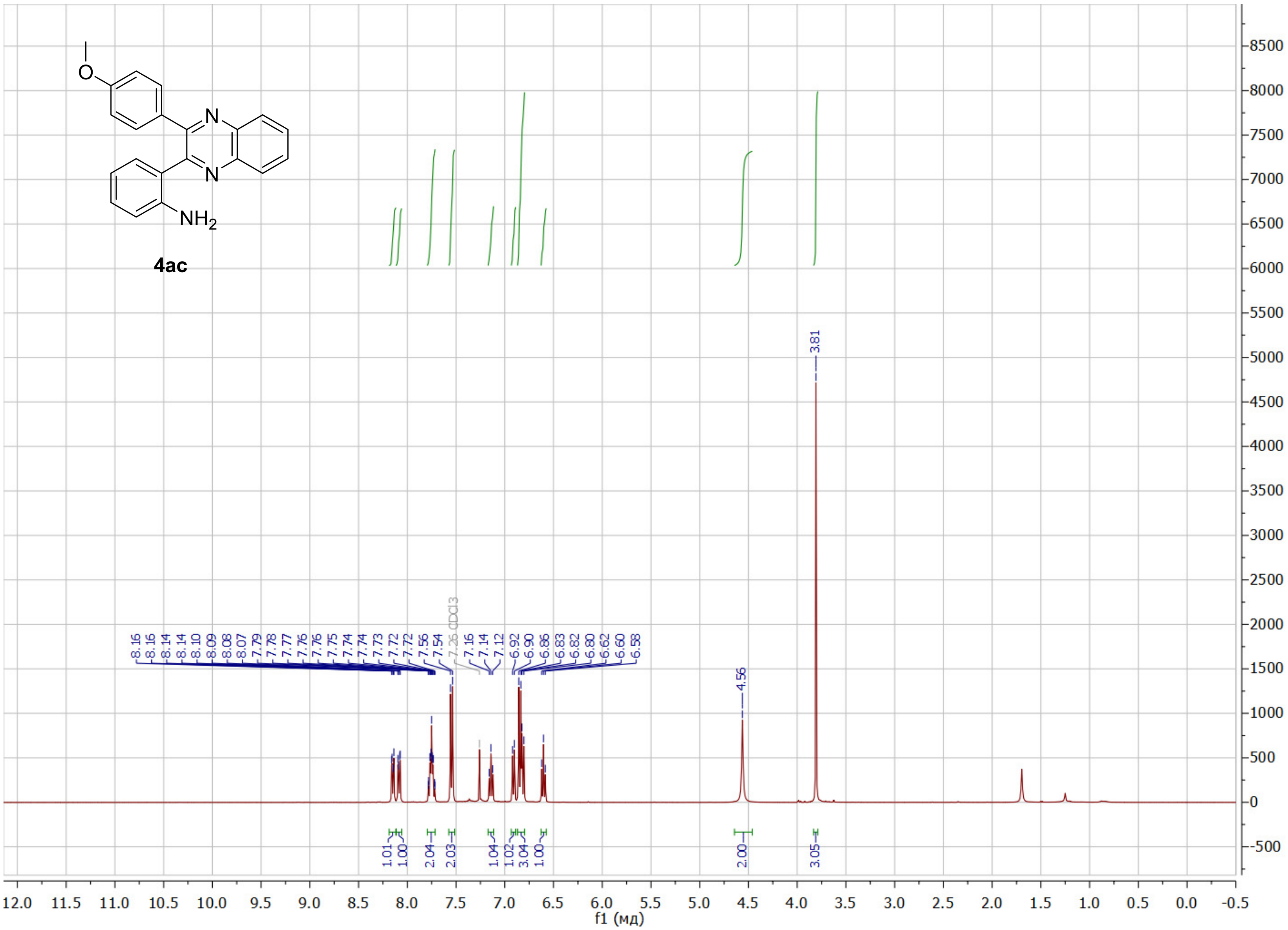

Figure S6. ^{1}H NMR spectrum of **4ac** in $CDCl_3$ (400 MHz)

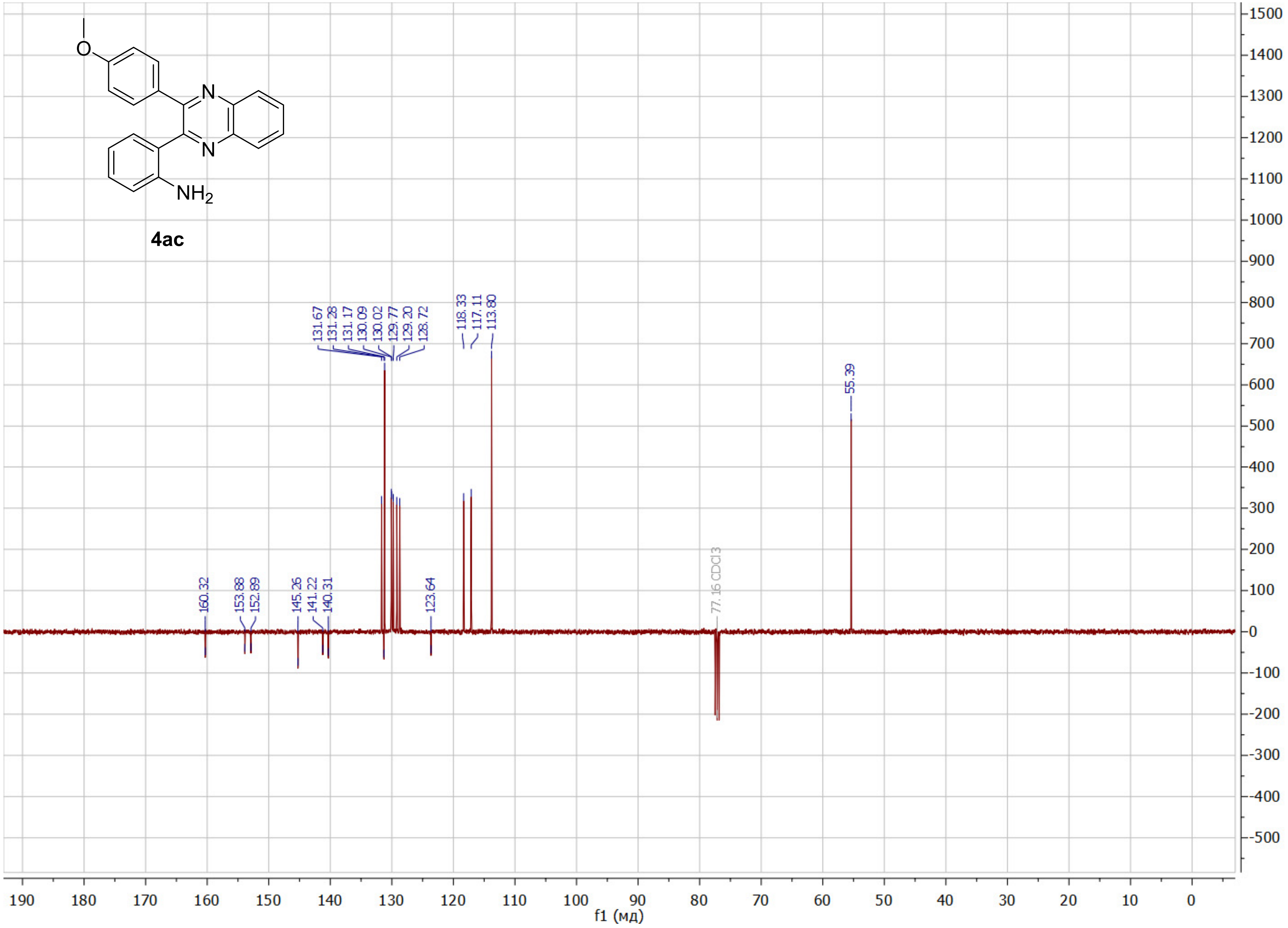

Figure S7. ^{13}C DEPTQ NMR spectrum of **4ac** in $CDCl_3$ (101 MHz)

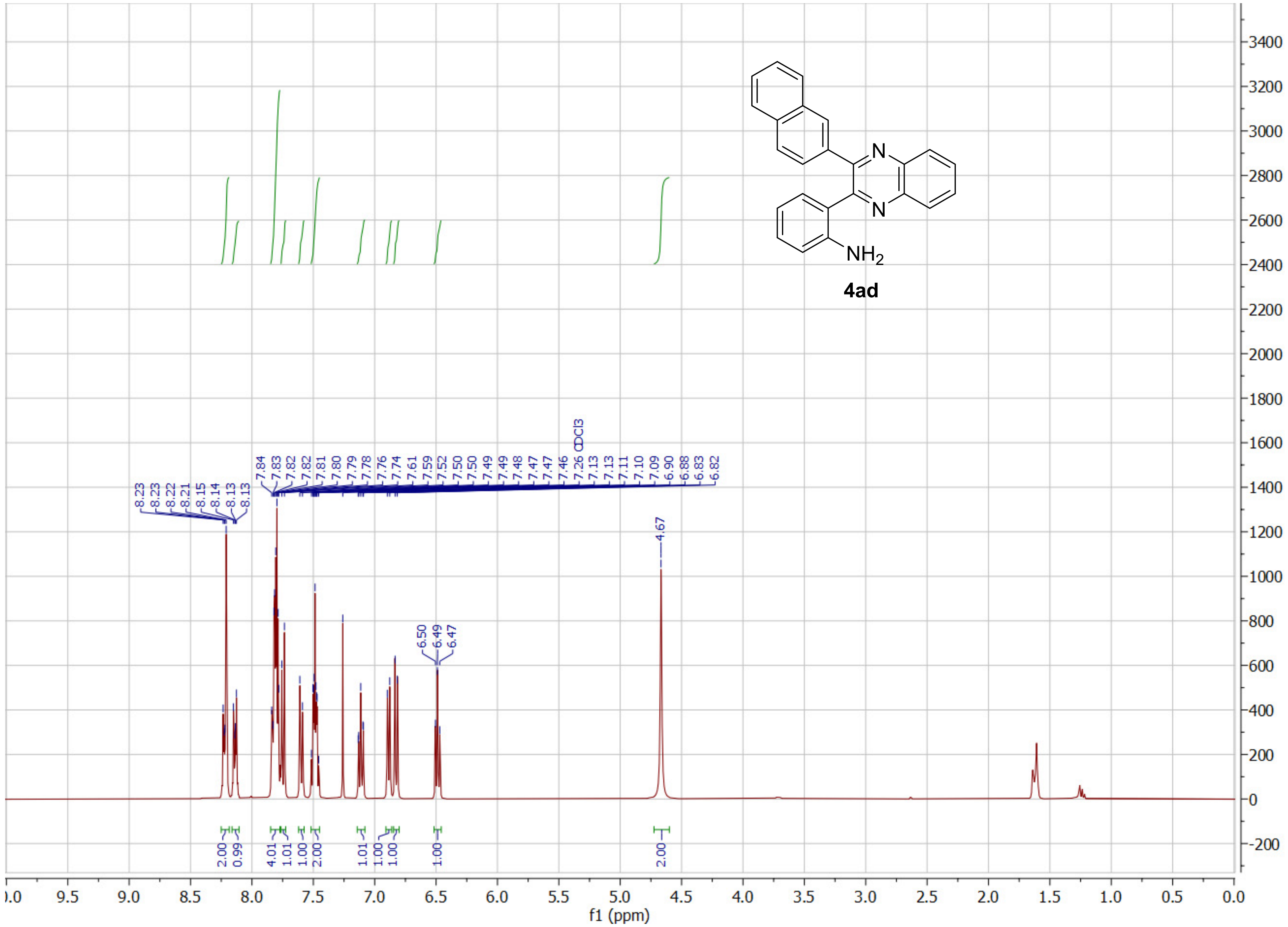

Figure S8. ^{1}H NMR spectrum of **4ad** in $CDCl_3$ (400 MHz)

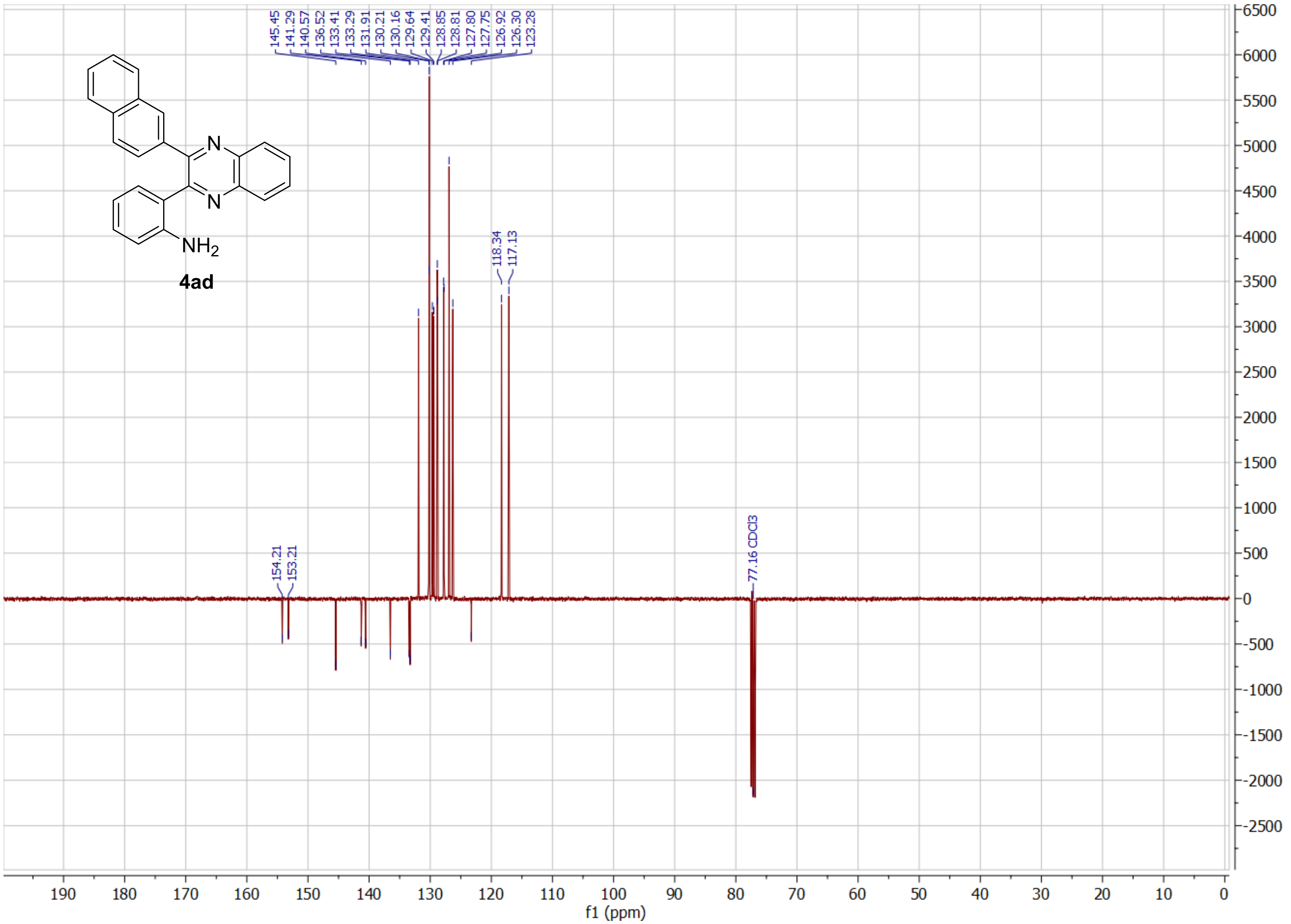

Figure S9. ^{13}C DEPTQ NMR spectrum of **4ad** in $CDCl_3$ (101 MHz)

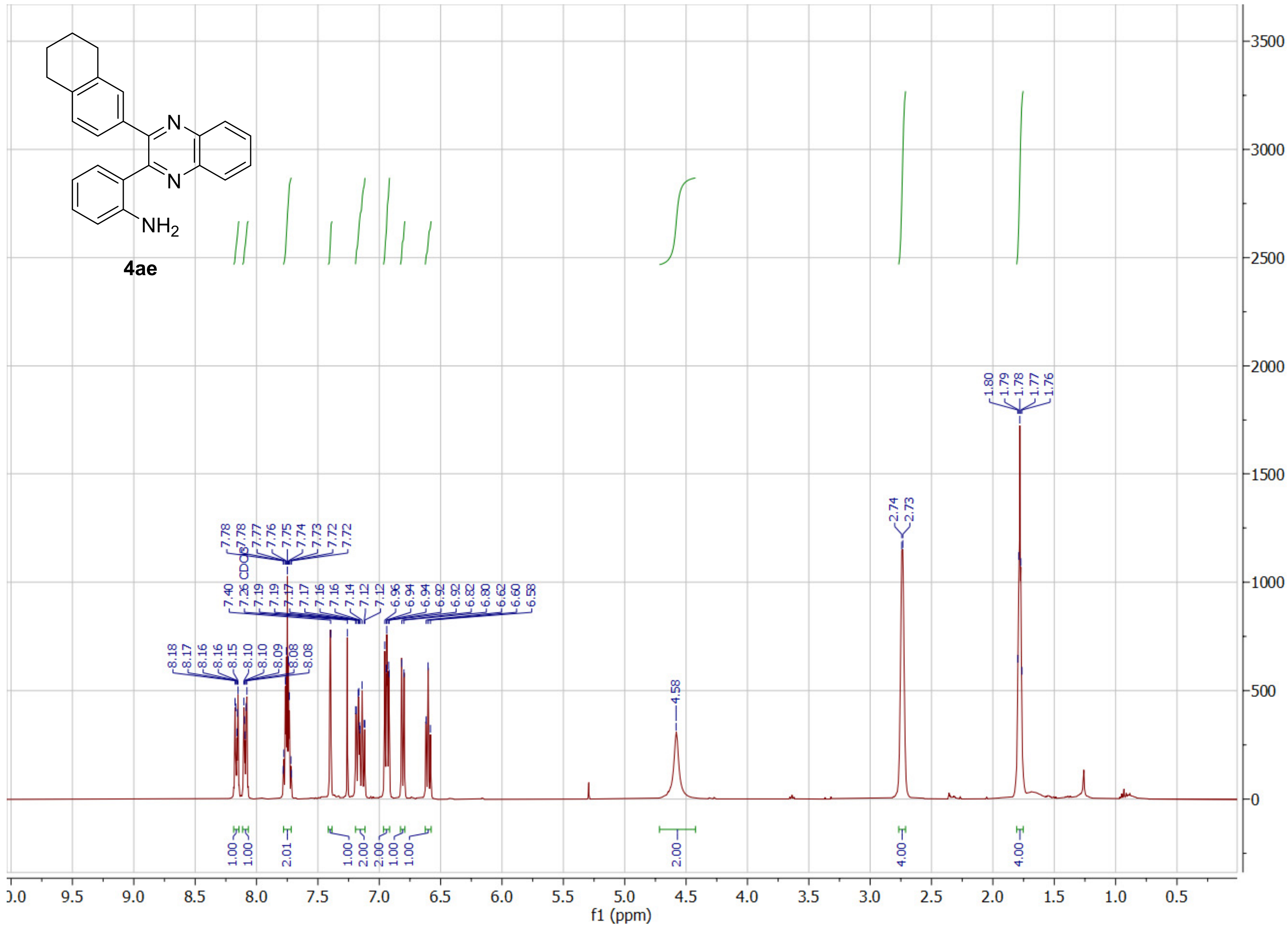

Figure S10. ^{1}H NMR spectrum of **4ae** in $CDCl_3$ (400 MHz)

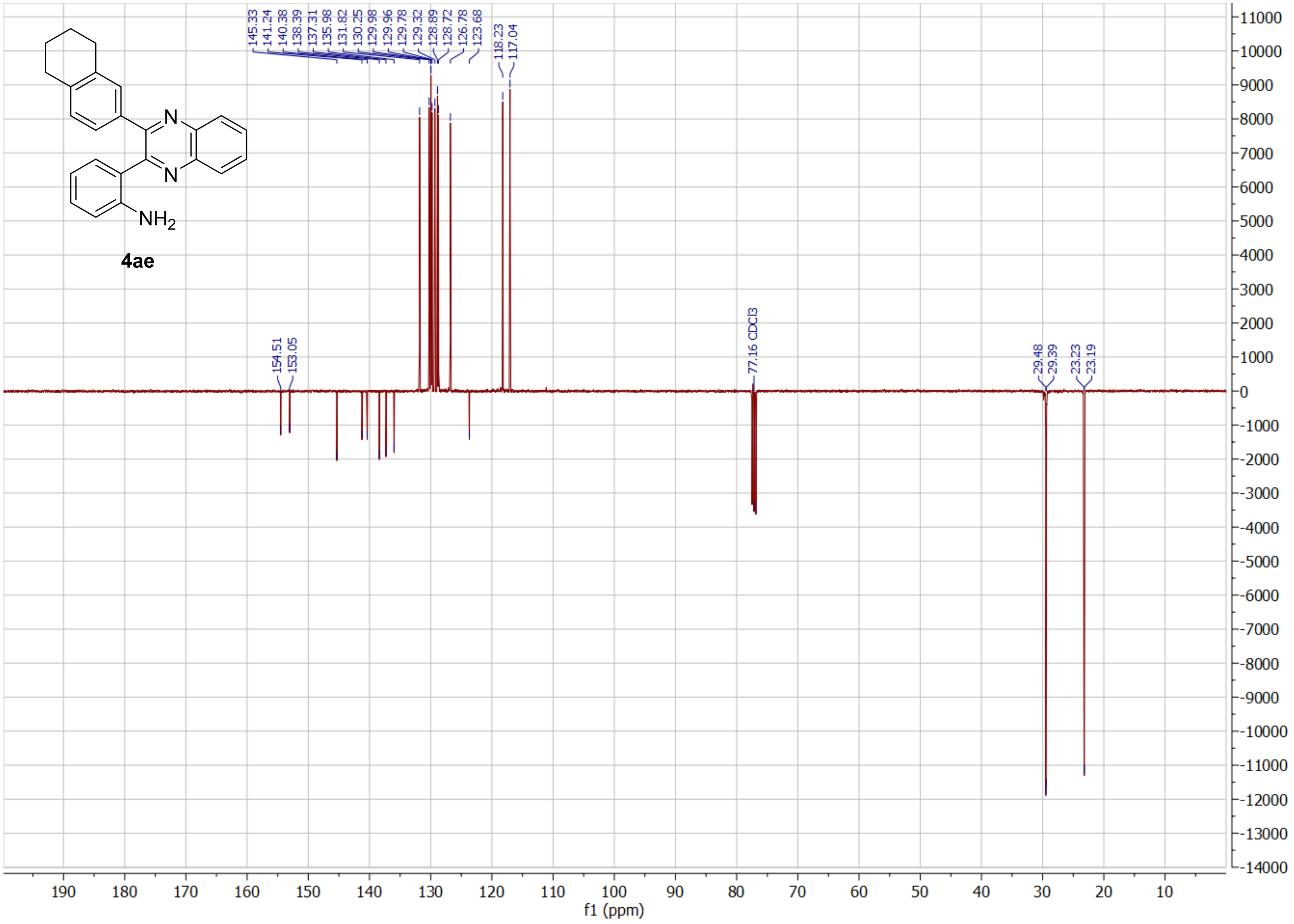

Figure S11. ^{13}C DEPTQ NMR spectrum of **4ae** in $CDCl_3$ (101 MHz)

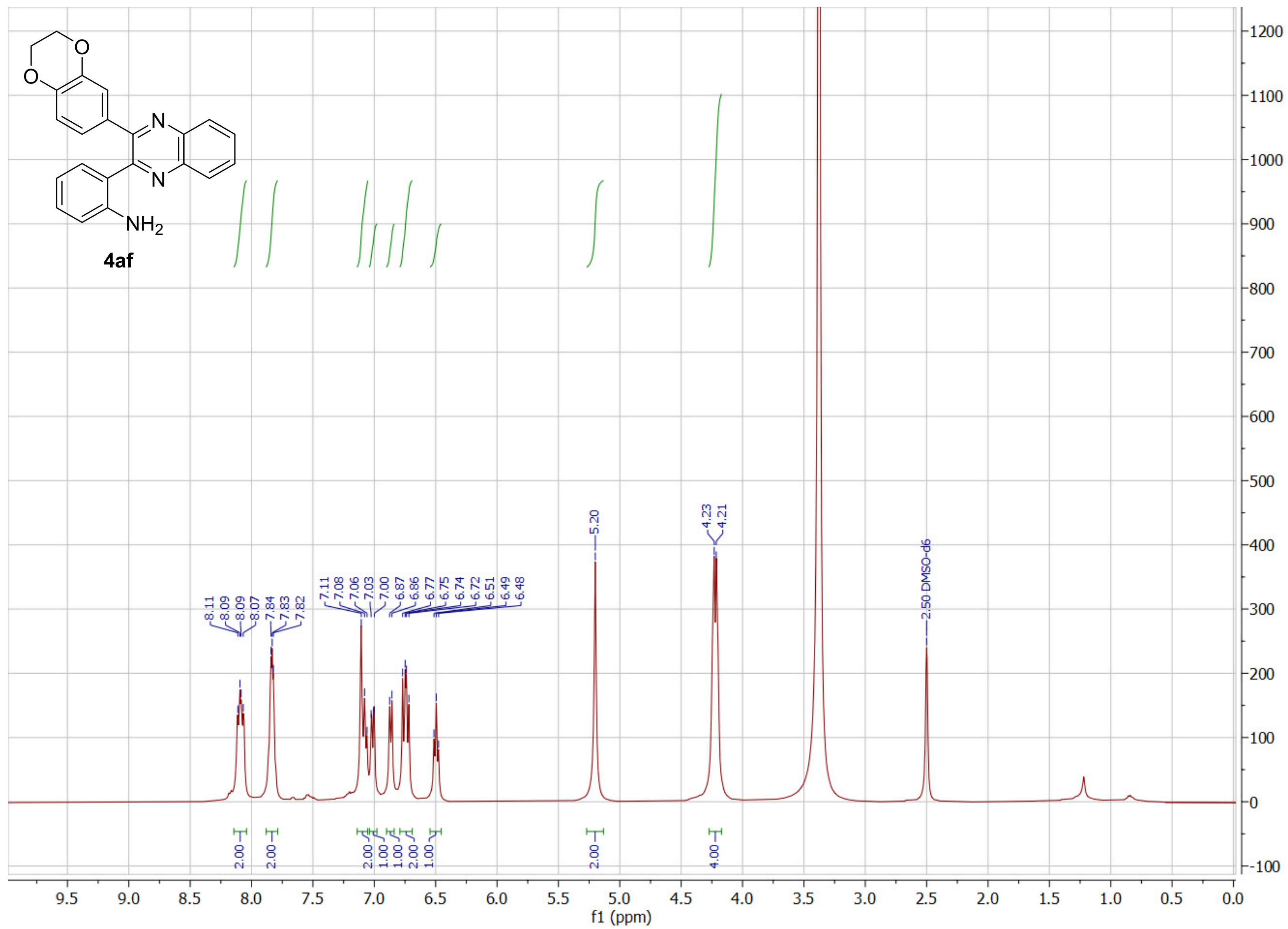

Figure S12. ^{1}H NMR spectrum of **4af** in DMSO-d_6 (400 MHz)

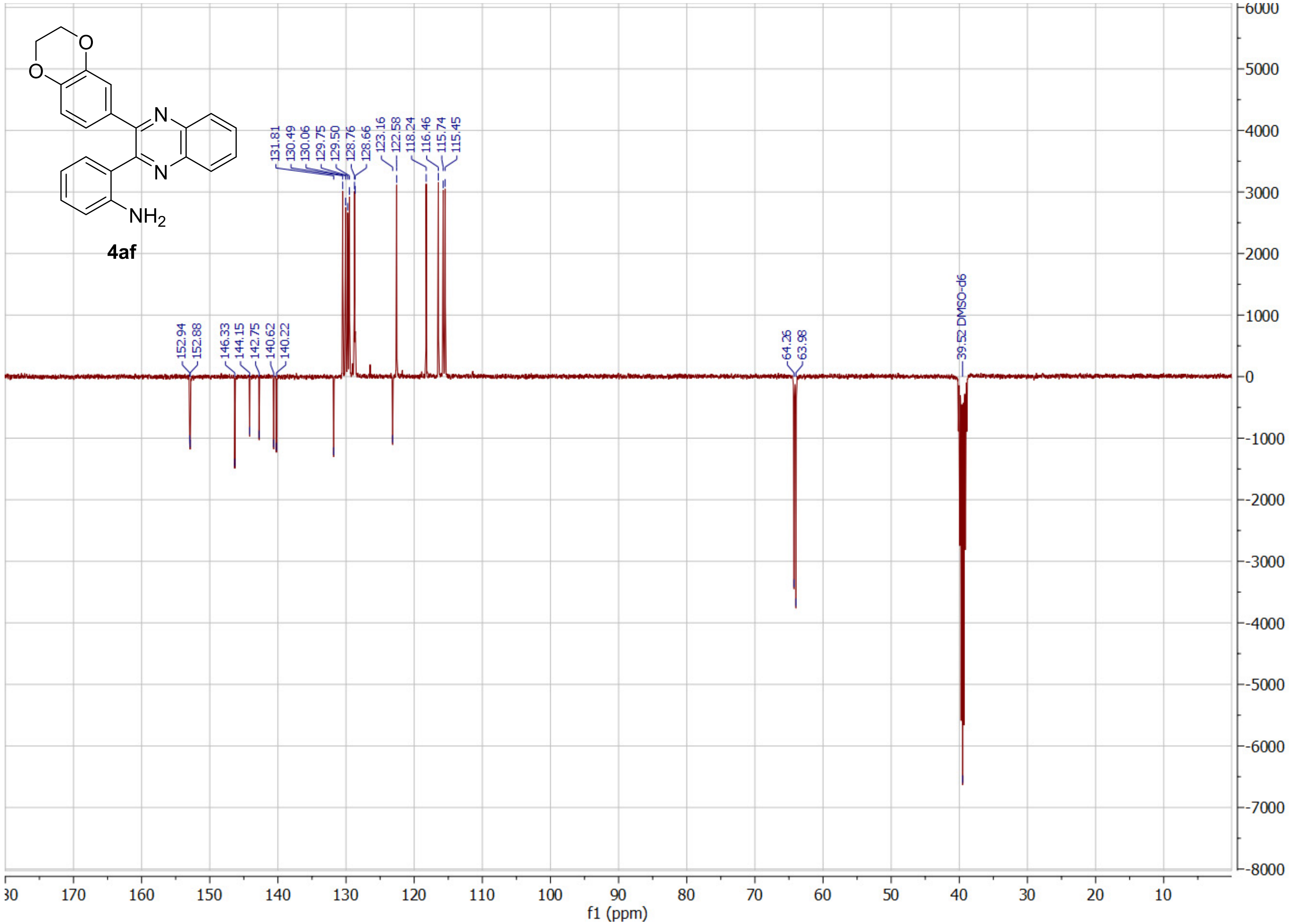

Figure S13. ^{13}C DEPTQ NMR spectrum of **4af** in DMSO-d_6 (101 MHz)

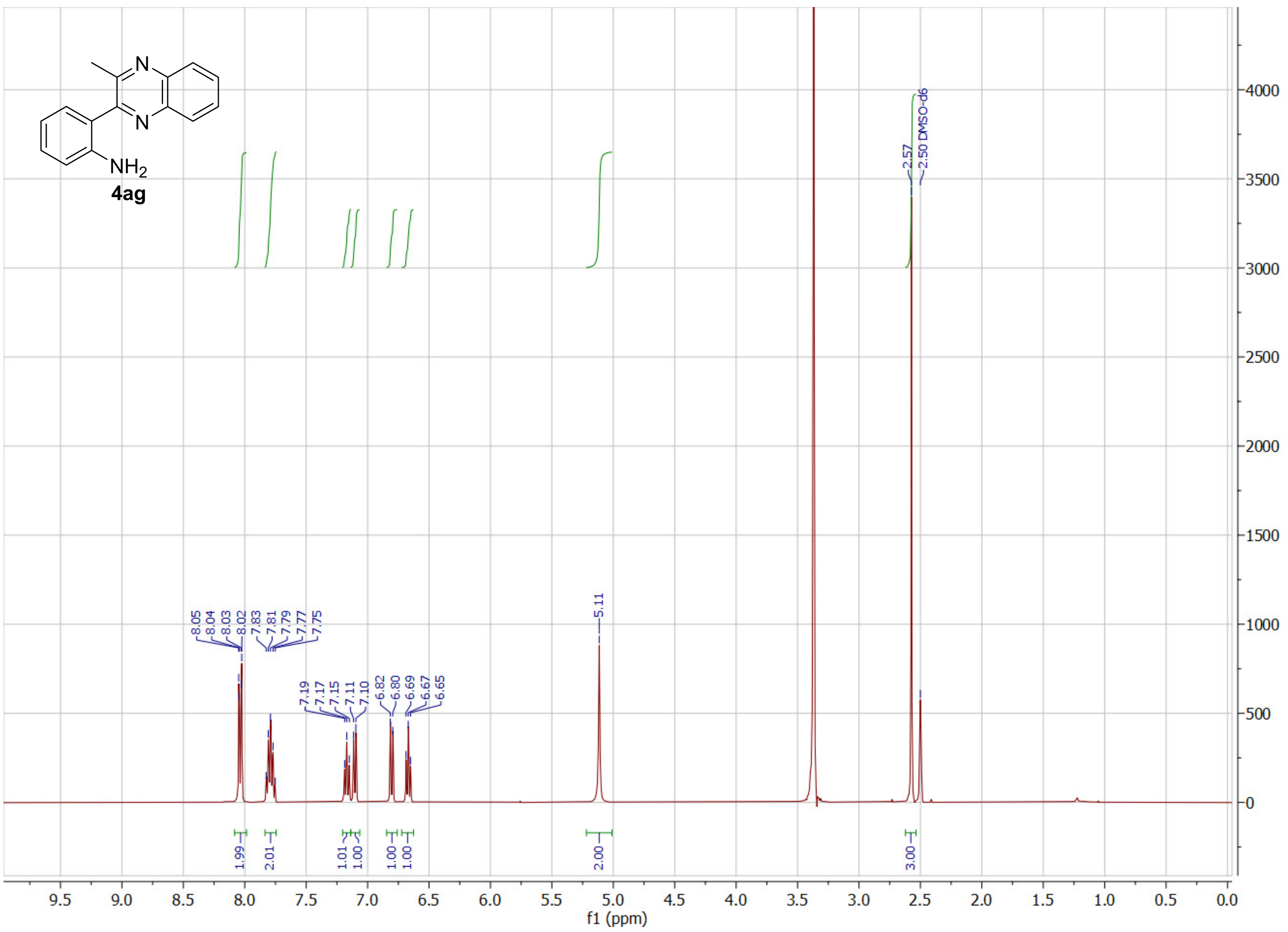

Figure S14. ^{1}H NMR spectrum of **4ag** in DMSO-d_6 (400 MHz)

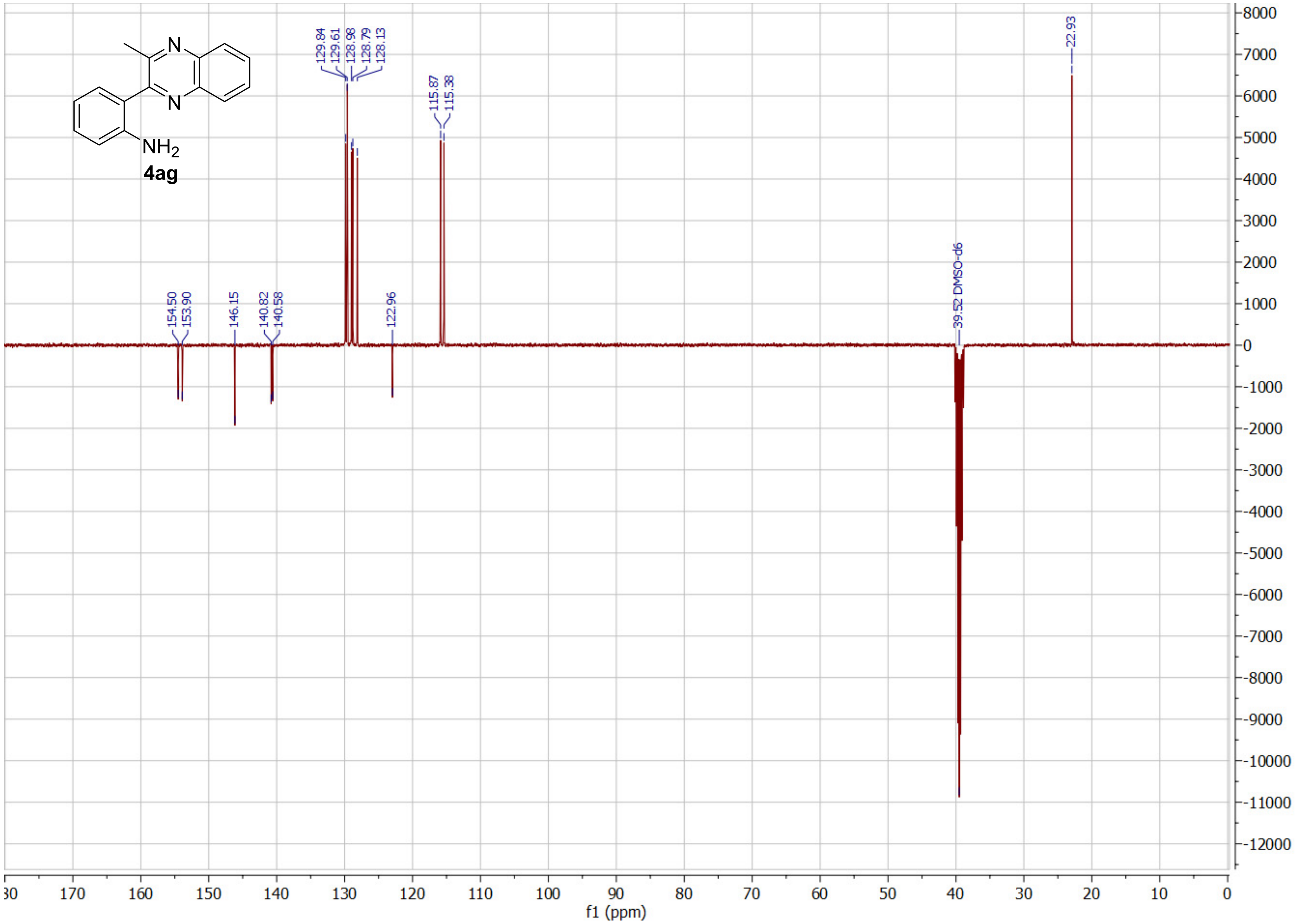

Figure S15. ^{13}C DEPTQ NMR spectrum of **4ag** in DMSO-d_6 (101 MHz)

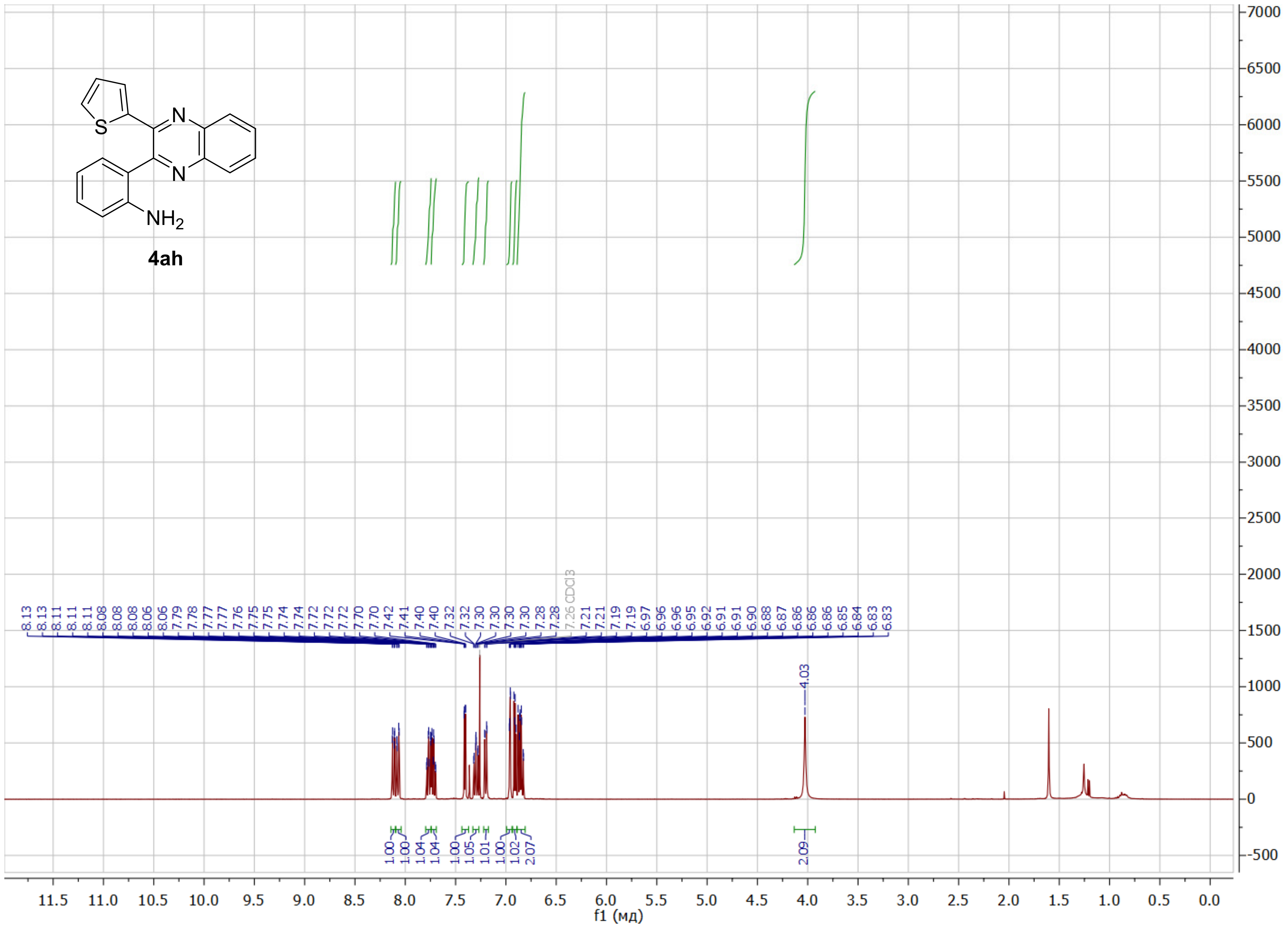

Figure S16. ^{1}H NMR spectrum of **4ah** in $CDCl_3$ (400 MHz)

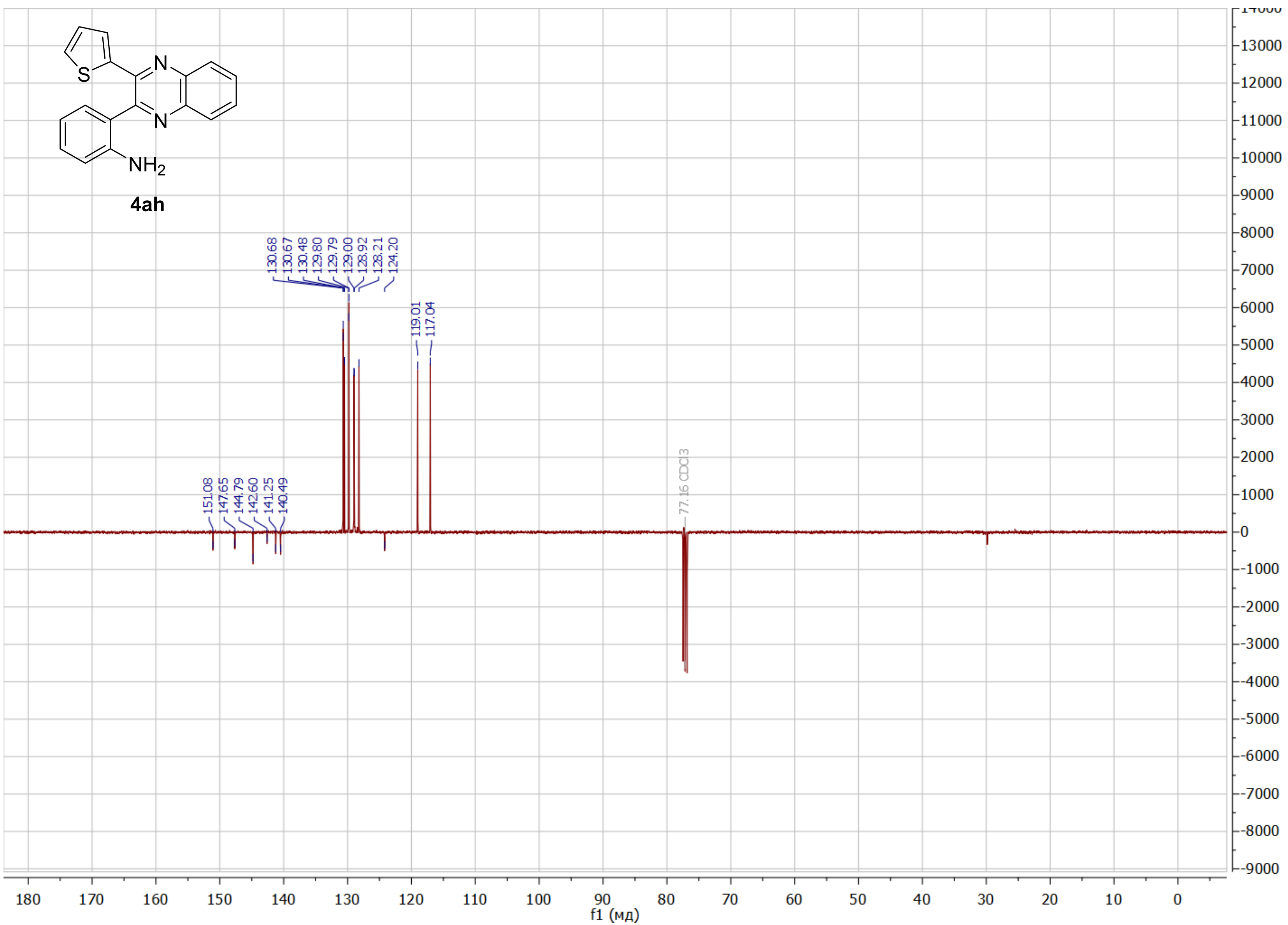

Figure S17. ^{13}C DEPTQ NMR spectrum of **4ah** in $CDCl_3$ (101 MHz)

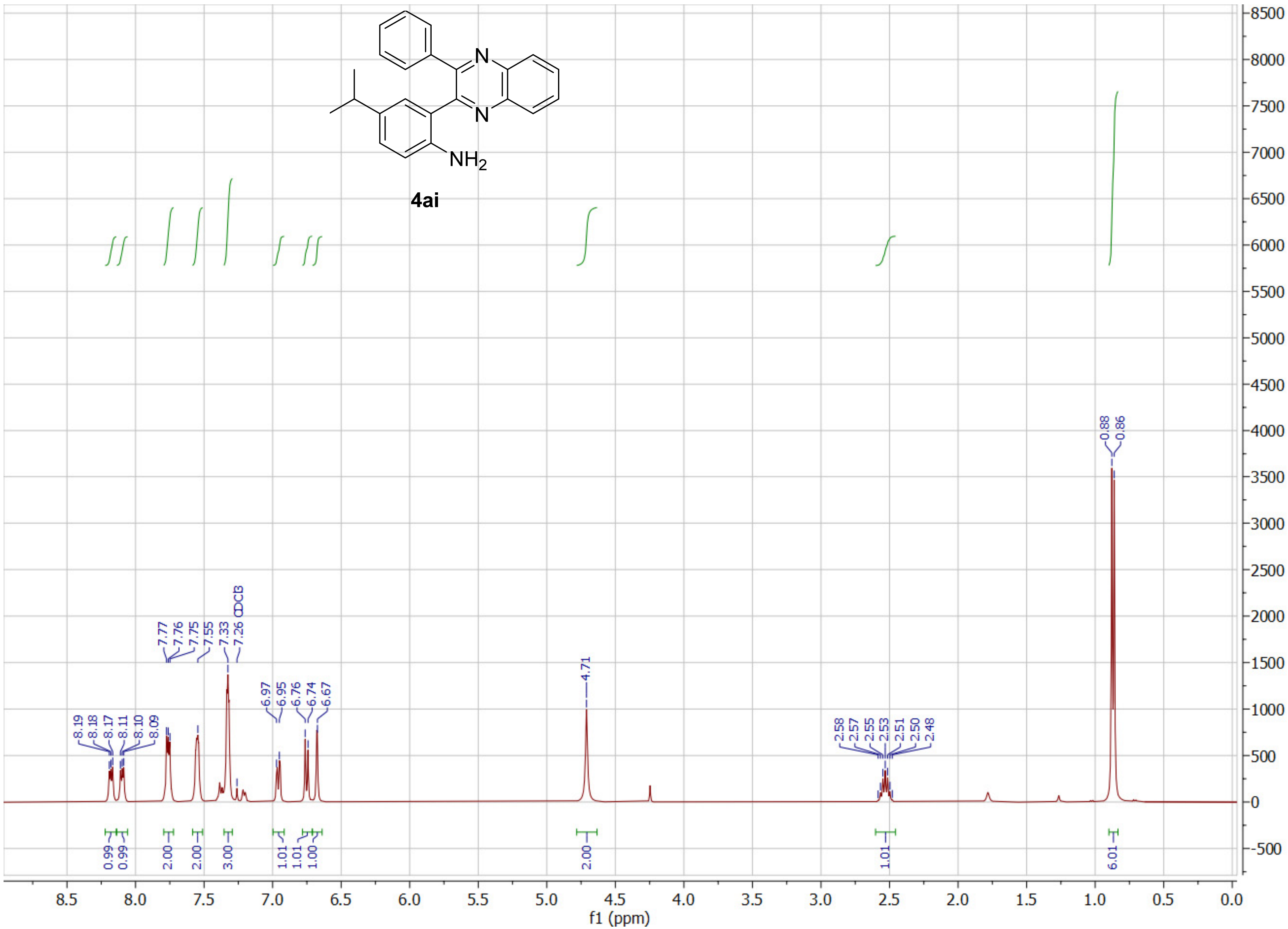

Figure S18. ^{1}H NMR spectrum of **4ai** in $CDCl_3$ (400 MHz)

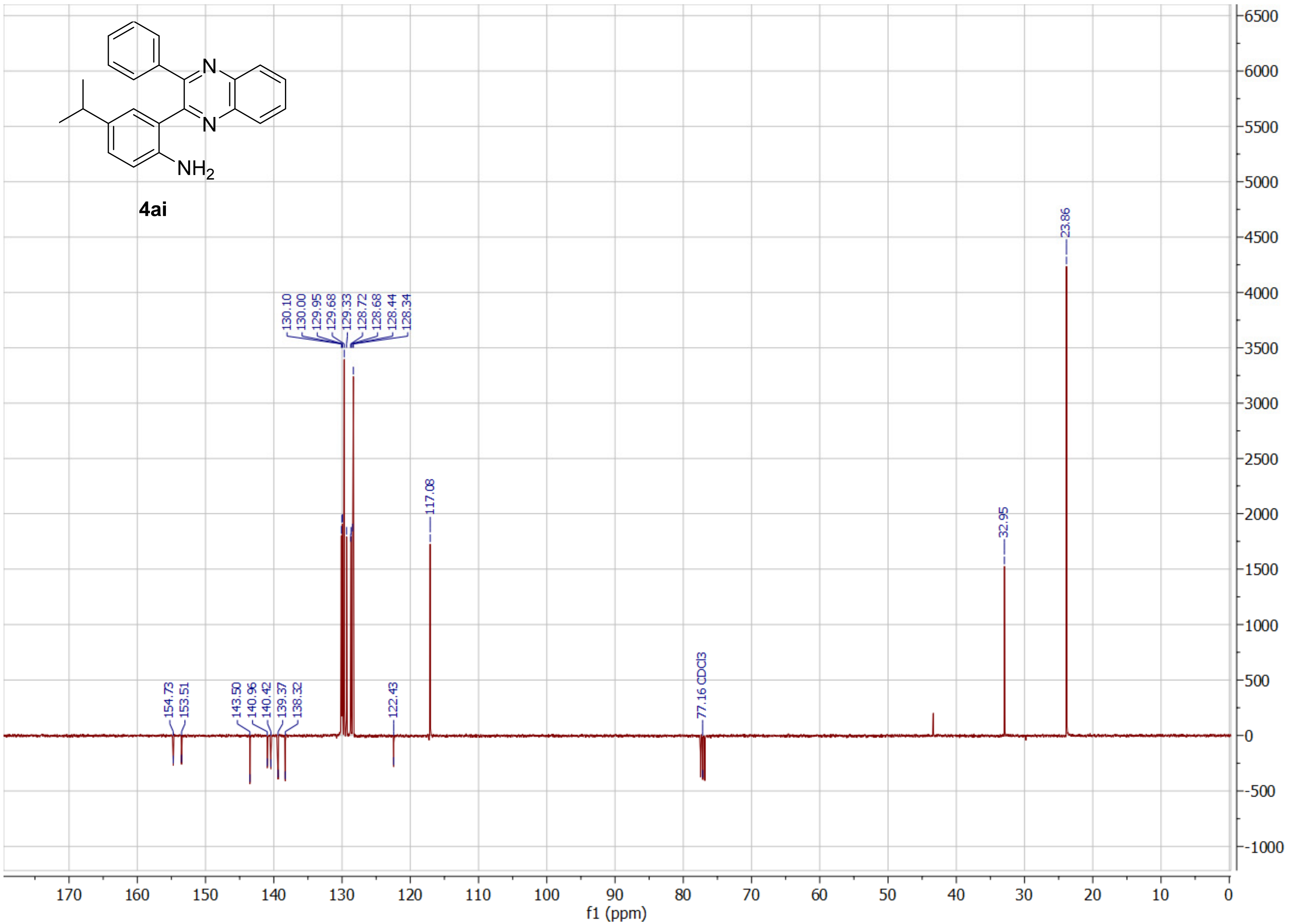

Figure S19. ^{13}C DEPTQ NMR spectrum of **4ai** in $CDCl_3$ (101 MHz)

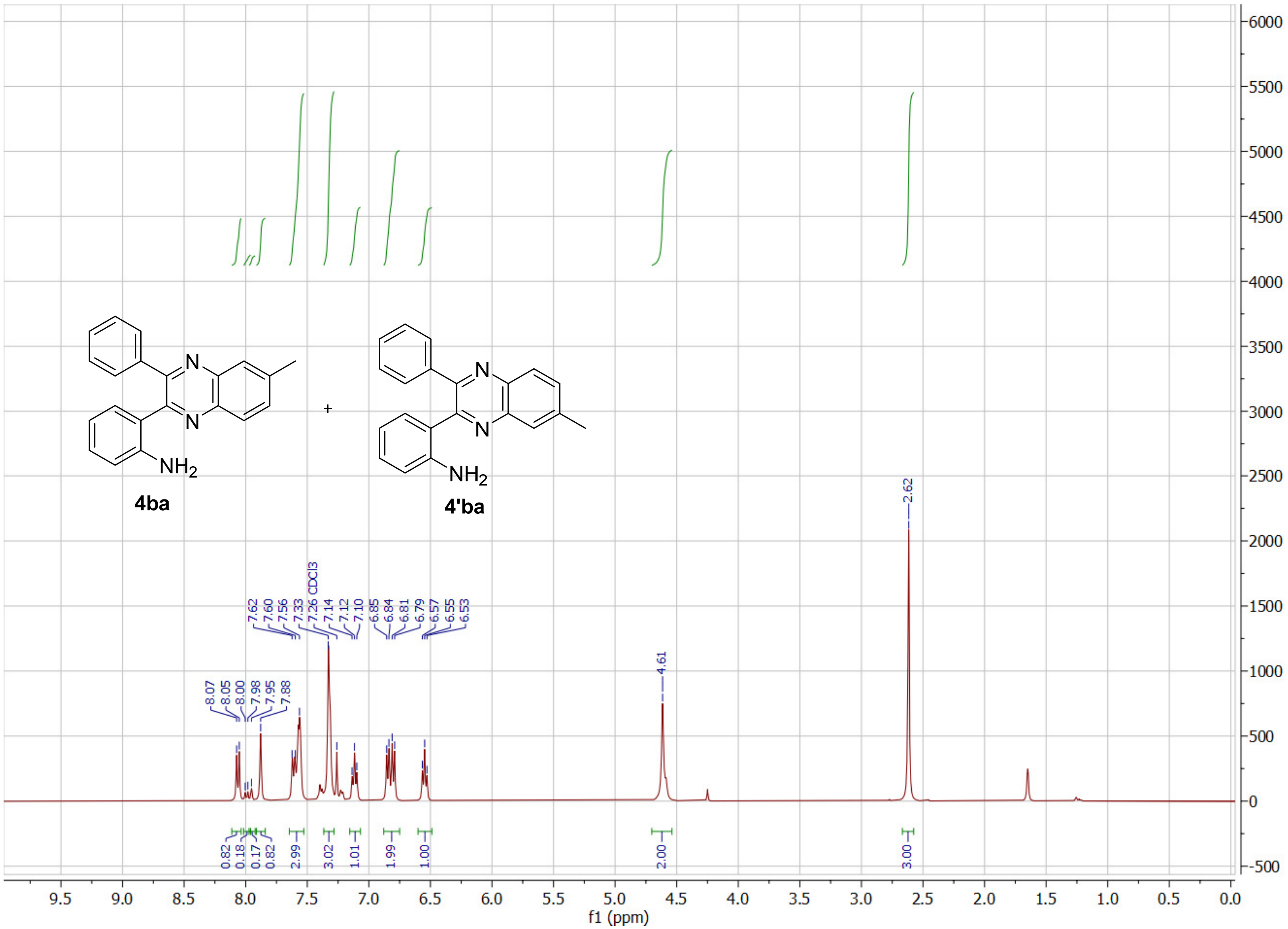

Figure S20. ^{1}H NMR spectrum of **4ba+4’ba** in $CDCl_3$ (400 MHz)

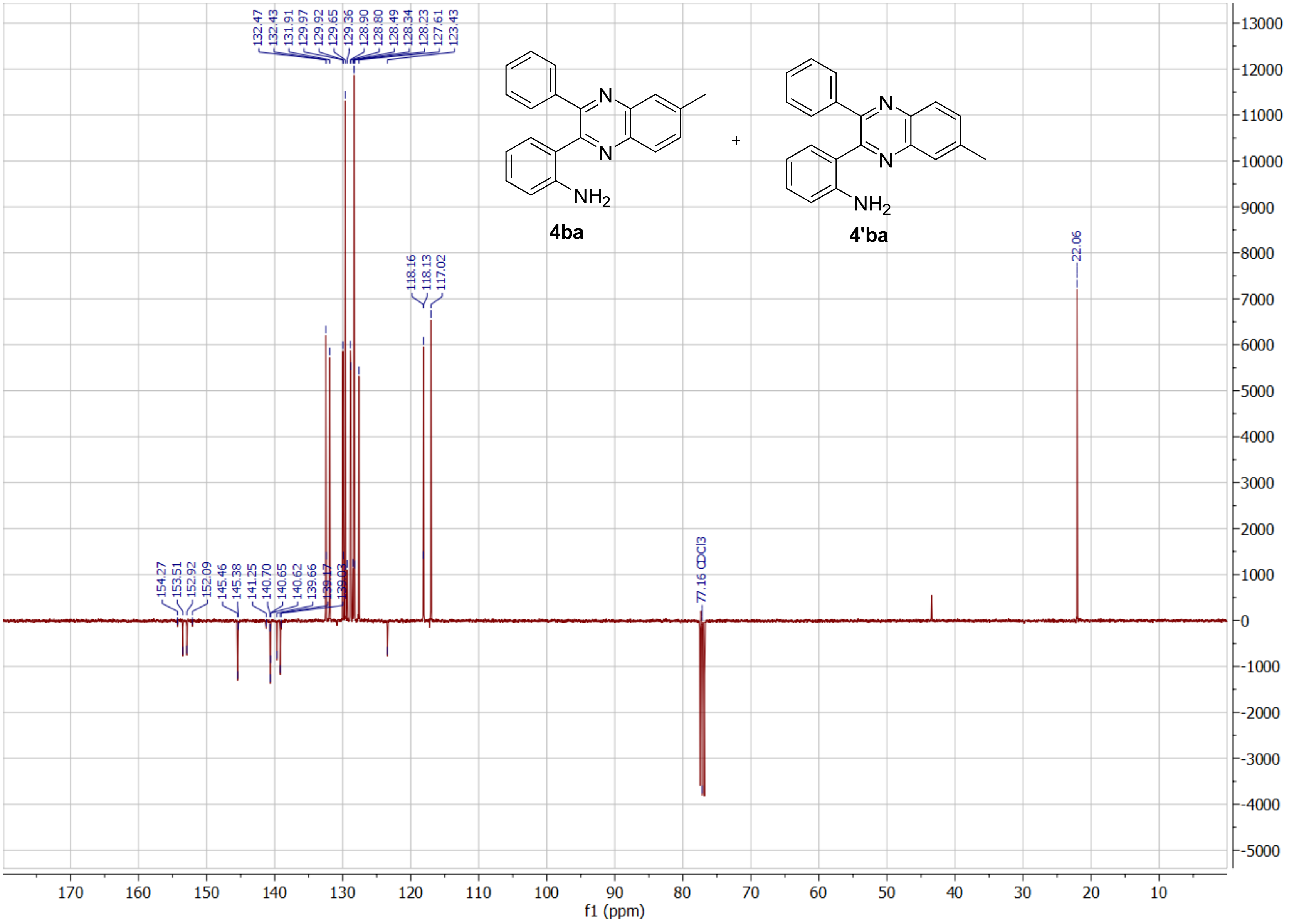

Figure S21. ^{13}C DEPTQ NMR spectrum of **4ba+4’ba** in $CDCl_3$ (101 MHz)

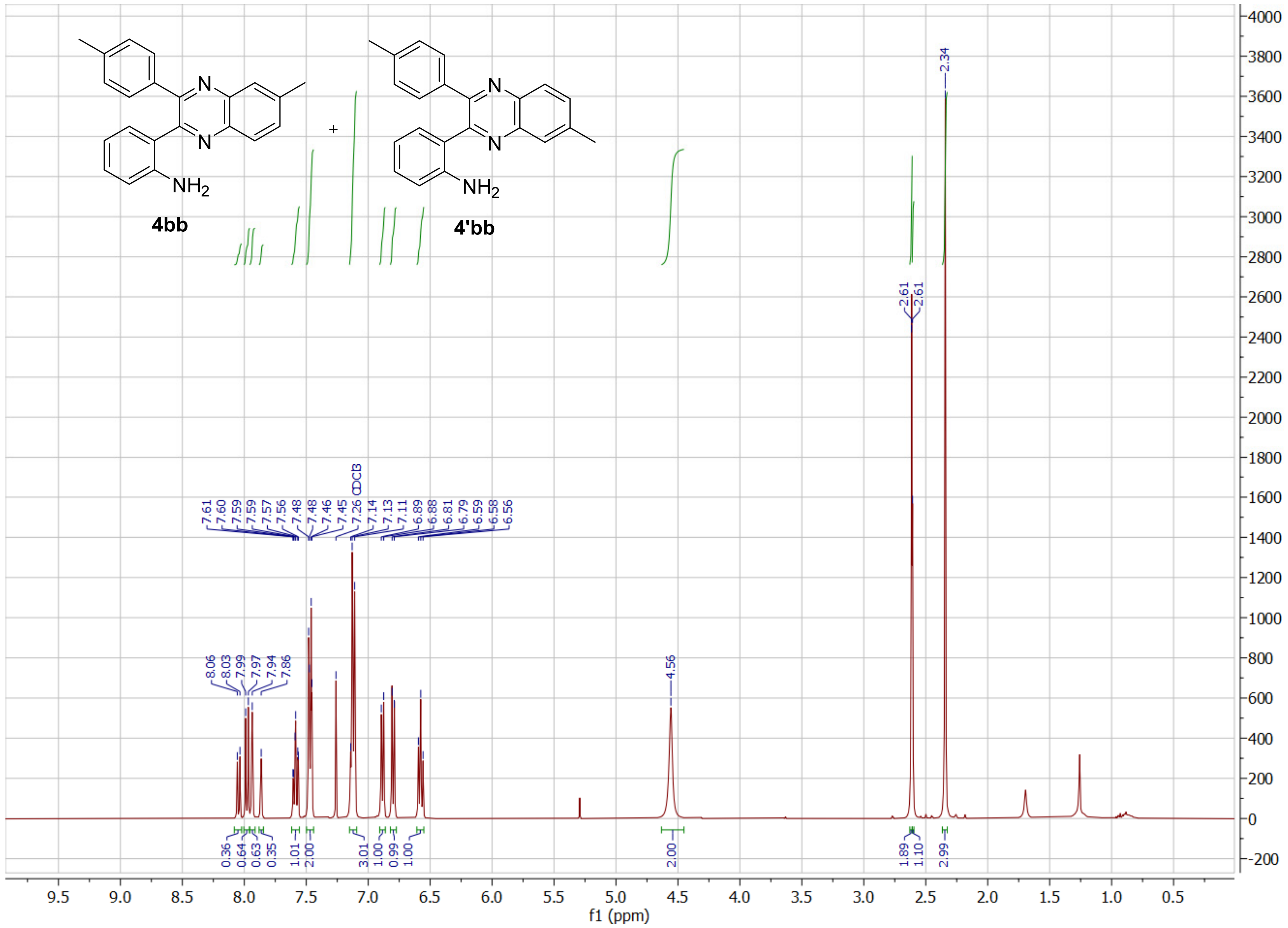

Figure S22. ^{1}H NMR spectrum of **4bb+4’bb** in $CDCl_3$ (400 MHz)

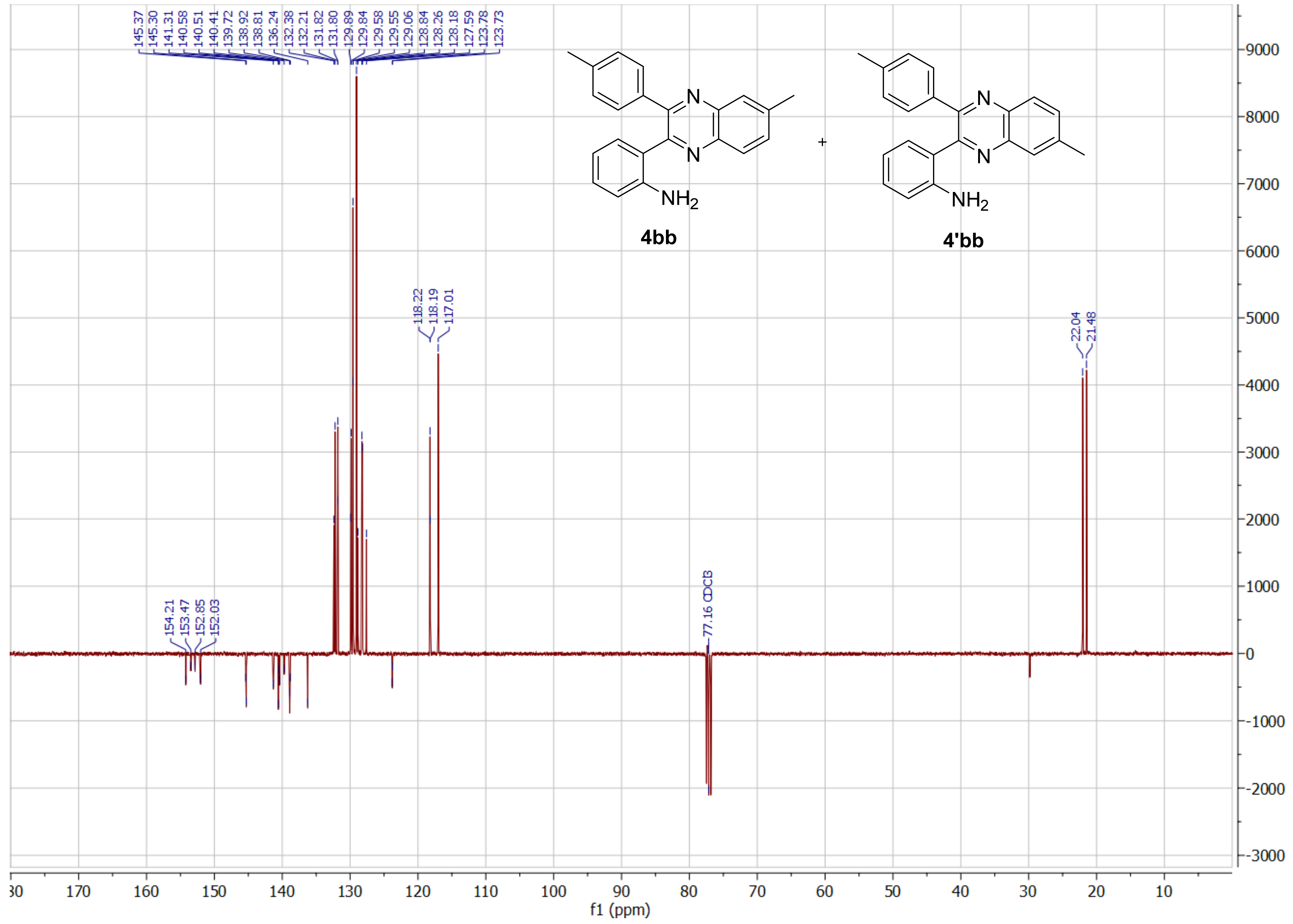

Figure S23. ^{13}C DEPTQ NMR spectrum of **4bb+4’bb** in $CDCl_3$ (101 MHz)

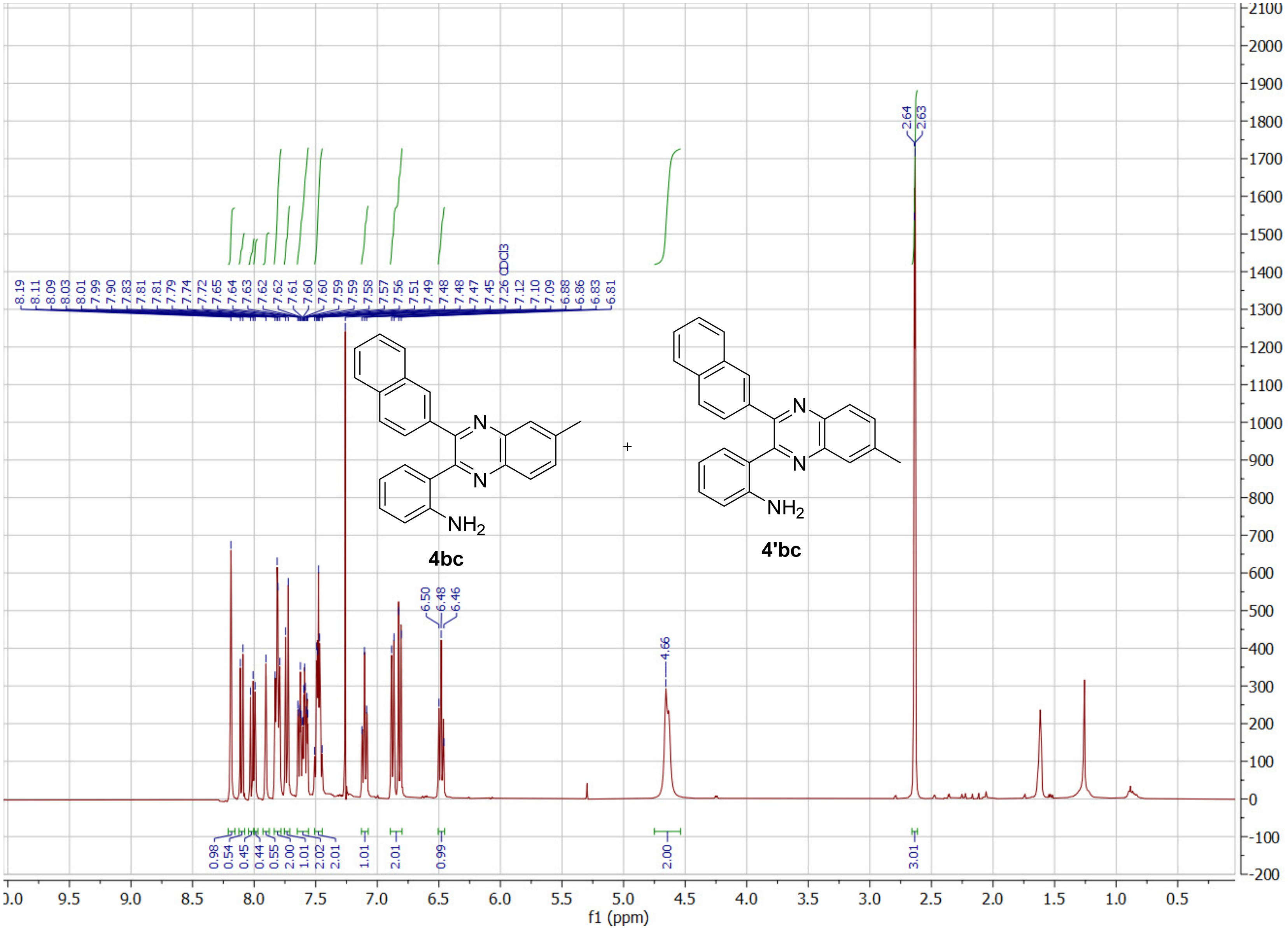

Figure S24. ^{1}H NMR spectrum of **4bc+4’bc** in $CDCl_3$ (400 MHz)

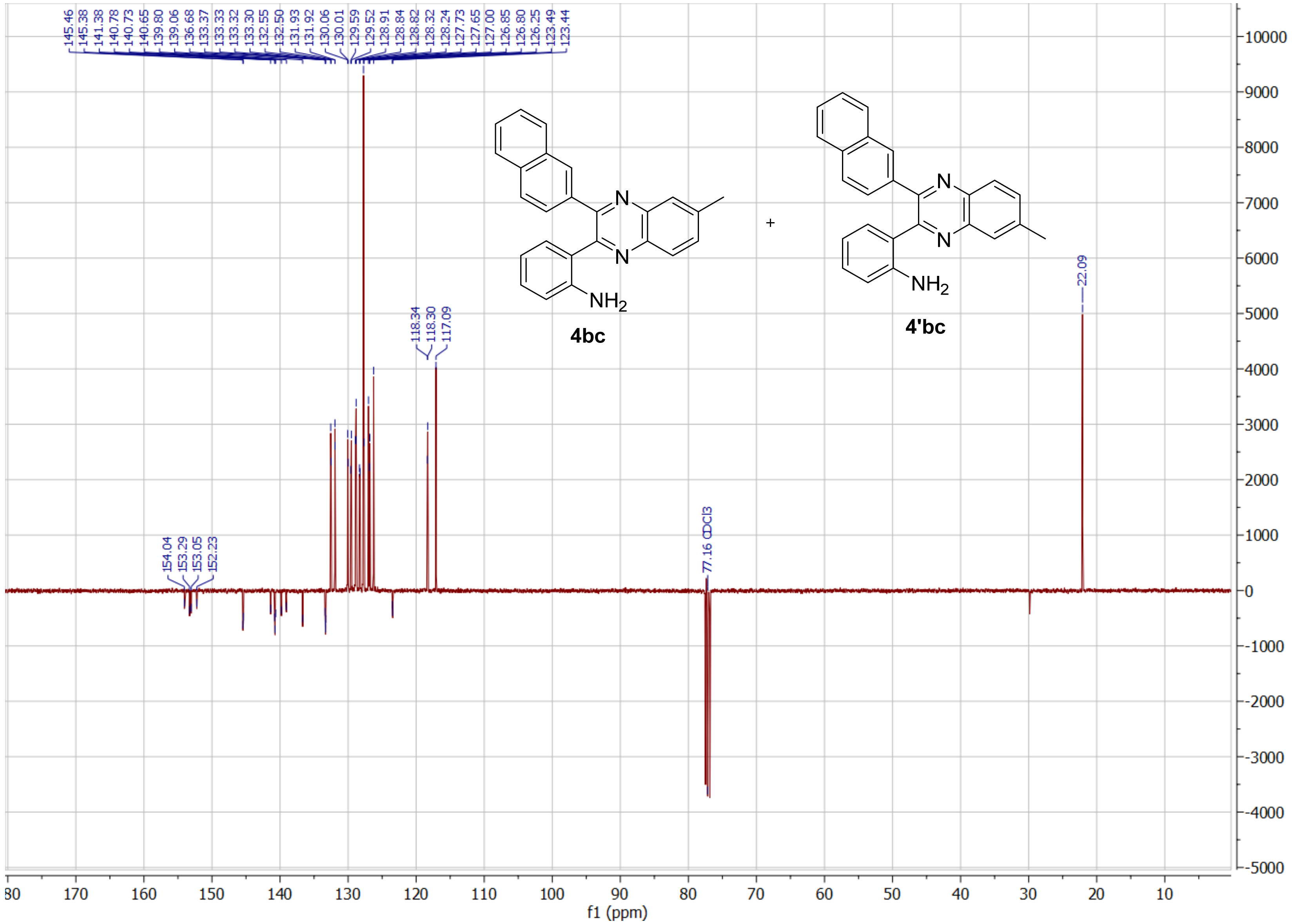

Figure S25. ^{13}C DEPTQ NMR spectrum of **4bc+4’bc** in $CDCl_3$ (101 MHz)

HRMS spectral charts

HRMS spectral charts for quinoxalines (4)

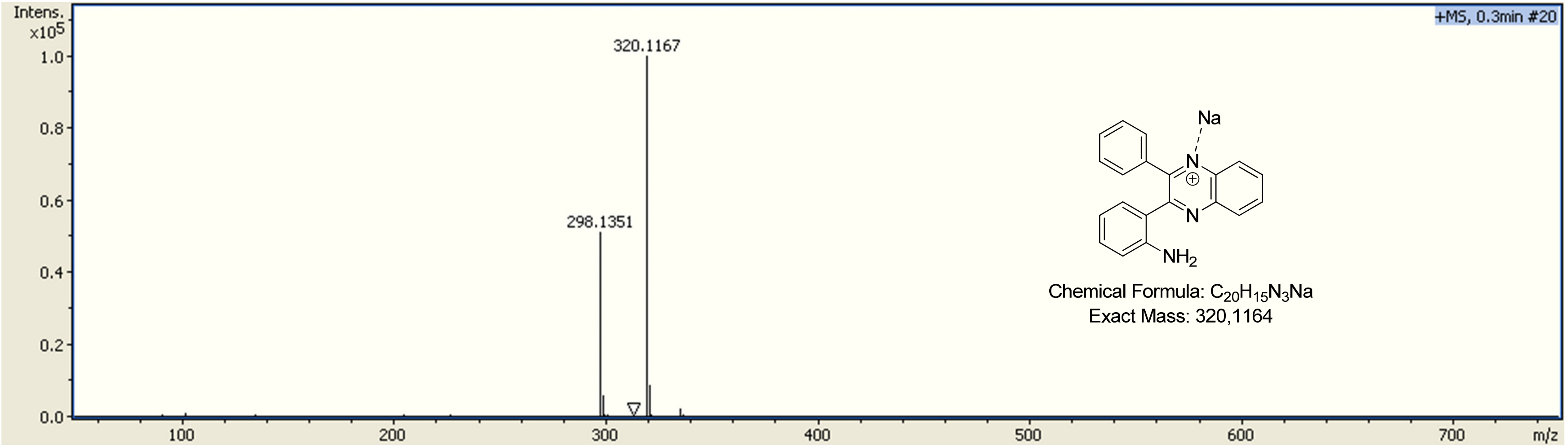

Figure S26. HRMS spectral chart for **4aa**

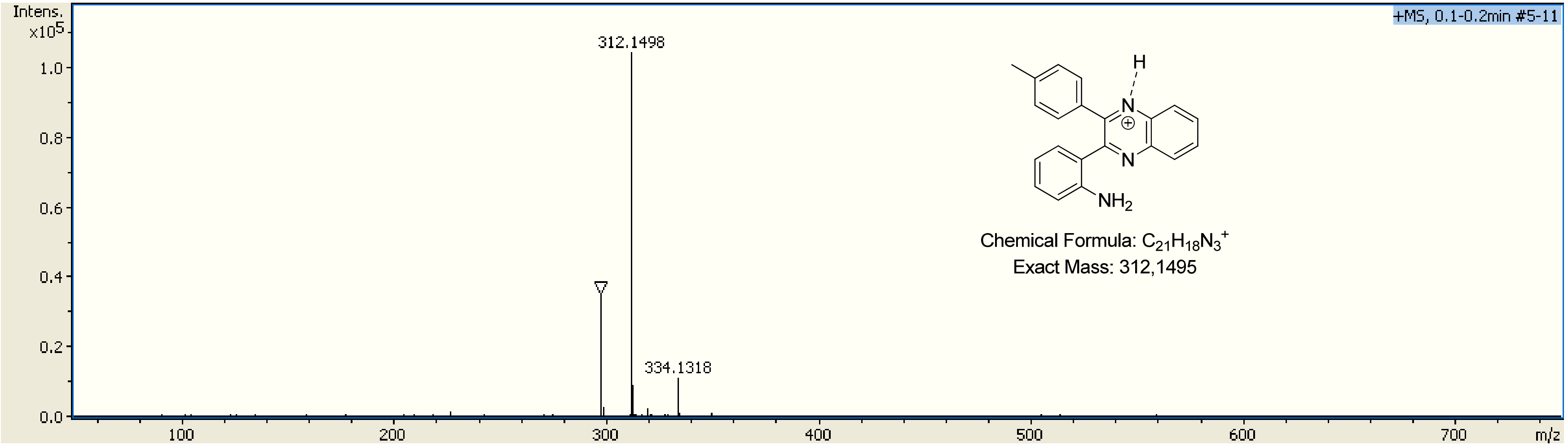

Figure S27. HRMS spectral chart for **4ab**

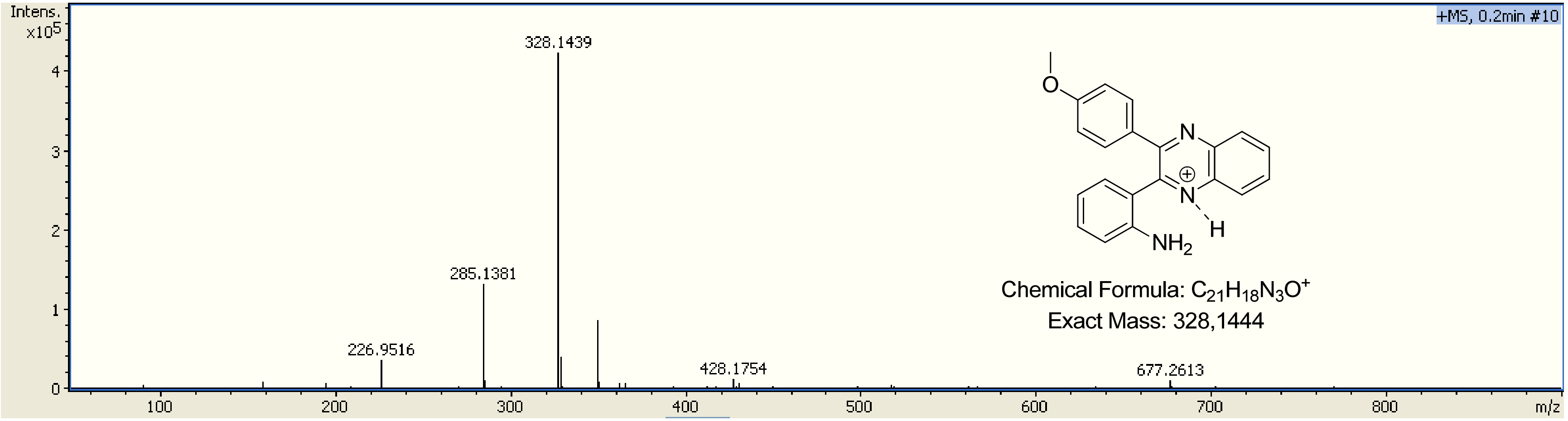

Figure S28. HRMS spectral chart for **4ac**

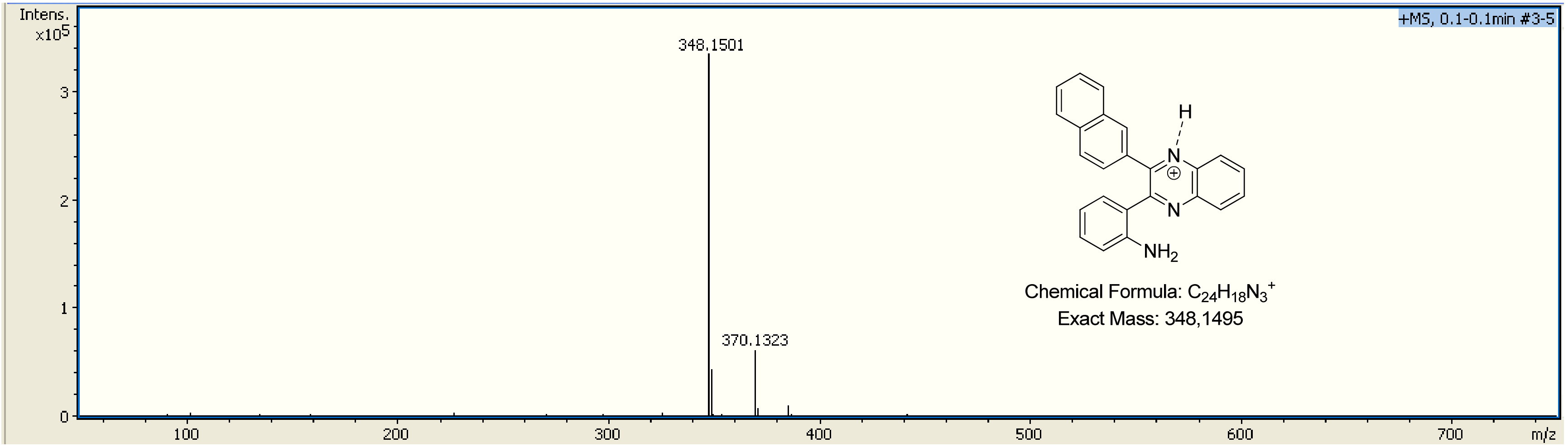

Figure S29. HRMS spectral chart for **4ad**

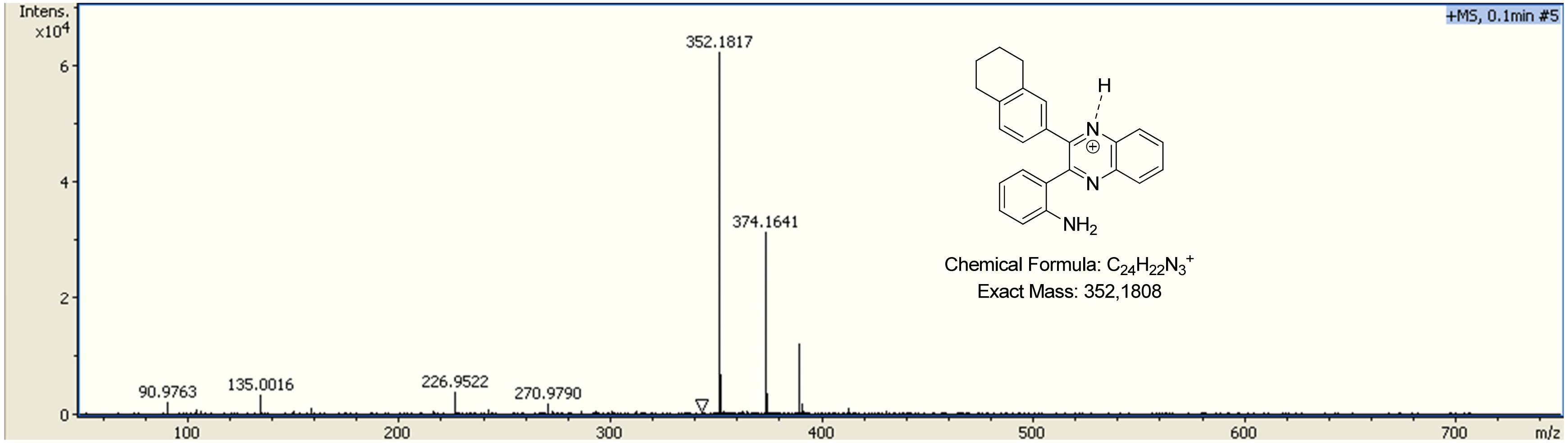

Figure S30. HRMS spectral chart for **4ae**

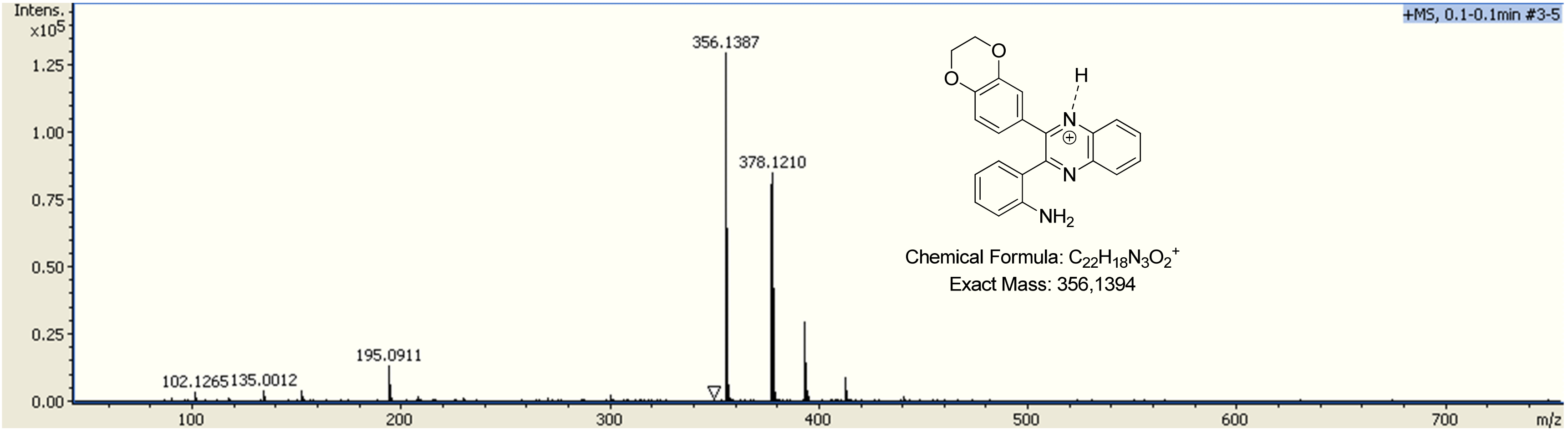

Figure S31. HRMS spectral chart for **4af**

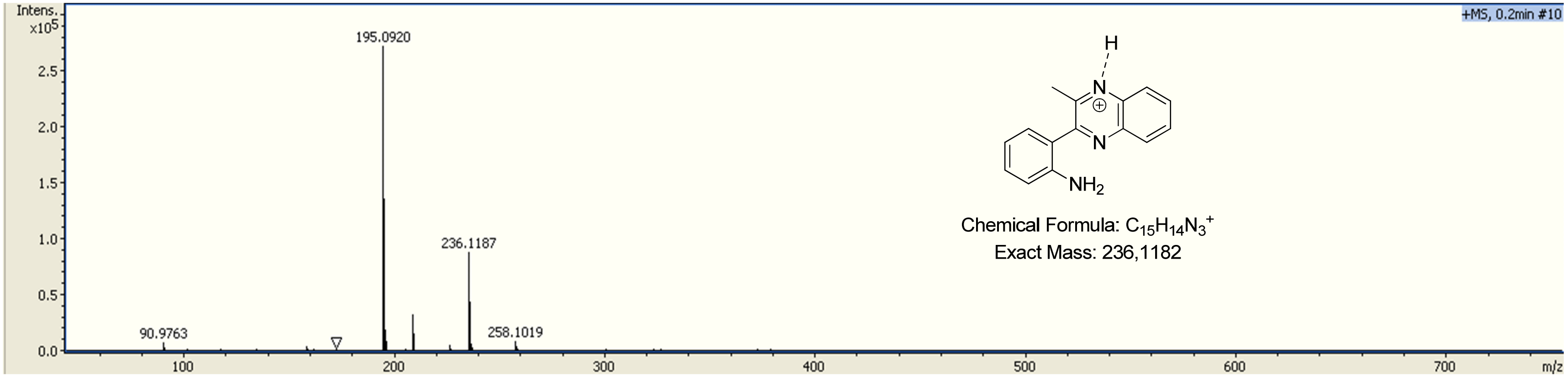

Figure S32. HRMS spectral chart for **4ag**

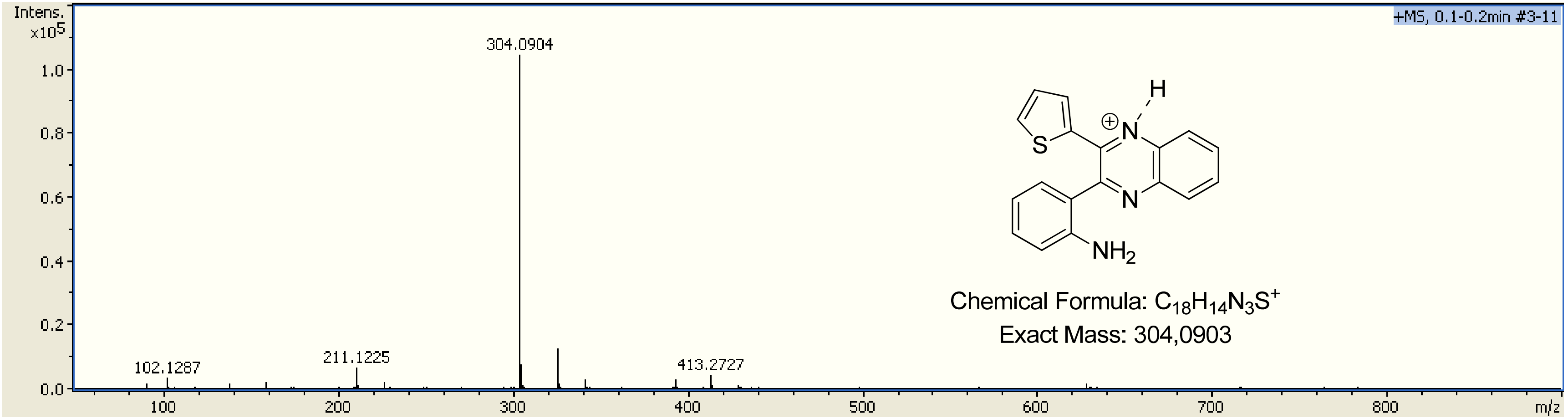

Figure S33. HRMS spectral chart for **4ah**

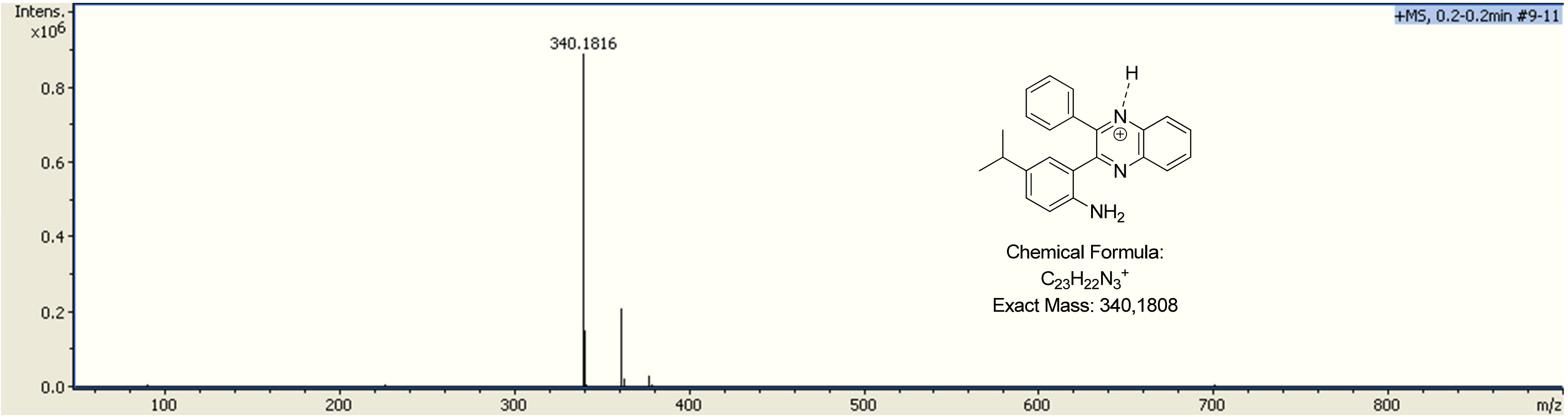

Figure S34. HRMS spectral chart for **4ai**

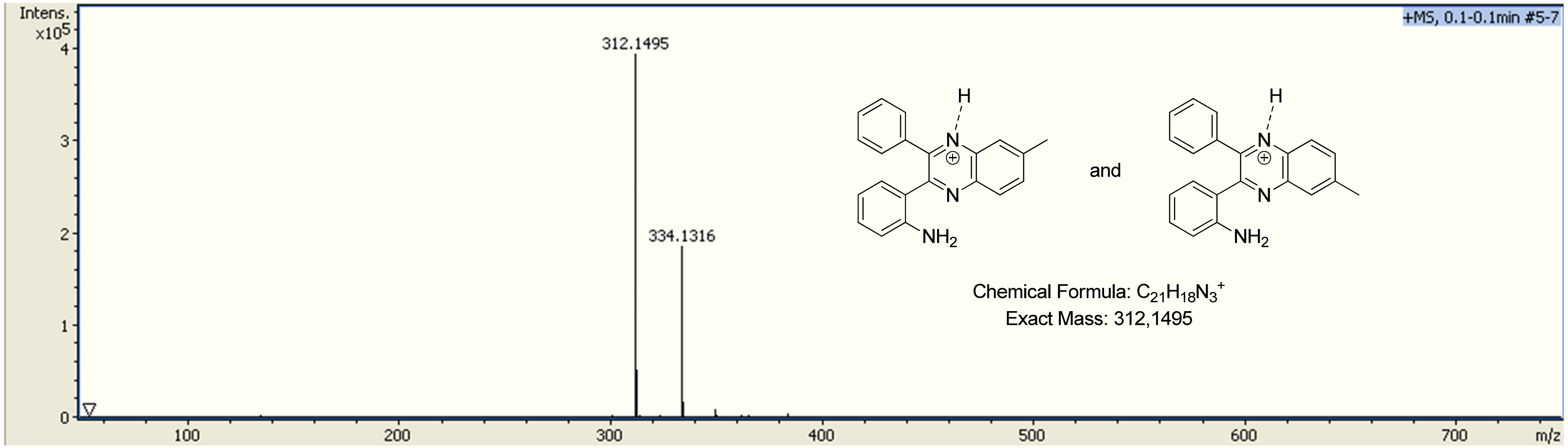

Figure S35. HRMS spectral chart for **4ba+4’ba**

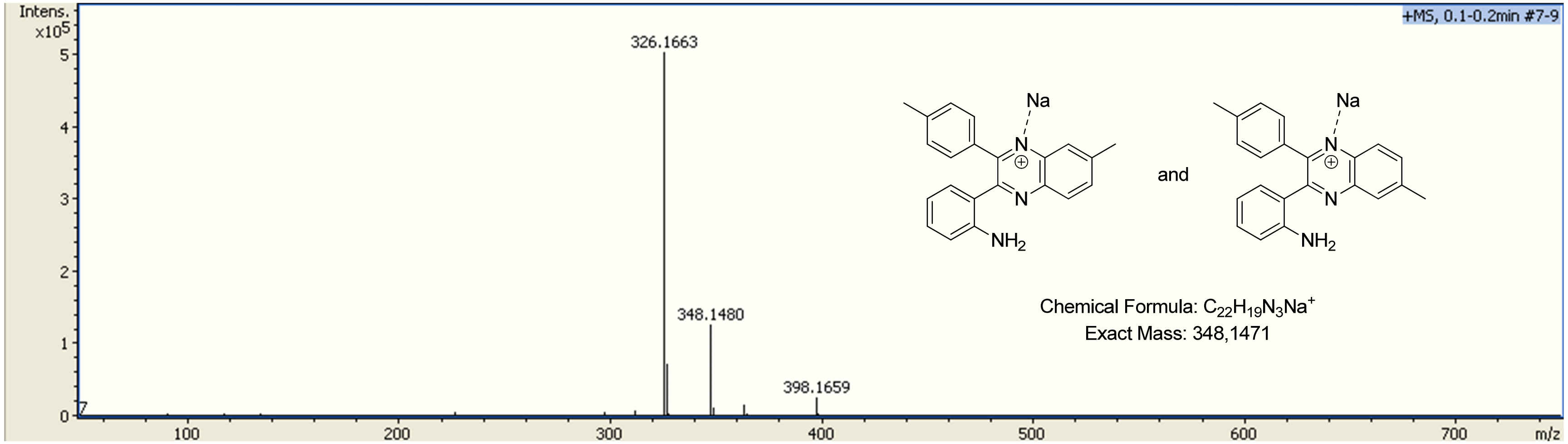

Figure S36. HRMS spectral chart for **4bb+4’bb**

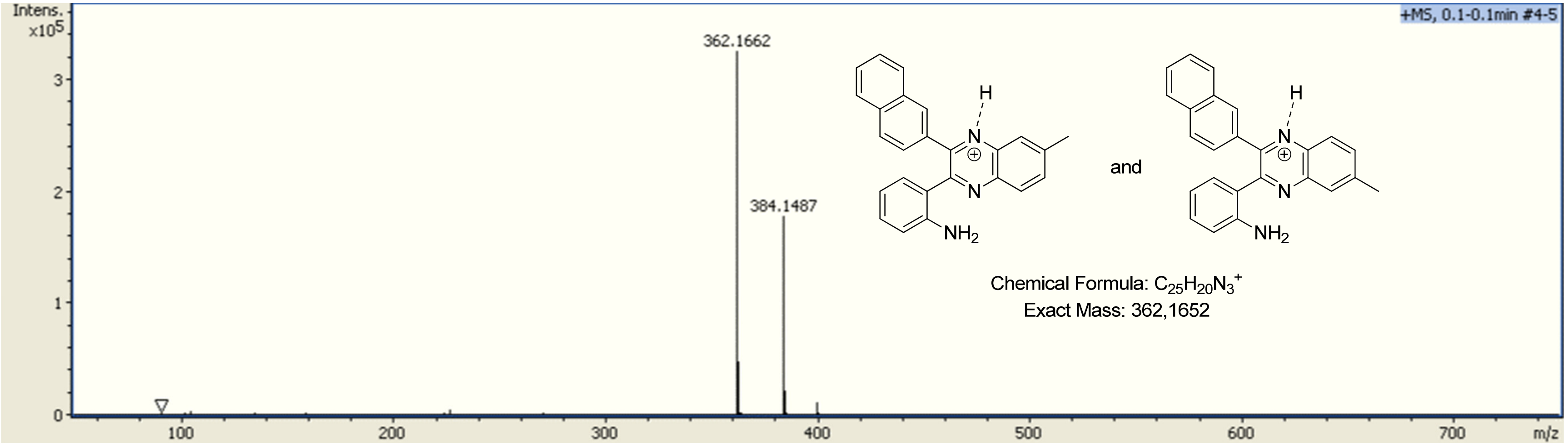

Figure S37. HRMS spectral chart for **4bc+4’bc**

X-Ray crystallography data

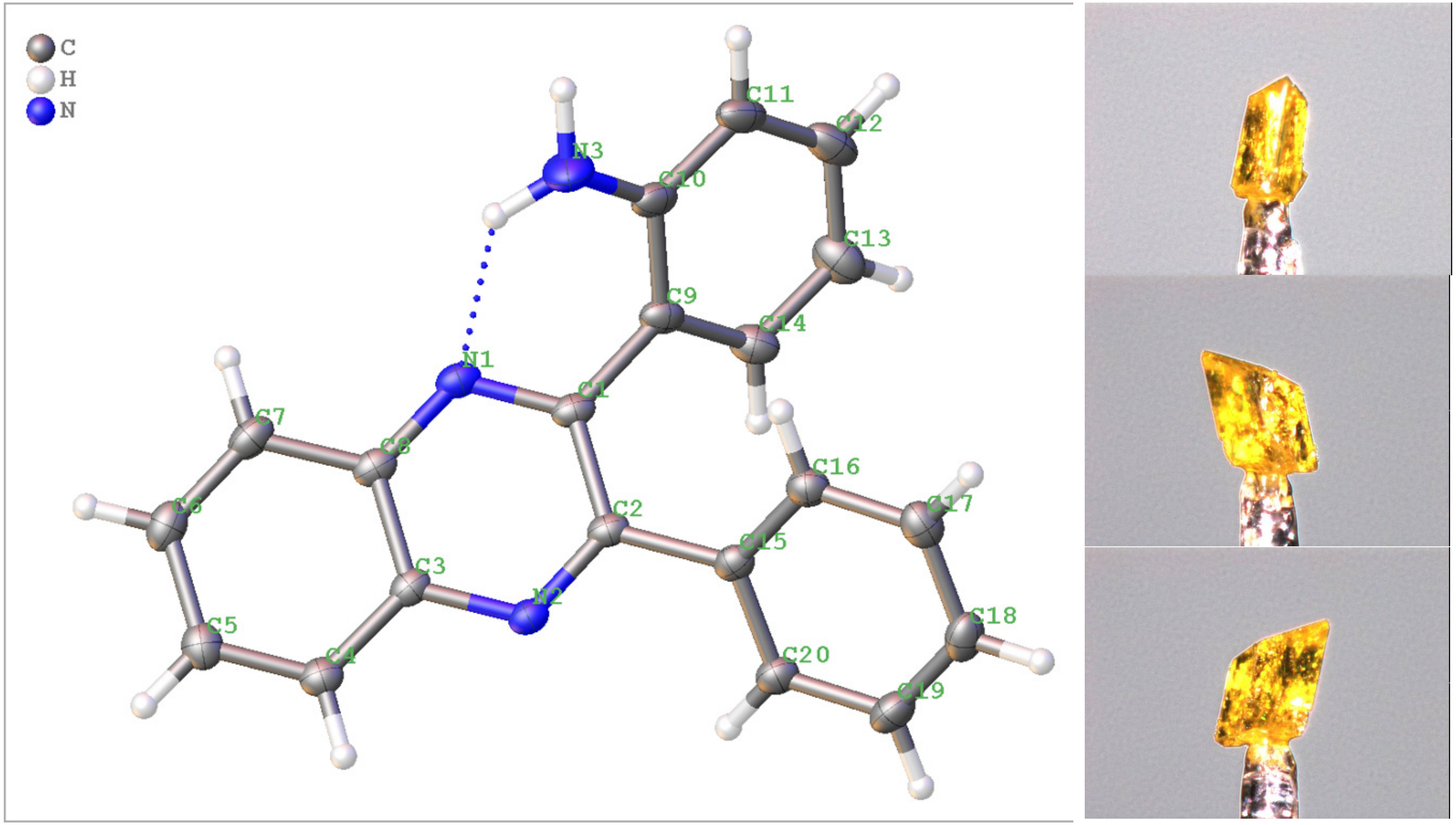

Figure S38. ORTEP drawing of the crystal structure showing 50% probability thermal ellipsoids (left, CCDC 2195374) and microphotography of the single crystal of compound **4aa** used for X-Ray diffraction analysis at the bottom.

Table S1 Crystal data and structure refinement for4aa.

Identification code	ANNA_KUR422_2
Empirical formula	$C_{20}H_{15}N_3$
Formula weight	297.35
Temperature/K	100.00(10)
Crystal system	monoclinic
Space group	$P2_1/n$
a/Å	14.4029(4)
b/Å	6.66847(16)
c/Å	16.3992(4)
α/°	90
β/°	103.136(2)
γ/°	90
Volume/Å^3	1533.86(7)
Z	4
ρ_{calc}g/cm^3	1.288
μ/mm^{-1}	0.607
F(000)	624.0
Crystal size/mm^3	0.478 × 0.279 × 0.188
Radiation	Cu Kα (λ = 1.54184)
2Θ range for data collection/°	7.382 to 152.812
Index ranges	-18 ≤ h ≤ 17, -8 ≤ k ≤ 8, -20 ≤ l ≤ 19
Reflections collected	16681
Independent reflections	3222 [R_{int} = 0.0303, R_{sigma} = 0.0184]
Data/restraints/parameters	3222/0/217
Goodness-of-fit on F^2	1.065
Final R indexes [I>=2σ (I)]	R_1 = 0.0441, wR_2 = 0.1182
Final R indexes [all data]	R_1 = 0.0459, wR_2 = 0.1199
Largest diff. peak/hole / e Å^{-3}	0.44/-0.21

Table S2 Fractional Atomic Coordinates (×10^4) and Equivalent Isotropic Displacement Parameters (Å^2×10^3) for 4aa. U_{eq} is defined as 1/3 of of the trace of the orthogonalised U_{IJ} tensor.

Atom	*x*	*y*	*z*	U(eq)
N1	3567.9(7)	7549.0(14)	3813.0(6)	20.4(2)
N2	4091.5(7)	7452.9(13)	5571.4(6)	18.1(2)
C8	4501.2(9)	7462.3(16)	4222.0(7)	19.5(3)
C2	3188.7(8)	7400.0(16)	5165.7(7)	17.8(2)
C1	2915.9(8)	7569.1(16)	4266.6(7)	18.7(2)
C3	4765.3(8)	7506.2(16)	5107.3(7)	18.1(2)
C15	2488.3(8)	7081.5(17)	5695.1(7)	19.1(2)
C4	5743.0(9)	7507.9(16)	5513.5(8)	21.1(3)
C16	1723.8(8)	5749.9(18)	5458.8(7)	22.3(3)
C7	5218.3(9)	7369.2(17)	3757.7(8)	23.7(3)
C5	6421.1(9)	7452.3(17)	5048.8(8)	23.4(3)
N3	2040.9(9)	5093(2)	2851.1(7)	35.0(3)
C20	2633.8(8)	8027.2(18)	6471.8(7)	22.0(3)
C9	1911.2(8)	7831.6(18)	3808.7(7)	21.2(3)
C6	6157.0(9)	7373.5(18)	4166.4(8)	25.1(3)
C18	1270.9(9)	6348(2)	6764.5(8)	27.2(3)
C17	1124.1(9)	5377.1(19)	5995.5(8)	26.1(3)
C10	1519.4(9)	6634(2)	3101.8(7)	25.1(3)
C12	22.7(9)	8408(2)	2972.1(8)	32.4(3)
C19	2022.2(9)	7681(2)	7000.0(8)	26.9(3)
C14	1348.4(9)	9271.1(18)	4078.2(8)	25.4(3)
C11	563.9(9)	6956(2)	2698.2(7)	29.4(3)
C13	406.2(9)	9582(2)	3667.6(9)	31.6(3)

Table S3 Anisotropic Displacement Parameters (Å^2×10^3) for 4aa. The Anisotropic displacement factor exponent takes the form: -2π^2[h^{2}a*^{2}U$_{11}$+2hka*b*U$_{12}$+…].

Atom	U_{11}	U_{22}	U_{33}	U_{23}	U_{13}	U_{12}
N1	28.9(5)	16.1(5)	15.7(5)	0.3(3)	4.4(4)	-0.6(4)
N2	23.4(5)	14.9(5)	16.1(5)	-0.5(3)	4.6(4)	0.6(4)
C8	28.3(6)	12.9(5)	17.5(5)	1.4(4)	5.8(4)	1.0(4)
C2	24.6(6)	12.9(5)	15.4(5)	-0.7(4)	3.7(4)	0.6(4)
C1	26.4(6)	13.6(5)	15.4(5)	-0.5(4)	3.3(4)	-1.3(4)
C3	25.3(6)	12.4(5)	17.6(5)	0.3(4)	6.7(4)	0.8(4)
C15	22.2(5)	17.9(5)	16.6(5)	2.7(4)	3.3(4)	3.3(4)
C4	26.6(6)	15.8(6)	20.5(6)	-0.7(4)	4.7(5)	-0.1(4)
C16	24.1(5)	21.6(6)	20.3(5)	1.0(4)	3.2(4)	0.8(4)
C7	35.7(7)	18.5(6)	19.4(5)	1.9(4)	11.3(5)	2.1(5)
C5	24.5(6)	16.3(6)	30.3(6)	0.4(4)	8.1(5)	0.4(4)
N3	36.0(6)	40.8(7)	27.5(6)	-15.9(5)	5.7(5)	-6.1(5)
C20	26.2(6)	21.9(6)	17.9(5)	1.3(4)	4.8(4)	0.7(5)
C9	26.0(6)	20.7(6)	15.4(5)	3.3(4)	1.6(4)	-3.6(4)
C6	32.3(6)	18.2(6)	28.6(6)	2.5(5)	15.0(5)	1.7(5)
C18	27.9(6)	30.6(7)	26.0(6)	8.9(5)	12.1(5)	4.8(5)
C17	23.2(6)	24.4(6)	30.1(6)	5.7(5)	5.0(5)	-0.3(5)
C10	30.6(6)	29.5(6)	14.9(5)	1.3(5)	4.6(4)	-8.3(5)
C12	25.1(6)	37.6(8)	29.2(6)	11.4(6)	-4.9(5)	-6.5(5)
C19	33.6(6)	29.5(7)	18.9(5)	0.9(5)	8.9(5)	2.8(5)
C14	28.2(6)	19.9(6)	25.0(6)	0.9(5)	-0.8(5)	-1.4(5)
C11	31.4(6)	37.4(7)	17.1(5)	2.7(5)	0.7(5)	-13.1(6)
C13	28.3(6)	25.4(6)	37.5(7)	4.7(5)	-0.2(5)	2.4(5)

Table S4 Bond Lengths for 4aa.

Atom	Atom	Length/Å	Atom	Atom	Length/Å
N1	C8	1.3603(15)	C16	C17	1.3887(17)
N1	C1	1.3239(15)	C7	C6	1.3657(18)
N2	C2	1.3196(15)	C5	C6	1.4111(18)
N2	C3	1.3630(15)	N3	C10	1.3887(18)
C8	C3	1.4151(16)	C20	C19	1.3880(17)
C8	C7	1.4170(16)	C9	C10	1.4153(16)
C2	C1	1.4415(15)	C9	C14	1.3916(18)
C2	C15	1.4881(16)	C18	C17	1.3904(18)
C1	C9	1.4814(16)	C18	C19	1.3856(19)
C3	C4	1.4144(16)	C10	C11	1.4016(18)
C15	C16	1.3990(16)	C12	C11	1.381(2)
C15	C20	1.3937(16)	C12	C13	1.390(2)
C4	C5	1.3691(17)	C14	C13	1.3867(17)

Table S5 Bond Angles for 4aa.

Atom	Atom	Atom	Angle/˚	Atom	Atom	Atom	Angle/˚
C1	N1	C8	118.10(10)	C17	C16	C15	120.12(11)
C2	N2	C3	117.66(10)	C6	C7	C8	119.82(11)
N1	C8	C3	120.67(11)	C4	C5	C6	120.77(12)
N1	C8	C7	119.75(11)	C19	C20	C15	120.67(11)
C3	C8	C7	119.57(11)	C10	C9	C1	120.67(11)
N2	C2	C1	121.46(11)	C14	C9	C1	119.53(10)
N2	C2	C15	115.51(10)	C14	C9	C10	119.79(11)
C1	C2	C15	123.00(10)	C7	C6	C5	120.63(11)
N1	C1	C2	120.71(11)	C19	C18	C17	119.98(11)
N1	C1	C9	116.89(10)	C16	C17	C18	120.22(11)
C2	C1	C9	122.38(10)	N3	C10	C9	121.21(11)
N2	C3	C8	120.85(11)	N3	C10	C11	120.85(11)

Table S5 Bond Angles for 4aa.

Atom	Atom	Atom	Angle/°	Atom	Atom	Atom	Angle/°
N2	C3	C4	119.74(10)	C11	C10	C9	117.78(12)
C4	C3	C8	119.33(11)	C11	C12	C13	120.89(12)
C16	C15	C2	121.61(10)	C18	C19	C20	119.93(12)
C20	C15	C2	119.18(10)	C13	C14	C9	121.69(12)
C20	C15	C16	119.06(11)	C12	C11	C10	121.37(12)
C5	C4	C3	119.85(11)	C14	C13	C12	118.47(13)

Table S6 Torsion Angles for 4aa.

A	B	C	D	Angle/°	A	B	C	D	Angle/°
N1	C8	C3	N2	5.99(16)	C1	C9	C10	C11	179.90(11)
N1	C8	C3	C4	-177.08(10)	C1	C9	C14	C13	179.59(11)
N1	C8	C7	C6	177.05(10)	C3	N2	C2	C1	-5.32(15)
N1	C1	C9	C10	49.82(15)	C3	N2	C2	C15	172.52(9)
N1	C1	C9	C14	-130.22(12)	C3	C8	C7	C6	-1.64(17)
N2	C2	C1	N1	7.91(16)	C3	C4	C5	C6	-0.62(17)
N2	C2	C1	C9	-170.24(10)	C15	C2	C1	N1	-169.78(10)
N2	C2	C15	C16	-136.15(11)	C15	C2	C1	C9	12.07(16)
N2	C2	C15	C20	39.39(15)	C15	C16	C17	C18	1.01(18)
N2	C3	C4	C5	176.49(10)	C15	C20	C19	C18	1.47(19)
C8	N1	C1	C2	-3.11(15)	C4	C5	C6	C7	0.60(18)
C8	N1	C1	C9	175.13(10)	C16	C15	C20	C19	-0.96(18)
C8	C3	C4	C5	-0.48(16)	C7	C8	C3	N2	-175.33(10)
C8	C7	C6	C5	0.55(18)	C7	C8	C3	C4	1.60(16)
C2	N2	C3	C8	-1.33(15)	N3	C10	C11	C12	176.25(12)

Table S6 Torsion Angles for 4aa.

A	B	C	D	Angle/°	A	B	C	D	Angle/°
C2	N2	C3	C4	-178.25(10)	C20	C15	C16	C17	-0.28(17)
C2	C1	C9	C10	-131.97(12)	C9	C10	C11	C12	0.75(18)
C2	C1	C9	C14	47.99(16)	C9	C14	C13	C12	0.3(2)
C2	C15	C16	C17	175.26(10)	C17	C18	C19	C20	-0.73(19)
C2	C15	C20	C19	-176.61(11)	C10	C9	C14	C13	-0.45(19)
C1	N1	C8	C3	-3.47(16)	C19	C18	C17	C16	-0.51(19)
C1	N1	C8	C7	177.85(10)	C14	C9	C10	N3	-175.55(12)
C1	C2	C15	C16	41.66(16)	C14	C9	C10	C11	-0.06(17)
C1	C2	C15	C20	-142.80(11)	C11	C12	C13	C14	0.4(2)
C1	C9	C10	N3	4.41(18)	C13	C12	C11	C10	-0.9(2)

Table S7 Hydrogen Atom Coordinates (Å×10^4) and Isotropic Displacement Parameters (Å^2×10^3) for 4aa.

Atom	*x*	*y*	*z*	U(eq)
H4	5924.88	7546.56	6094.91	25
H16	1617.07	5113.29	4941.22	27
H7	5050.21	7305.33	3176.17	28
H5	7063.39	7466.79	5317.77	28
H20	3145.75	8899.42	6637.73	26
H6	6626.53	7323.89	3860.23	30
H18	865.03	6103.06	7120.73	33
H17	622.61	4474.96	5840.11	31
H12	-606.95	8603.68	2687.41	39
H19	2116.66	8342.12	7511.55	32
H14	1611.04	10044.52	4545.63	31

Table S7 Hydrogen Atom Coordinates (Å×10^4^) and Isotropic Displacement Parameters (Å^2^×10^3^) for 4aa.

Atom	*x*	*y*	*z*	U(eq)
H11	288.84	6176.47	2236.25	35
H13	39.2	10553.56	3853.23	38
H3A	2713(14)	5310(30)	2969(12)	51(5)
H3B	1762(12)	4430(30)	2345(11)	44(5)

Experimental

Single crystals of $C_{20}H_{15}N_3$ **4aa** were obtained by slow evaporation of saturated solution in EtOAc. A suitable crystal was selected and mounted on the glass stick by acrylic glue on a SuperNova, Dual, Cu at home/near**, AtlasS2 diffractometer**. The crystal was kept at 100.00(10) K during data collection. Using Olex2 [S1], the structure was solved with the olex2.solve [S2] structure solution program using Charge Flipping and refined with the SHELXL [S3] refinement package using Least Squares minimisation.

Crystal structure determination of 4aa

Crystal Data for $C_{20}H_{15}N_3$ (M =297.35 g/mol): monoclinic, space group P2$_1$/n (no. 14), a = 14.4029(4) Å, b = 6.66847(16) Å, c = 16.3992(4) Å, β = 103.136(2)°, V = 1533.86(7) Å^3, Z = 4, T = 100.00(10) K, μ(Cu Kα) = 0.607 mm^{-1}, $Dcalc$ = 1.288 g/cm^3, 16681 reflections measured (7.382° ≤ 2Θ ≤ 152.812°), 3222 unique (R_{int} = 0.0303, R_{sigma} = 0.0184) which were used in all calculations. The final R_1 was 0.0441 (I > 2σ(I)) and wR_2 was 0.1199 (all data).

Refinement model description

Number of restraints - 0, number of constraints - unknown.

Details:

```
1. Fixed Uiso
 At 1.2 times of:
  All C(H) groups
2.a Aromatic/amide H refined with riding coordinates:
 C4(H4), C16(H16), C7(H7), C5(H5), C20(H20), C6(H6), C18(H18), C17(H17),
 C12(H12), C19(H19), C14(H14), C11(H11), C13(H13)
```

This report has been created with Olex2, compiled on 2020.11.12 svn.r5f609507 for OlexSys. Please let us know if there are any errors or if you would like to have additional features.

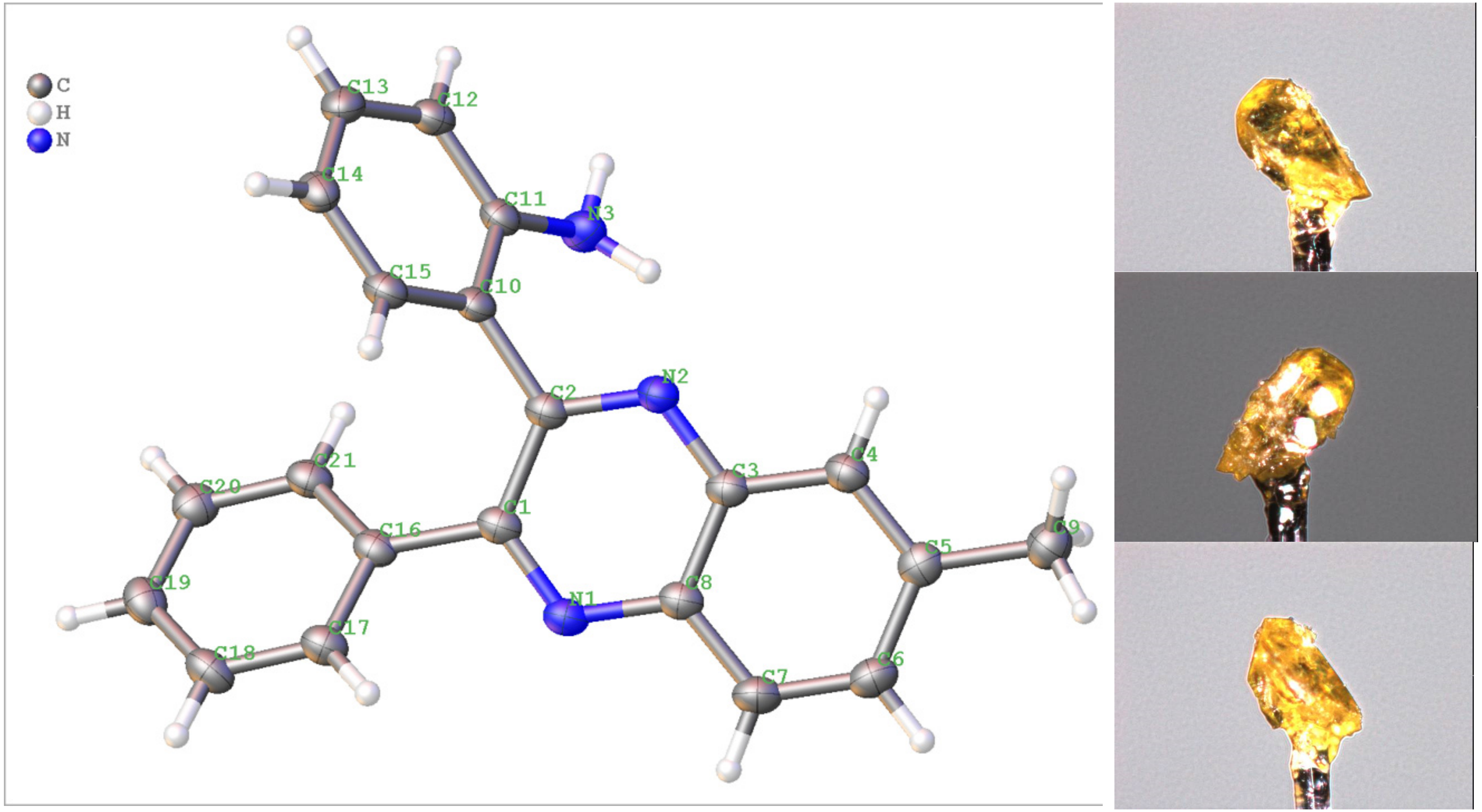

Figure S39. ORTEP drawing of the crystal structure showing 50% probability thermal ellipsoids (left, CCDC 2195382) and microphotography of the single crystal of compound **4'ba** used for X-Ray diffraction analysis at the bottom.

Table S8 Crystal data and structure refinement for ANNA_KUR432_2.

Identification code	ANNA_KUR432_2
Empirical formula	$C_{21}H_{17}N_3$
Formula weight	311.38
Temperature/K	100.00(10)
Crystal system	monoclinic
Space group	$P2_1/n$
a/Å	11.67181(16)
b/Å	7.46450(9)
c/Å	18.9827(3)
α/°	90
β/°	106.0997(14)
γ/°	90
Volume/Å^3	1588.99(4)
Z	4
ρ_{calc}g/cm^3	1.302
μ/mm^{-1}	0.609
F(000)	656.0
Crystal size/mm^3	0.541 × 0.328 × 0.253
Radiation	Cu Kα (λ = 1.54184)
2Θ range for data collection/°	8.028 to 152.66
Index ranges	$-14 \leq h \leq 14$, $-9 \leq k \leq 9$, $-23 \leq l \leq 23$
Reflections collected	16948
Independent reflections	3324 [R_{int} = 0.0207, R_{sigma} = 0.0133]
Data/restraints/parameters	3324/0/227
Goodness-of-fit on F^2	1.045
Final R indexes [I>=2σ (I)]	R_1 = 0.0379, wR_2 = 0.0997
Final R indexes [all data]	R_1 = 0.0393, wR_2 = 0.1008
Largest diff. peak/hole / e Å^{-3}	0.39/-0.25

Table S9 Fractional Atomic Coordinates (×10^4) and Equivalent Isotropic Displacement Parameters (Å^2×10^3) for 4'ba. U_{eq} is defined as 1/3 of of the trace of the orthogonalised U_{IJ} tensor.

Atom	*x*	*y*	*z*	U(eq)
N2	3613.8(8)	2761.0(13)	3659.2(5)	23.9(2)
N1	5911.6(8)	2317.9(13)	4613.0(5)	24.0(2)
N3	3395.8(9)	5476.5(14)	2477.9(6)	29.7(2)
C4	2821.2(10)	3086.1(15)	4685.4(6)	25.7(2)
C2	4546.2(10)	2539.4(14)	3402.3(6)	22.5(2)
C11	3662.5(9)	3906.4(16)	2158.6(6)	23.9(2)
C17	7761.3(10)	1100.1(15)	4027.0(6)	26.3(2)
C18	8800.1(10)	961.7(16)	3809.7(7)	30.0(3)
C10	4276.8(9)	2478.4(15)	2589.9(6)	22.9(2)
C8	4955.9(10)	2564.3(14)	4879.8(6)	23.5(2)
C15	4613.0(10)	997.5(16)	2249.3(6)	26.1(2)
C14	4372.4(10)	897.4(17)	1493.1(6)	28.8(3)
C16	6815.6(10)	2165.0(15)	3630.6(6)	22.9(2)
C1	5732.9(10)	2336.4(14)	3893.5(6)	22.7(2)
C12	3406.0(10)	3768.7(17)	1395.0(6)	27.0(3)
C3	3797.8(10)	2805.5(15)	4399.7(6)	23.2(2)
C5	2965.9(11)	3181.3(15)	5425.9(6)	26.3(2)
C21	6948.7(10)	3110.9(16)	3024.6(6)	25.7(2)
C20	7990.2(10)	2984.7(17)	2812.0(6)	28.6(3)
C9	1906.6(11)	3553.0(18)	5717.4(7)	31.5(3)
C7	5097.0(11)	2636.4(16)	5644.7(6)	28.1(3)
C19	8918.1(10)	1897.6(17)	3203.0(7)	29.7(3)
C6	4134.1(11)	2945.7(16)	5906.5(6)	28.5(3)
C13	3755.3(10)	2288.6(18)	1067.2(6)	29.1(3)

Table S10 Anisotropic Displacement Parameters (Å^2×10^3) for 4'ba. The Anisotropic displacement factor exponent takes the form: -2π^2[h^{2}a*^{2}U$_{11}$+2hka*b*U$_{12}$+…].

Atom	U_{11}	U_{22}	U_{33}	U_{23}	U_{13}	U_{12}
N2	22.3(4)	23.6(5)	23.5(5)	-1.2(4)	2.6(4)	1.0(4)
N1	23.2(5)	20.7(5)	25.1(5)	1.2(4)	1.6(4)	-0.8(4)
N3	31.3(5)	28.5(5)	27.4(5)	2.0(4)	4.8(4)	8.3(4)
C4	24.5(5)	22.9(5)	27.2(6)	-0.9(4)	3.0(4)	1.0(4)
C2	21.8(5)	18.9(5)	24.4(5)	-0.3(4)	2.4(4)	0.4(4)
C11	17.2(5)	26.1(6)	26.6(5)	-0.9(4)	3.0(4)	-0.4(4)
C17	24.0(5)	21.9(5)	30.2(6)	1.6(4)	2.7(4)	-0.8(4)
C18	22.4(5)	24.6(6)	40.0(7)	0.2(5)	3.4(5)	1.3(4)
C10	16.8(5)	25.9(6)	23.6(5)	-0.8(4)	1.6(4)	-1.3(4)
C8	24.2(5)	18.3(5)	25.3(6)	0.4(4)	2.4(4)	-1.3(4)
C15	20.6(5)	26.2(6)	28.1(6)	-1.1(4)	0.9(4)	0.9(4)
C14	24.6(5)	31.0(6)	28.6(6)	-7.5(5)	3.6(4)	0.3(5)
C16	20.4(5)	19.3(5)	26.1(5)	-2.5(4)	1.6(4)	-2.6(4)
C1	22.2(5)	16.7(5)	26.2(5)	0.6(4)	1.9(4)	-0.9(4)
C12	20.7(5)	30.7(6)	26.5(6)	2.9(5)	1.2(4)	0.6(4)
C3	24.8(5)	18.9(5)	23.6(5)	-1.4(4)	2.7(4)	-0.5(4)
C5	31.8(6)	18.0(5)	28.6(6)	-0.6(4)	7.8(5)	-1.0(4)
C21	22.2(5)	23.9(5)	27.6(6)	-0.2(4)	1.2(4)	-2.5(4)
C20	28.0(6)	27.2(6)	29.4(6)	-2.1(5)	6.0(5)	-6.7(5)
C9	35.1(6)	29.2(6)	30.9(6)	-1.3(5)	10.4(5)	-0.1(5)
C7	28.5(6)	26.8(6)	24.9(6)	2.3(4)	0.5(4)	-0.9(5)
C19	23.4(5)	27.4(6)	38.5(6)	-6.2(5)	8.8(5)	-4.5(5)
C6	35.3(6)	25.5(6)	22.7(5)	1.0(4)	4.6(5)	-2.5(5)
C13	24.8(5)	37.5(7)	22.6(5)	-3.5(5)	2.6(4)	-2.2(5)

Table S11 Bond Lengths for 4'ba.

Atom	Atom	Length/Å	Atom	Atom	Length/Å
N2	C2	1.3206(14)	C10	C15	1.3909(16)
N2	C3	1.3622(14)	C8	C3	1.4166(15)
N1	C8	1.3593(15)	C8	C7	1.4162(16)
N1	C1	1.3233(14)	C15	C14	1.3861(16)
N3	C11	1.3938(15)	C14	C13	1.3885(18)
C4	C3	1.4073(16)	C16	C1	1.4873(15)
C4	C5	1.3703(16)	C16	C21	1.3947(16)
C2	C10	1.4867(15)	C12	C13	1.3838(17)
C2	C1	1.4473(15)	C5	C9	1.5137(16)
C11	C10	1.4124(16)	C5	C6	1.4256(17)
C11	C12	1.4002(16)	C21	C20	1.3862(16)
C17	C18	1.3886(16)	C20	C19	1.3916(18)
C17	C16	1.3985(16)	C7	C6	1.3685(17)
C18	C19	1.3861(18)			

Table S12 Bond Angles for 4'ba.

Atom	Atom	Atom	Angle/°	Atom	Atom	Atom	Angle/°
C2	N2	C3	118.29(9)	C17	C16	C1	119.05(10)
C1	N1	C8	118.19(9)	C21	C16	C17	118.66(10)
C5	C4	C3	121.36(10)	C21	C16	C1	122.18(10)
N2	C2	C10	115.34(9)	N1	C1	C2	120.98(10)
N2	C2	C1	120.97(10)	N1	C1	C16	116.11(9)
C1	C2	C10	123.67(10)	C2	C1	C16	122.90(10)
N3	C11	C10	121.42(10)	C13	C12	C11	121.27(11)
N3	C11	C12	120.16(10)	N2	C3	C4	119.25(10)
C12	C11	C10	118.23(10)	N2	C3	C8	120.67(10)
C18	C17	C16	120.42(11)	C4	C3	C8	120.09(10)
C19	C18	C17	120.36(11)	C4	C5	C9	120.24(10)

Table S12 Bond Angles for 4'ba.

Atom	Atom	Atom	Angle/°	Atom	Atom	Atom	Angle/°
C11	C10	C2	120.36(10)	C4	C5	C6	118.29(11)
C15	C10	C2	120.05(10)	C6	C5	C9	121.46(10)
C15	C10	C11	119.58(10)	C20	C21	C16	120.87(11)
N1	C8	C3	120.84(10)	C21	C20	C19	120.00(11)
N1	C8	C7	120.86(10)	C6	C7	C8	120.33(11)
C7	C8	C3	118.27(11)	C18	C19	C20	119.67(11)
C14	C15	C10	121.47(11)	C7	C6	C5	121.63(11)
C15	C14	C13	119.08(11)	C12	C13	C14	120.35(11)

Table S13 Torsion Angles for 4'ba.

A	B	C	D	Angle/°	A	B	C	D	Angle/°
N2	C2	C10	C11	56.12(14)	C8	N1	C1	C2	-2.50(15)
N2	C2	C10	C15	-122.87(11)	C8	N1	C1	C16	176.64(9)
N2	C2	C1	N1	1.88(16)	C8	C7	C6	C5	0.79(18)
N2	C2	C1	C16	-177.19(10)	C15	C14	C13	C12	1.20(18)
N1	C8	C3	N2	1.28(16)	C16	C17	C18	C19	0.90(18)
N1	C8	C3	C4	-179.03(10)	C16	C21	C20	C19	0.28(17)
N1	C8	C7	C6	177.78(11)	C1	N1	C8	C3	0.99(15)
N3	C11	C10	C2	6.35(16)	C1	N1	C8	C7	-177.23(10)
N3	C11	C10	C15	-174.66(10)	C1	C2	C10	C11	-125.16(12)
N3	C11	C12	C13	174.29(11)	C1	C2	C10	C15	55.86(15)
C4	C5	C6	C7	0.18(17)	C1	C16	C21	C20	177.11(10)
C2	N2	C3	C4	178.39(10)	C12	C11	C10	C2	-178.59(10)

Table S13 Torsion Angles for 4'ba.

A	B	C	D	Angle/°	A	B	C	D	Angle/°
C2	N2	C3	C8	-1.91(16)	C12	C11	C10	C15	0.40(16)
C2	C10	C15	C14	179.84(10)	C3	N2	C2	C10	179.17(9)
C11	C10	C15	C14	0.84(17)	C3	N2	C2	C1	0.41(15)
C11	C12	C13	C14	0.03(18)	C3	C4	C5	C9	177.72(11)
C17	C18	C19	C20	0.19(18)	C3	C4	C5	C6	-1.47(17)
C17	C16	C1	N1	34.11(14)	C3	C8	C7	C6	-0.49(17)
C17	C16	C1	C2	-146.77(11)	C5	C4	C3	N2	-178.52(10)
C17	C16	C21	C20	0.79(17)	C5	C4	C3	C8	1.78(17)
C18	C17	C16	C1	-177.82(10)	C21	C16	C1	N1	-142.20(11)
C18	C17	C16	C21	-1.37(17)	C21	C16	C1	C2	36.92(16)
C10	C2	C1	N1	-176.78(10)	C21	C20	C19	C18	-0.77(18)
C10	C2	C1	C16	4.15(16)	C9	C5	C6	C7	-178.99(11)
C10	C11	C12	C13	-0.83(16)	C7	C8	C3	N2	179.54(10)
C10	C15	C14	C13	-1.65(17)	C7	C8	C3	C4	-0.76(16)

Table S14 Hydrogen Atom Coordinates (Å×10^4) and Isotropic Displacement Parameters (Å^2×10^3) for 4'ba.

Atom	*x*	*y*	*z*	U(eq)
H4	2061.8	3209.49	4365.05	31
H17	7694.08	480.75	4438.75	32
H18	9420.01	237.09	4072.62	36
H15	5008.04	53.61	2535.23	31
H14	4620.89	-88.04	1274.06	35
H12	2992.86	4689.69	1102.55	32
H21	6330.68	3835.73	2759.64	31
H20	8068.83	3626.63	2408.06	34

Table S14 Hydrogen Atom Coordinates (Å×10[4]) and Isotropic Displacement Parameters (Å[2]×10[3]) for 4'ba.

Atom	*x*	*y*	*z*	U(eq)
H9A	1789.73	4823.22	5736.02	47
H9B	2052.41	3059.14	6201.21	47
H9C	1206	3012.24	5399.88	47
H7	5847.65	2472.86	5970.68	34
H19	9613.99	1799.41	3057.91	36
H6	4244.65	3003.6	6410.32	34
H13	3575.87	2225.39	558.91	35
H3A	2881(14)	6210(20)	2158(9)	40(4)
H3B	3174(15)	5300(20)	2903(9)	47(5)

Experimental

Single crystals of $C_{21}H_{17}N_3$ 4'ba were obtained by slow evaporation of EtOAc solution. A suitable crystal was selected and mounted on the glass stick by acrylic glue on a **SuperNova, Dual, Cu at home/near, AtlasS2** diffractometer. The crystal was kept at 100.00(10) K during data collection. Using Olex2 [S1], the structure was solved with the olex2.solve [S2] structure solution program using Charge Flipping and refined with the SHELXL [S3] refinement package using Least Squares minimisation.

Crystal structure determination of 4'ba

Crystal Data for $C_{21}H_{17}N_3$ (M =311.38 g/mol): monoclinic, space group P2$_1$/n (no. 14), a = 11.67181(16) Å, b = 7.46450(9) Å, c = 18.9827(3) Å, β = 106.0997(14)°, V = 1588.99(4) Å^3, Z = 4, T = 100.00(10) K, μ(Cu Kα) = 0.609 mm^{-1}, $Dcalc$ = 1.302 g/cm^3, 16948 reflections measured (8.028° ≤ 2Θ ≤ 152.66°), 3324 unique (R_{int} = 0.0207, R_{sigma} = 0.0133) which were used in all calculations. The final R_1 was 0.0379 (I > 2σ(I)) and wR_2 was 0.1008 (all data).

Refinement model description

Number of restraints - 0, number of constraints - unknown.

Details:

```
1. Fixed Uiso
 At 1.2 times of:
  All C(H) groups
 At 1.5 times of:
  All C(H,H,H) groups
2.a Aromatic/amide H refined with riding coordinates:
 C4(H4), C17(H17), C18(H18), C15(H15), C14(H14), C12(H12), C21(H21), C20(H20),
 C7(H7), C19(H19), C6(H6), C13(H13)
2.b Idealised Me refined as rotating group:
 C9(H9A,H9B,H9C)
```

This report has been created with Olex2, compiled on 2020.11.12 svn.r5f609507 for OlexSys. Please let us know if there are any errors or if you would like to have additional features.

References

S1. Dolomanov, O.V., Bourhis, L.J., Gildea, R.J, Howard, J.A.K. & Puschmann, H. (2009), *J. Appl. Cryst*. 42, 339-341.

S2. Sheldrick, G.M. (2008). *Acta Cryst*. A64, 112-122.

S3. Sheldrick, G.M. (2015). *Acta Cryst*. C71, 3-8.
